# Isothiourea-Catalyzed Enantioselective Michael Addition of Malonates to α,β-Unsaturated Aryl Esters

**DOI:** 10.1021/acs.orglett.2c01486

**Published:** 2022-06-02

**Authors:** Jiufeng Wu, Claire M. Young, Amy A. Watts, Alexandra M. Z. Slawin, Gregory R. Boyce, Michael Bühl, Andrew D. Smith

**Affiliations:** † EaStCHEM, School of Chemistry, University of St Andrews, North Haugh, St Andrews, Fife KY16 9ST, United Kingdom; ‡ Department of Chemistry and Physics, Florida Gulf Coast University, Fort Myers, Florida 33965, United States

## Abstract

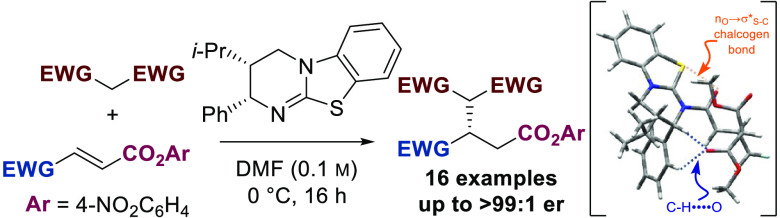

An enantioselective Michael addition of malonates to α,β-unsaturated *para*-nitrophenyl esters was achieved using the Lewis basic isothiourea HyperBTM, giving excellent levels of product enantioselectivity (up to >99:1 enantiomeric ratio) in good yields and with complete regioselectivity (>20:1 regioselectivity ratio) in the presence of alternative (phenyl ketone and ethyl ester) Michael acceptors. Density functional theory calculations indicate that N-acylation is rate-limiting. This constitutes a rare example of a highly enantioselective addition of simple, readily available malonates to α,β-unsaturated esters.

The asymmetric Michael reaction is a powerful method for stereoselective C–C bond formation. While enantioselective catalytic Michael addition of carbon nucleophiles to α,β-unsaturated aldehydes, ketones, and alkylidene malonates are well-established,^1^ analogous enantioselective addition to α,β-unsaturated esters are rare. This is likely due to the low inherent electrophilicity of the carboxylic acid oxidation state compared to alternative Michael acceptors^2^ combined with the lack of enantiofacial discrimination. Despite these issues, several useful catalytic enantioselective additions have been achieved with highly reactive nucleophilic partners, including silyl ketene acetals,^3^ dihydropyrazol-3-ones,^4^ aryl boronic acids,^5^ thiols and amines,^6^ and Grignard reagents.^7^ However, the addition of less reactive, stabilized carbon nucleophiles, such as malonates, remains an unsolved challenge. The current state of the art was demonstrated by Nakamura and co-workers in 2016, who employed a chiral lithium binaphtholate complex **1** to promote the highly enantioselective addition of malonates to symmetric maleic esters,^8^ but this was limited by the lack of variability at the β position of the Michael acceptor (Scheme 1A). As a result of the importance of this bond disconnection, alternative enantioselective methods with a broad scope would be a welcome addition to the synthetic toolbox.

**Scheme 1:**
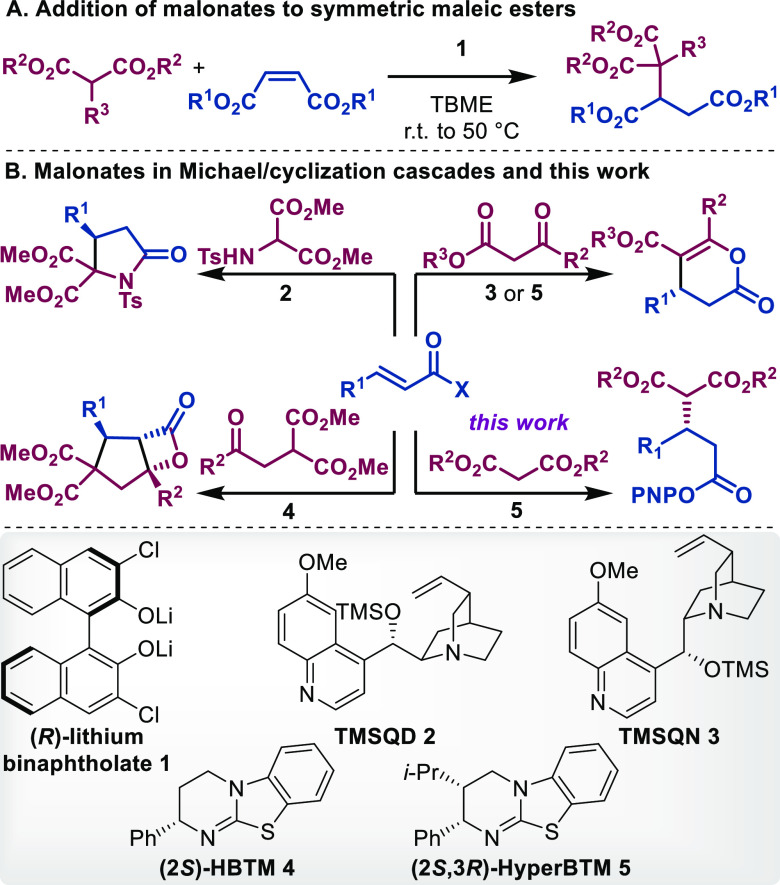
Selected Examples of Chiral Amine-Catalyzed Michael Reaction/Cyclization Cascades with Malonate Derivatives and Comparison to This Work

Chiral tertiary amines, such as chiral 4-dimethylaminopyridine (DMAP) derivatives,^9^ cinchona alkaloids,^10^ and isothioureas,^11^ are effective organocatalysts for inducing asymmetry in a variety of transformations with α,β-unsaturated carboxylic acid derivatives via chiral α,β-unsaturated *N*-acylammonium intermediates.^12^ This technique is frequently employed with bis-nucleophile coupling partners that rely upon an initial stereoselective conjugate addition followed by a second nucleophilic addition to achieve turnover of the chiral tertiary amine catalyst. Using this strategy, several methods have been developed employing an asymmetric Michael reaction with malonate derivatives followed by cyclization to release the organocatalysts, with instructive examples highlighted in Scheme 1B.

Romo and co-workers developed an elegant cinchona alkaloid **2**-catalyzed Michael reaction/proton transfer/lactamization cascade to provide lactams from aminomalonates and α,β-unsaturated acid chlorides (top left).^10^ The isothioureas, HBTM **4** and HyperBTM **5**, have been employed in cascade reactions, where an initial Michael reaction with β-ketoesters^10a,13^ (top right) and β-ketomalonates^14^ (bottom left) was followed by a cyclization event to release the catalyst and deliver δ- and β-lactones in high enantioselectivity, respectively. Building on this precedent and previous work that demonstrated the multifunctional nature of electron-deficient phenoxides as a leaving group and as a secondary nucleophile to achieve catalytic turnover in isothiourea catalysis,^15^ we posited that α,β-unsaturated *p*-nitrophenyl (PNP) esters would be able to perform the Michael addition reaction without the need for a pendent secondary nucleophile to achieve catalytic turnover. This PNP turnover strategy has previously been employed to promote the enantioselective nitronate addition to α,β-unsaturated PNP esters;^15a^ however, this process required nitroalkane to be used as a solvent or highly reactive silyl nitronates to be used as stoichiometric nucleophiles.^15b,16^ The use of dihydropyrazol-3-ones and 3-substituted oxindoles as *N*-heterocyclic enolates was also achieved through the aryloxide catalytic turnover.^4^ Herein, we report the HyperBTM-catalyzed addition of simple malonates and related derivatives to α,β-unsaturated aryl esters possessing a variety of electron-withdrawing β substituents under mild reaction conditions.

An examination to determine the most suitable reaction parameters began with an analysis of solvents and bases (Table 1). β-Trifluoromethyl α,β-unsaturated PNP ester **6** was reacted with dimethyl malonate **7** in the presence of 20 mol % HyperBTM **5** and 1 equiv of diisopropylethylamine in CH_2_Cl_2_ to provide the desired product with promising 62:38 enantiomeric ratio (er) (entry 1). Moving to more polar solvents, acetonitrile (MeCN) and *N*,*N*-dimethylformamide (DMF), provided higher yields (58 and 65%) and enantioselectivity (70:30 and 89:11) (entries 2 and 3). Gratifyingly, cooling the reaction in DMF to 0 °C increased the er to 90:10 (entry 4). Performing the reaction in the absence of external base at 0 °C (entry 5) provided high levels of enantioinduction (>99:1 er). Lowering the catalyst loading of compound **5** to 10 mol % (entry 6) resulted in similar enantioselectivity but a decreased yield. Attempting the reaction with (*R*)-BTM **9** furnished the desired Michael adduct in only 8% yield but with high 95:5 er (entry 7), while (*S*)-tetramisole **10** provided no desired product under the optimized reaction conditions (entry 8).

**Table 1:**
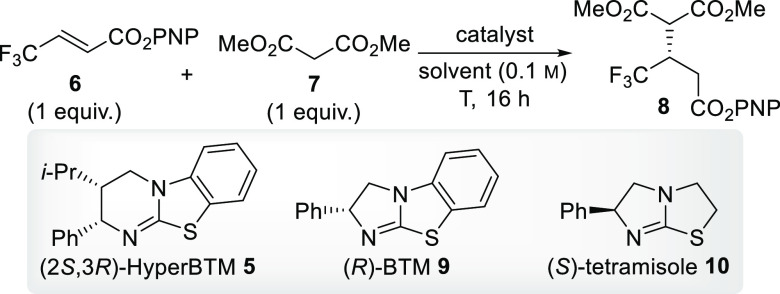
Reaction Optimization^a^

entry	solvent	catalyst (mol %)	base	*T* (°C)	yield (%)	er
1	CH_2_Cl_2_	**5** (20)	iPr_2_NEt	rt	30	62:38
2	MeCN	**5** (20)	iPr_2_NEt	rt	58	70:30
3	DMF	**5** (20)	iPr_2_NEt	rt	65	89:11
4	DMF	**5** (20)	iPr_2_NEt	0	73	90:10
5	DMF	**5** (20)		0	66	>99:1
6	DMF	**5** (10)		0	47	>99:1
7	DMF	**9** (20)		0	8	96:4
8	DMF	**10** (20)		0	0	

a All yields are isolated yields after purification by column chromatography. Enantiomeric ratios are determined by high-performance liquid chromatography (HPLC) analysis on a chiral stationary phase. PNP = *p*-nitrophenyl.

With the optimized conditions in hand, the steric and electronic parameters of the process were investigated. A variety of α,β-unsaturated aryl esters with electron-withdrawing β substituents were subjected to the optimized reaction conditions, with the results presented in Scheme 2.

**Scheme 2:**
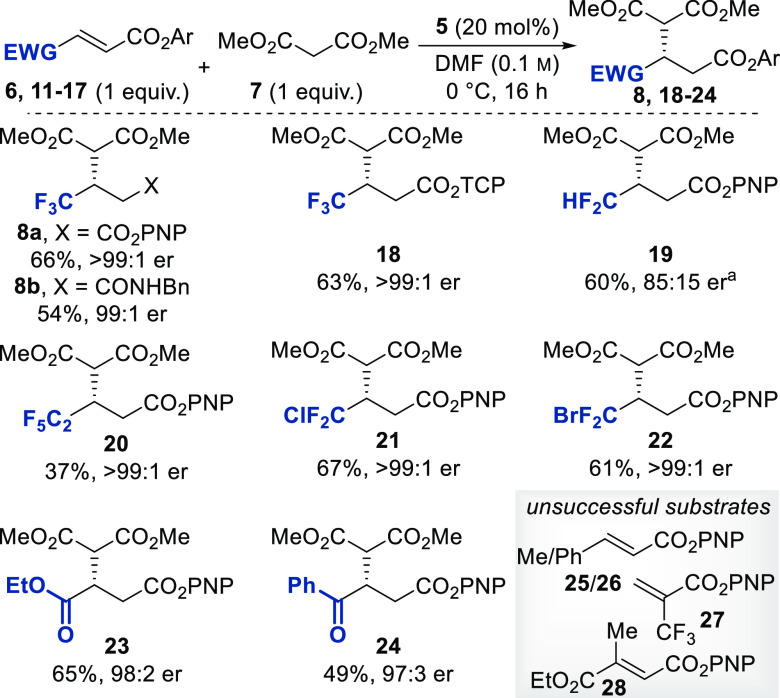
Scope and Limitations of Dimethyl Malonate Addition to α,β-Unsaturated Esters (2*R,*3*S*)-HyperBTM was used, and the product has the opposite absolute configuration to that shown. All yields are isolated yields after purification by column chromatography. Enantiomeric ratios are determined by HPLC analysis on a chiral stationary phase. PNP, *p*-nitrophenyl; TCP, 2,4,6-trichlorophenyl.

Model β-trifluoromethyl α,β-unsaturated *p*-nitrophenyl ester **6** and β-trifluoromethyl α,β-unsaturated 2,4,6-trichlorophenyl (TCP) ester **11** performed similarly in the reaction conditions, providing 66 and 63% yields, respectively, with complete enantioselectivity (>99:1 er) in both cases. This suggests that *p*-nitrophenoxide and 2,4,6-trichlorophenoxide are both capable of facilitating catalyst turnover to propagate the reaction. The reaction can be performed on a gram scale (3.8 mmol) to provide compound **8a** in a 60% yield and 99:1 er. To demonstrate the utility of *p*-nitrophenyl esters,^15c^ compound **8a** was derivatized *in situ* via the addition of benzylamine to produce the amide **8b**. The absolute configuration within compound **22** was unambiguously determined by single-crystal X-ray analysis to be the *S* enantiomer, with the configuration of all other examples assigned by analogy. Extension of this protocol to the use of alternative β-substituted a,β-unsaturated PNP esters gave product yields ranging from 37 to 67% with high levels of enantioselectivity (from 85:15 to >99:1 er). The enantioselectivity was complete for all β-perhalogenated examples **20**–**22** (β-C_2_F_5_, β-CF_2_Cl, and β-CF_2_Br), with lower enantioselectivity observed for β-ester **23** (98:2 er), β-ketone **24** (97:3 er), and β-CHF_2_**19** (85:15 er). Because CHF_2_ functions as a bioisostere for an alcohol,^18^ the hydrogen-bonding abilities of these three substrates may contribute to the slightly diminished enantiomeric ratios. The ethyl ester **23** constitutes the first highly enantioselective addition of malonate to unsymmetric fumaric ester and proceeds with complete regioselectivity [20:1 regioselectivity ratio (rr)]. Additionally, the labile PNP ester offers the opportunity for facile differentiation between the two ester moieties of fumaric ester. Interestingly, full regioselectivity is also observed for the aryl ketone substrate **24**. This highlights that the activated electrophilic α,β-unsaturated acyl isothiouronium intermediate can override the inherent selectivity of the parent molecule to provide exclusive Michael addition to the α,β-unsaturated PNP ester. Although promising, limitations of the methodology include the requirement of an activating β-electron-withdrawing substituent. Alternative substrates, such as β-methyl and β-phenyl α,β-unsaturated PNP esters **25** and **26** did not provide the desired Michael addition product, returning only starting material. The β,β-disubstituted fumaric ester **27** also provided no desired product, and the incorporation of a strongly withdrawing trifluoromethyl substituent in the α position for compound **28** instead of the β position was not supported.

The variability of the nucleophilic partner was then explored, commencing with the alkyl malonate series (Scheme 3). Gratifyingly, in addition to dimethyl malonate, dimethyl fluoromalonate provided the desired fluorinated tetrasubstituted carbon-containing product **37** in 82% yield and 98:2 er; however, dimethyl methylmalonate provided no desired product likely as a result of steric hindrance at the nucleophilic site. Ethyl, isopropyl, benzyl, and *tert*-butyl malonates were then examined and showed a decrease in yield correlating with increasing steric bulk within the nucleophile: ethyl (43%) **38**, isopropyl (32%) **39**, and *tert*-butyl (0%) malonates, while the 2-fluorobenzyl malonate and benzyl malonate gave the desired products **40** and **41** in 81 and 72% yields, respectively. The relatively high yields obtained when using benzyl malonates may result from π-stacking interactions with the α,β-unsaturated acyl ammonium complex. All examples **37**–**44** provided complete enantioselectivity of >99:1 er. With the performance of the reaction in MeCN and addition of catalytic diisopropylethylamine, malononitrile could be used, giving compound **42** in 48% yield with >99:1 er. Dithiomalonates are valuable substrates as a result of their ability to be converted into aldehydes and ketones more easily than their ester counterparts.^19^ Odorless *S*,*S*-bis(4-*tert*-butyl)benzyl)propanebis(thiolate) in MeCN with catalytic diisopropylethylamine gave the desired product **43** as a precipitate after 3 h in 58% yield and >99:1 er. To the best of our knowledge, these represent the first example of malononitrile or dithiomalonate addition in an enantioselective fashion to an α,β-unsaturated ester. Finally, β-ketoesters have been previously demonstrated to provide access to dihydropyrans in HyperBTM-catalyzed reactions of homoanhydrides. This reaction also proceeded smoothly with α,β-unsaturated PNP ester to provide compound **44** in 66% yield and 99:1 er. This example does not use the ability of *p*-nitrophenoxide to reform the ester, with turnover instead facilitated by the nascent enolate. In comparison to the use of a homoanhydride substrate, use of the ester starting material represents better atom economy with *p*-nitrophenol as the only byproduct and does not require an excess of the isothiouronium precursor.

**Scheme 3:**
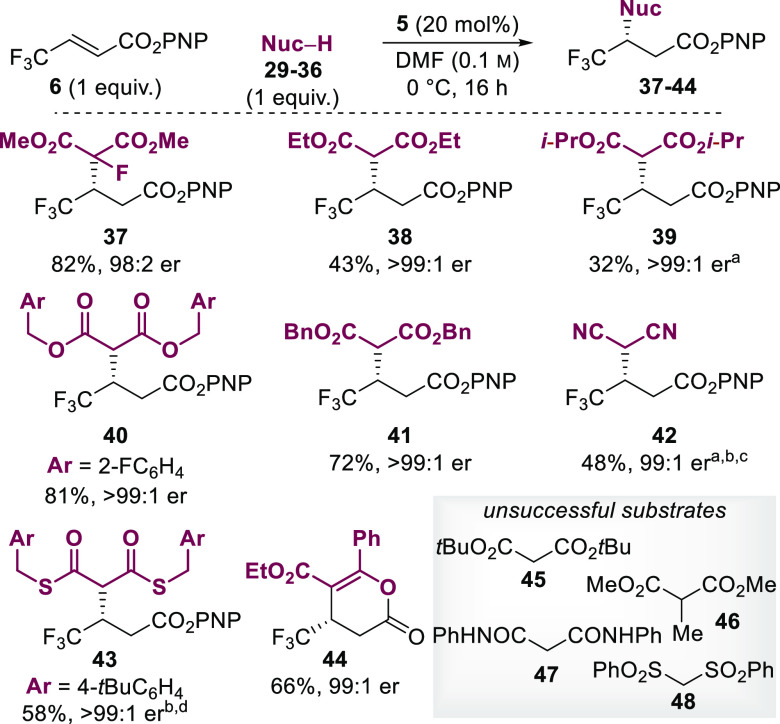
Scope and Limitations of the Addition of Nucleophiles to β-Trifluoromethyl α,β-Unsaturated PNP Ester (2*R*,3*S*)-HyperBTM was used, and the product has the opposite absolute configuration to that shown. In MeCN, and 10% diisopropylethyl amine was added. Complete in 5 h. Complete in 3 h. All yields are isolated yields after purification by column chromatography. Enantiomeric ratios are determined by HPLC analysis on a chiral stationary phase.

On the basis of prior investigations^15b^ and in combination with density functional theory (DFT) studies [M06-2X/6-31G(d,p)/IEFPCM optimized, see Supporting Information for details] based on methodology introduced by Wang et al.,^20^ the proposed catalytic cycle for the transformation is illustrated in Scheme 4. Acylation of HyperBTM **5** by the α,β-unsaturated PNP ester and displacement of *p*-nitrophenoxide were calculated to be rate-limiting (Δ*G*^⧧^ = 52.8 kJ mol^–1^), forming the corresponding α,β-unsaturated isothiouronium ion pair. This electrophilic complex is then engaged by the malonate anion in a stereoselective Michael addition through transition state **49**. Within this transition state, the isothiouronium adopts a *s*-*cis* conformation, with an stabilizing *syn*-coplanar 1,5-S···O chalcogen bond (n_o_ to σ*_S–C_)^19−23^ providing a conformational lock. To minimize 1,2 strain, the aryl stereodirecting unit adopts a pseudo-axial orientation, promoting facial selectivity in the Michael addition. This transition state is further stabilized by two weak CH···O interactions between *ortho*-C–H of the stereodirecting phenyl substituent and C–H α to positively charged nitrogen of acylated HyperBTM with the anionic malonate. Malonate addition to the electrophile is computed to be irreversible, and *anti* addition to the stereodirecting phenyl group is favored over the corresponding diastereomeric transition state by ΔΔ*G*^⧧^ = 17.5 kJ mol^–1^ (Table S1, Supporting Information). This leads to preferential formation of the (*S*)-enantiomer of the product and is consistent with the level of enantioselectivity observed experimentally (>99:1 er). Resultant isothiouronium enolate is protonated, presumably by *p*-nitrophenol, providing *p*-nitrophenoxide necessary to complete catalytic turnover^15^ and generate the Michael addition product.

**Scheme 4:**
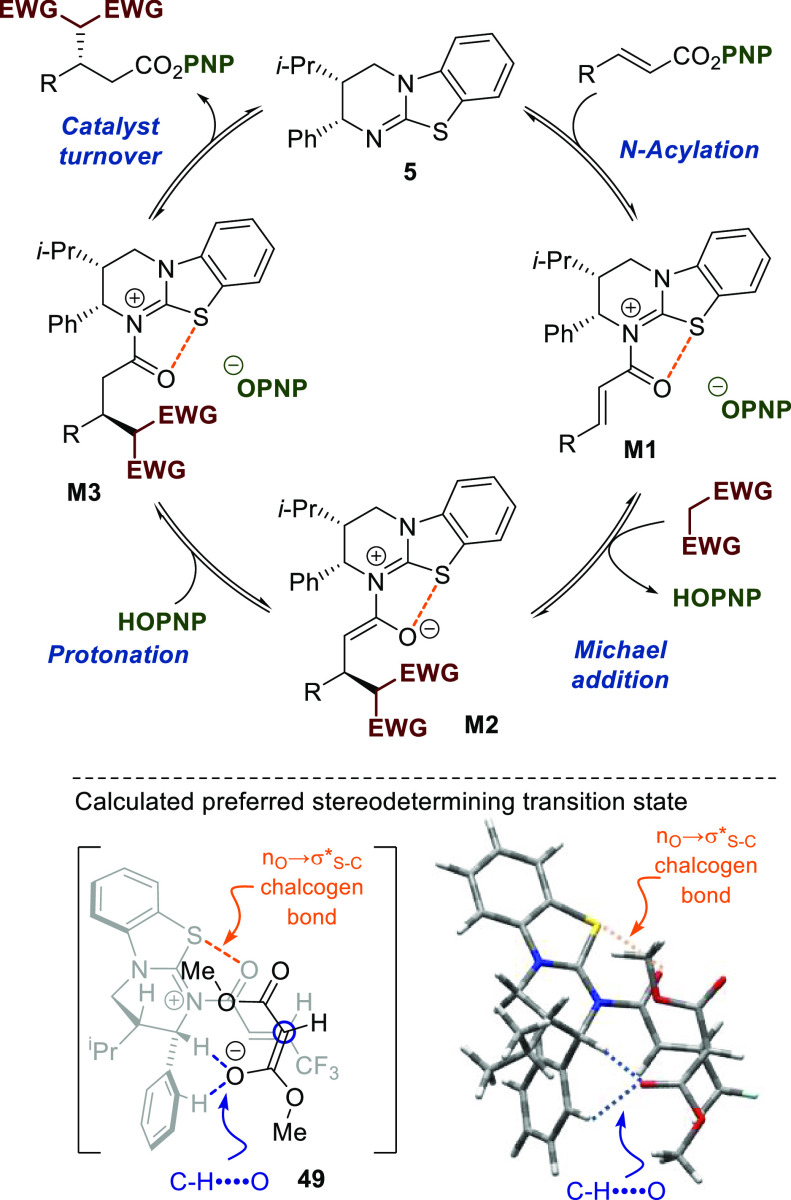
Proposed Catalytic Cycle [M06-2X/6-31G(d,p)/IEFPCM Optimized]: TS to (*S*)-Product Enantiomer

To conclude, the isothiourea-catalyzed addition of malonates and malonate derivatives to α,β-unsaturated *p*-nitrophenyl esters is disclosed. The reaction exploits the multifunctional nature of *p*-nitrophenoxide as a (1) leaving group, (2) proton shuttle, and (3) secondary nucleophile to provide catalytic turnover without the need for a pendent nucleophile within malonate. A variety of α,β-unsaturated aryl ester electrophiles containing β-electron-withdrawing substituents and malonate nucleophiles were tolerated in good yield and excellent enantioselectivity (typically >99:1 er). Exquisite regioselectivity was observed in examples with competing Michael addition reaction sites. Finally, DFT studies identified Michael addition of malonate to the chiral isothiouronium ion intermediate to be stereodetermining, consistent with experimental observations.

## Supporting Information Available

Full experimental procedures, characterization data, nuclear magnetic resonance (NMR) spectra, and HPLC chromatograms (PDF)
xyz coordinates of all optimized DFT structures in the manuscript (TXT)
The research data supporting this publication can be accessed at https://doi.org/10.17630/807cc4de-3e6c-43d6-a091-54f073849543.
