# Supplementary material for: Isothiourea-Catalyzed Enantioselective Michael Addition of Malonates to α,β-Unsaturated Aryl Esters
Source: Org Lett. 2022 Jun 2;24(22):4040–5. doi: 10.1021/acs.orglett.2c01486 (PMC9278409; doi:10.1021/acs.orglett.2c01486)
Supplement: Supplementary file 1 — ol2c01486_si_001.pdf [file ol2c01486_si_001.pdf]

Supporting Information

Isothiourea-Catalyzed Enantioselective Michael Addition of Malonates to α,β-Unsaturated Aryl Esters.

Jiufeng Wu, Claire M. Young, Amy A. Watts, Alexandra M. Z. Slawin, Gregory R. Boyce,* Michael Bühl* and Andrew D. Smith*

EaStCHEM, School of Chemistry, University of St Andrews, North Haugh, St Andrews, Fife KY16 9ST, UK
Department of Chemistry and Physics, Florida Gulf Coast University, Fort Myers, Florida 33965, United States

Email: ads10@st-andrews.ac.uk; mb105@st-andrews.ac.uk; gboyce@fgcu.edu

Table of Contents

1. General Information S1
2. General Procedures S3
3. Synthesis of Starting Materials S4
4. Synthesis of Michael Addition Products S7
5. Determination of Product Configuration by X-Ray Crystallography S17
6. Computational Details S18
7. References S25
8. Appendix I: NMR Spectra S27
9. Appendix I: HPLC Spectra S75

1. General Information

Reactions involving moisture sensitive reagents were carried out in flame-dried glassware under an argon or nitrogen atmosphere using standard vacuum line techniques and using anhydrous solvents. Anhydrous solvents (CH_2Cl_2 and toluene) were obtained from an anhydrous solvent system (purified using an alumina column, Mbraun SPS-800). All other reactions were performed in standard glassware with no precautions

to exclude air or moisture. Solvents and commercial reagents were used as supplied without further purification unless otherwise stated. (2*S*,3*R*)-HyperBTM **5** was prepared from a literature procedure.[1] The α,β-unsaturated PNP esters **6** $(CF)_3$,[2] **16** (CO_2Et),[3] **17** (PhCOR),[3] and the β-trifluoromethyl α,β-unsaturated TCP ester **11**[2] were synthesized as previously reported. All regiomeric ratios were analyzed by the ^{1}H NMR analysis of the crude reaction mixtures.

Room temperature (r.t.) refers to 20−25 °C. Temperatures of 0 °C and −78 °C were obtained using ice/water and CO_2(s)/acetone baths, respectively. Reflux conditions were obtained using a DrySyn, oil bath, or sand bath equipped with a contact thermometer.

Analytical thin layer chromatography was performed on pre-coated aluminium plates (Kieselgel 60 F_{254} silica). TLC visualisation was carried out with ultraviolet light (254 nm), followed by staining with a 1% aqueous $KMnO_4$ solution. Manual column chromatography was performed in glass columns fitted with porosity 3 sintered discs over Kieselgel 60 silica using the solvent system stated. Automated chromatography was performed on a Biotage Isolera Four running Biotage OS578 with a UV/Vis detector using the method stated and cartridges filled with Kieselgel 60 silica.

Melting points were recorded on an Electrothermal 9100 melting point apparatus and are uncorrected.

Optical rotations $[\alpha]_D^{20}$ were measured on a PerkinElmer Model 341 polarimeter operating at the sodium D line with a 100 mm path cell at 20 °C.

HPLC analyses were obtained using either a Shimadzu HPLC consisting of a DGU-20A5 degassing unit, LC-20AT liquid chromatography pump, SIL-20AHT autosampler, CMB-20A communications bus module, SPD-M20A diode array detector and a CTO-20A column oven; or a Shimadzu HPLC consisting of a DGU-20A5R degassing unit, LC-20AD liquid chromatography pump, SIL-20AHT autosampler, SPD-20A UV/Vis detector and a CTO-20A column oven. Separation was achieved using DAICEL CHIRALCEL OD-H or DAICEL CHIRALPAK AD-H or AS-H columns. All HPLC traces of enantiomerically-enriched compounds were compared with authentic racemic spectra.

^{1}H, ^{13}C, ^{19}F nuclear magnetic resonance (NMR) spectra were acquired on either a Bruker Avance 300 (^{1}H 300 MHz), Bruker Avance II 400 (^{1}H 400 MHz; ^{13}C 101 MHz; ^{19}F 376 MHz) or a Bruker Avance II 500 (^{1}H 500 MHz; ^{13}C 126 MHz) spectrometer at ambient temperature in the deuterated solvent stated. All chemical shifts are quoted in parts per million (ppm) and referenced to the residual solvent peak. All coupling constants, *J*, are quoted in Hz. Multiplicities are indicated by: s (singlet), d (doublet), t (triplet), q (quartet), and combinations thereof, and m (multiplet). The abbreviation Ar is used to denote aromatic, Ph to denote phenyl, Bn to denote benzyl, br to denote broad, and app to denote apparent.

Infrared spectra (**ν_{max}**) were recorded on a Shimadzu IRAffinity-1 Fourier transform IR spectrophotometer fitted with a Specac Quest ATR accessory (diamond puck). Spectra were recorded of either thin films or solids, with characteristic absorption wave numbers (max) reported in cm^{-1}.

High Resolution Mass spectrometry (HRMS) data were acquired by electrospray ionization time-of-flight (ESI-TOF), either at the University of St Andrews or the University of Edinburgh.

2. General Procedures

General Procedure 1: *Preparation of malonate derivatives.*

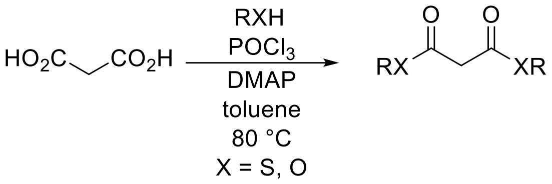

$POCl_3$ (2.2 equiv) was added dropwise to malonic acid (1.0 equiv), DMAP (0.4 equiv) in anhydrous toluene (0.25 M) at room temperature. The thiol was then added dropwise to the solution and the reaction was heated to 70 ºC for 3 h with a condenser. The reaction was then quenched by pouring into ice water. The aqueous solution was then extracted with Et_2O (3×), washed with brine, dried with $MgSO_4$, filtered, and the solvent was removed *in vacuo*. The residue was then purified by column chromatography to afford the pure product.

General Procedure 2*: Synthesis of α,β-unsaturated PNP esters*

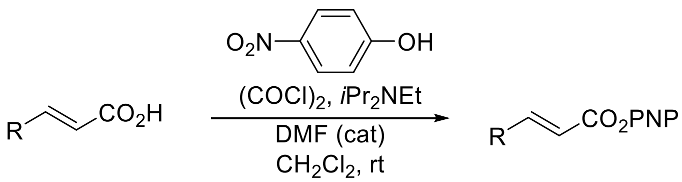

To a solution of corresponding α,β-unsaturated carboxylic acid (1.0 equiv) in anhydrous CH_2Cl_2 (0.33 M) was added oxalyl chloride (1.0 equiv) and a few drops of DMF at r.t. under a N_2 atmosphere and allowed to stir for 1h. A solution of diisopropylethylamine (2.0 equiv) and the phenol (1.0 equiv) in anhydrous CH_2Cl_2 (0.33 M) were added dropwise and the mixture was allowed to stir overnight. The solvent was then removed *in vacuo* and the residue was purified by column chromatography to afford the pure product.

General Procedure 3*: Asymmetric Michael addition of malonates to α,β-unsaturated aryl esters*

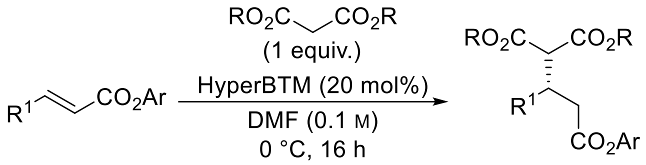

A mixture of α,β-unsaturated ester (1 equiv) and HyperBTM **5** (20 mol%) in DMF (0.1 M) was cooled to 0 ºC. The malonate (1 equiv.) was added to the pre-cooled mixture, and the resulting mixture was allowed to stir for 16 h at 0 ºC. DMF was removed under vacuum and the crude mixture was purified by column chromatography to afford the product.

3. Synthesis of Starting Materials

3.1 Synthesis of malonate derivatives.

Bis(2-fluorobenzyl) malonate (32)

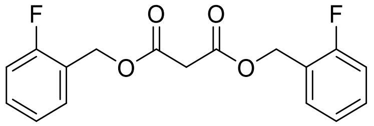

Following General Procedure 1, malonic acid (0.321 g, 3.08 mmol), DMAP (0.150 g, 1.2 mmol), 2-fluorobenzyl alcohol (0.815 g, 6.5 mmol), and phosphoryl chloride (0.63 mL, 6.8 mmol) in anhydrous toluene (20 mL) gave the title compound as a yellow oil (0.156 g, 0.59 mmol) in a 19% yield. **ν_{max}** (film), 1755 (C=O), 1732 (C=O), 1620, 1589, 1492, 1140, 999, 853; **^{1}H NMR** (500 MHz, $CDCl_3$) δ_H: 3.48 (2H, s), 5.24 (2H, s), 7.04 – 7.13 (4H, m), 7.29 – 7.38 (4H, m); **^{19}F NMR** (470 MHz, $CDCl_3$) δ_F: –117.8 (m); **^{13}C NMR** (126 MHz, $CDCl_3$) δ_C: 41.4 , 61.2 (d, *J* 4.2), 115.5 (d, *J* 21.0), 122.5 (d, *J* 14.5), 124.3 (d, *J* 3.8), 130.5 (d, *J* 8.3), 130.71 (d, *J* 3.6), 161.0, 166.1. **HRMS** (ESI^+) $C_{17}H_{15}F_2O_4$ $[M]^+$ found 321.0929, requires 321.0933 (–1.2 ppm).

***S,S*-Bis(4-*tert*-butyl)benzyl) propanebis(thiolate) (35)**

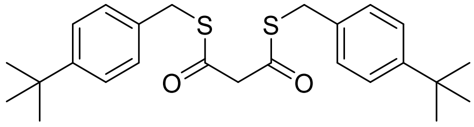

Following General Procedure 1, malonic acid (0.500 g, 4.8 mmol), DMAP (0.234 g, 1.9 mmol), 4-*tert*-butylbenzyl thiol (2.0 mL, 10.6 mmol), and phosphoryl chloride (0.98 mL, 10.6 mmol) in anhydrous toluene (20 mL) gave the title compound as a white solid (1.46 g, 3.41 mmol) in a 71% yield. **mp** 59−61 ºC; **ν_{max}** (film), 2951 (C–H), 2905 (C–H), 1703 (C=O), 1672, 1516, 1416, 995, 824; **^{1}H NMR** (500 MHz, $CDCl_3$) δ_H: 1.33 (18H, s), 3.83 (2H, s), 4.19 (4H, s), 7.24 (4H, app d, *J* 8.3), 7.35 (4H, app d, *J* 8.3); **^{13}C NMR** (126

MHz, $CDCl_3$) δ_C: 31.4, 33.8, 34.7, 57.2, 125.8, 128.7, 133.5, 150.7, 190.1. **HRMS** (ESI^+) $C_{25}H_{32}O_2S_2$ $[M]^+$ found 428.1841, requires 428.1838 (+0.7 ppm).

3.2 Synthesis of α,β-Unsaturated Aryl Esters.

4-Nitrophenyl (*E*)-4,4-difluorobut-2-enoate (12)

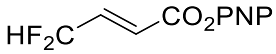

The title compound was prepared according to General Procedure 2 as a white solid. **mp** 74–76 ºC; ν_{max} (film), 1526 (N–O), 1736 (C=O); **^{1}H NMR** (400 MHz, $CDCl_3$) δ_H: 6.34 (1H, tdd, *J* 54.6, 3.9, 1.1), 6.52 (1H, dtd, *J* 15.9, 2.9, 1.0), 6.97 – 7.18 (1H, m), 7.30 – 7.52 (2H, m), 8.12 – 8.39 (2H, m); **^{19}F NMR** (376 MHz, $CDCl_3$) δ_F: –116.96 (dd, *J* 10.1, 3.2), –116.85 (dd, *J* 10.6, 3.6); **^{13}C NMR** (101 MHz, $CDCl_3$) δ_C: 112.0 (t, *J* 238.2), 122.4, 125.5, 125.8 (t, *J* 10.4), 139.6 (t, *J* 24.2), 145.7, 154.9, 162.2. **HRMS** (ESI^+) $C_{10}H_7F_2NO_4$ $[M]^+$ found 243.0344, requires 243.0338 (+2.5 ppm).

4-Nitrophenyl (*E*)-4,4,5,5,5-pentafluoropent-2-enoate (13)

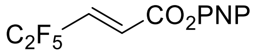

The title compound was prepared according to General Procedure 2 as a yellow oil (75% yield, ν_{max} (film), 1526 (N–O), 1755 (C=O); **^{1}H NMR** (500 MHz, $CDCl_3$) δ_H: 6.78 (1H, dt, *J* 15.8, 2.1), 7.05 (1H, dt, *J* 15.8, 11.5,), 7.34 – 7.41 (2H, m), 8.28 – 8.36 (2H, m); **^{19}F NMR** (470 Hz, $CDCl_3$) δ_F: –117.4 (q, *J* 2.3), –84.4 (t, *J* 2.2); **^{13}C NMR** (126 MHz, $CDCl_3$) δ_C: 111.4 (tq, *J* 253.0, 39.7), 118.5 (qt, *J* 285.6, 36.2), 122.3, 125.6, 129.6 (t, *J* 8.4), 133.5 (t, *J* 24.3), 145.9, 154.6, 161.3. **HRMS** (ESI^+) $C_{11}H_6F_5NO_4$ $[M]^+$ found 311.0206, requires 311.0211 (–1.6 ppm).

4-Nitrophenyl (*E*)-4-chloro-4,4-difluorobut-2-enoate (14)

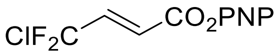

The title compound was prepared according to General Procedure 2 as a white solid.[4] **mp** 99−100 ºC; ν_{max} (film), 1522 (N–O), 1751 (C=O); **^{1}H NMR** (500 MHz, $CDCl_3$) δ_H: 6.60 (1H, dt, *J* 15.6, 1.8), 7.16 (1H, dt, *J* 15.6, 9.0), 7.35–7.38 (2H, m), 8.29–8.32 (2H, m); **^{19}F NMR** (470 MHz, $CDCl_3$) δ_F: −54.46; **^{13}C NMR** (126 MHz, $CDCl_3$) δ_C: 122.4, 123.2 (t, *J* 287.9), 124.2 (t, *J* 6.5), 125.5, 139.6 (t, *J* = 29.1 Hz), 145.8, 154.7, 161.8. **HRMS** (ESI^+) $C_{10}H_6F_2ClNO_4$ $[M]^+$ found 276.9943, requires 276.9948 (–1.8 ppm).

4-Nitrophenyl (*E*)-4-bromo-4,4-difluorobut-2-enoate (15)

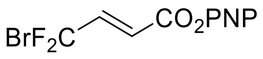

Following General Procedure 2, (*E*)-4-bromo-4,4-difluorobut-2-enoic acid (0.50 g, 2.49 mmol), oxalyl chloride (0.32 g, 3.73 mmol), diisopropylethylamine (0.87 mL, 4.98 mmol), and *p*-nitrophenol (0.35 g, 2.49 mmol) gave the title compound as a yellow solid (0.30 g, 0.93 mmol) in a 37% yield. **mp** 79−80 ºC; **ν_{max}** (film), 1522 (N–O), 1749 (C=O); **^{1}H NMR** (500 MHz, $CDCl_3$) δ_H: 6.52 (1H, dt, *J* 15.6, 1.7), 7.22 (1H, dt, *J* 15.6, 10.0), 7.36 (2H, app d, *J* 8.7), 8.31 (2H, app d, *J* 8.7); **^{19}F NMR** (376 MHz, $CDCl_3$) δ_F: –50.6; **^{13}C NMR** (126 MHz, $CDCl_3$) δ_C: 114.7 (t, *J* 301.8), 122.4, 122.6 (t, *J* 6.8), 125.6, 141.0 (t, *J* 25.9), 145.7, 154.8, 161.9. **HRMS** (ESI$^+$) $C_{10}H_6{}^{79}BrF_2NO_4$ $[M]^+$ found 320.9437, requires 320.9443 (−1.9 ppm).

4. Synthesis of Michael Addition Products.

(*S*)-1,1-Dimethyl 3-(4-nitrophenyl) 2-(trifluoromethyl)propane-1,1,3-tricarboxylate (8a)

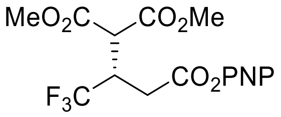

The title compound was prepared according to General Procedure 3 from (*E*)-4-nitrophenyl 4,4,4-trifluorobut-2-enoate **6** (26.0 mg, 0.1 mmol), (2*S*,3*R*)-HyperBTM **5** (6.2 mg, 0.02 mmol) and dimethylmalonate **7** (0.011 mL, 0.1 mmol) in DMF (1.0 mL) at 0 ºC for 16 h. The reaction was concentrated under reduced pressure and purified by column chromatography (5:3 CH_2Cl_2:Petrol) to give the title compound (26.1 mg, 66%) as white solid. **mp** 74−76 ºC. ν_{max} (film) / cm^{-1} 3035 (C–H), 1751 (C=O); $[\alpha]_D^{20}$ +18.3 (*c* 0.1 in $CHCl_3$); **Chiral HPLC analysis**: Chiralpak AD-H (90:10 hexane:IPA, flow rate 1.00 $mL.min^{-1}$, 211 nm, 30 °C) t_R (minor): 31.6 min, t_R (major): 19.3 min, >99:1 er; **^{1}H NMR** (500 MHz, $CDCl_3$) δ_H: 3.01 (1H, dd, *J* 17.7, 7.2), 3.23 (1H, dd, *J* 17.6, 4.8), 3.66 – 3.76 (1H, m), 3.78 (6H, s), 3.88 (1H, d, *J* 5.3), 7.27 – 7.34 (2H, m), 8.23 – 8.30 (2H, m); **^{19}F NMR** (470 MHz, $CDCl_3$) δ_F: −70.6 (d, *J* 8.9); **^{13}C {^{1}H} NMR** (126 MHz, $CDCl_3$) δ_C: 30.9 (q, *J* 2.2), 39.9 (q, *J* 27.9), 48.9 (q, *J* 2.6), 53.3 (CO_2CH_3), 53.5, 122.5, 125.4, 126.3 (q, *J* 280.2), 145.6, 155.2, 167.2, 168.5. **HRMS** (ESI^+) $C_{15}H_{14}F_3NO_8Na$ $[M+Na]^+$ found 416.0555, requires 416.0564 (−2.2 ppm).

Gram Scale

The title compound was prepared according to General Procedure 3 from (*E*)-4-nitrophenyl 4,4,4-trifluorobut-2-enoate **6** (1.00 g, 3.82 mmol), (2*S*,3*R*)-HyperBTM **5** (236.0 mg, 0.77 mmol) and dimethylmalonate **7** (0.44 mL, 3.82 mmol) in DMF (38.0 mL) at 0 ºC for 16 h. The reaction was concentrated under reduced pressure and purified by column chromatography (5:3 CH_2Cl_2:Petrol) to give the title compound (897 mg, 60%) as white solid.

Dimethyl (S)-2-(4-(benzylamino)-1,1,1-trifluoro-4-oxobutan-2-yl)malonate (8b)

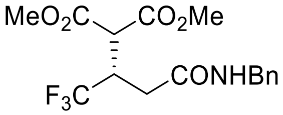

The title compound was prepared according to a modified version of General Procedure 3 from (*E*)-4-nitrophenyl 4,4,4-trifluorobut-2-enoate **6** (62.0 mg, 0.24 mmol), (2*S*,3*R*)-HyperBTM **5** (15.0 mg, 0.05 mmol) and dimethylmalonate **7** (0.027 mL, 0.24 mmol) in DMF (2.4 mL) at 0 ºC for 16 h. The reaction was then quenched with benzylamine (0.029 mL, 0.24 mmol) at 0 ºC and allowed to warm to room temperature

over 1 h. The reaction was then diluted with ether, washed with 1 M HCl, sat Na_2CO_3 (3×), brine, dried with $MgSO_4$, and filtered. The organics were then concentrated under reduced pressure and purified by column chromatography 30% EtOAc: Hexane) to give the title compound (46 mg, 54%) as white solid. **mp** 83−85 ºC. **ν_{max}** (film) / cm^{-1} 3302 (N–H), 1749 (C=O), 1742 (C=O), 1647, 1560, 1269, 1148, 978, 901; $[\alpha]_D^{20}$ +18.8 (*c* 1.0 in $CHCl_3$); **Chiral HPLC analysis**: Chiralpak AD-H (90:10 hexane:IPA, flow rate 1.00 mL.min^{-1}, 211 nm, 30 °C) t_R (minor): 16.5 min, t_R (major): 11.3 min, 99:1 er; **^{1}H NMR** (500 MHz, $CDCl_3$) δ_H: 2.61 (1H, dd, *J* 15.9, 6.7), 2.81 (1H, dd, *J* 15.8, 5.7), 3.69 – 3.77 (1H, m), 3.74 (3H, s), 3.75 (3H, s), 3.86 (1H, d, *J* 4.7), 4.34 (1 H, dd, *J* 14.8, 5.4), 4.46 (1 H, dd, *J* 14.7, 6.0), 6.20 – 6.30 (1 H, m), 7.18 – 7.38 (5 H, m).; **^{19}F NMR** (470 MHz, $CDCl_3$) δ_F: −70.1 (CF_3, d, *J* 9.3); **^{13}C {^{1}H} NMR** (126 MHz, $CDCl_3$) δ_C: 32.2 (q, *J* 2.2), 40.2 (q, *J* 27.9), 43.8, 49.1 (q, *J* 2.3), 53.0 (CO_2CH_3), 53.3 (CO_2CH_3), 126.6 (q, *J* 280.4), 127.6, 127.9, 128.8, 129.9, 138.0, 167.4, 167.6, 169.0. **HRMS** (ESI^+) $C_{16}H_{18}F_3NO_5Na$ $[M+Na]^+$ found 384.1018, requires 384.1030 (−3.1 ppm).

1,1-Dimethyl 3-(2,4,6-trichlorophenyl) (*S*)-2-(trifluoromethyl)propane-1,1,3-tricarboxylate (18)

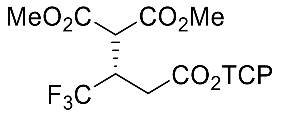

The title compound was prepared according to General Procedure 3 from 2,4,6-trichlorophenyl (*E*)-4,4,4-trifluorobut-2-enoate **11** (31.9 mg, 0.1 mmol), (2*S*,3*R*)-HyperBTM **5** (6.2 mg, 0.02 mmol) and dimethylmalonate **7** (0.011 mL, 0.1 mmol) in DMF (1.0 mL) at 0 ºC for 16 h. The reaction was concentrated under reduced pressure and purified by column chromatography (1:1 CH_2Cl_2:Petrol) to give the title compound (28.4 mg, 63%) as white solid. **mp** 67−68 ºC. **ν_{max}** (film) / cm^{-1} 3091 (C–H), 3010 (C–H), 2960 (C–H), 1776 (C=O), 1735 (C=O); $[\alpha]_D^{20}$ +15.3 (*c* 0.1 in $CHCl_3$); **Chiral HPLC analysis**: Chiralpak AD-H (90:10 hexane:IPA, flow rate 1.00 mL.min^{-1}, 211 nm, 30 °C) t_R (minor): 7.8 min, t_R (major): 6.5 min, >99:1 er; **^{1}H NMR** (500 MHz, $CDCl_3$) δ_H: 3.13 (1H, dd, *J* 18.3, 7.9), 3.33 (1H, dd, *J* 18.3, 3.5), 3.67 – 3.78 (1H, m), 3.80 (6H, s), 3.91 (1H, d, *J* 5.0), 7.38 (2H, s,); **^{19}F NMR** (376 MHz, $CDCl_3$) δ_F: −70.32; **^{13}C {^{1}H} NMR** (126 MHz, $CDCl_3$) δ_C: 30.1 (q, *J* 1.9), 39.6 (q, *J* 28.1), 49.0 (m), 53.3, 53.5, 126.23 (q, *J* 280.3), 128.8, 129.7, 132.4, 142.8, 167.1, 167.2. **HRMS** (ESI^+) $C_{15}H_{12}F_3Cl_3O_6Na$ $[M+Na]^+$ found 472.9532, requires 472.9544 (−2.5 ppm).

1,1-Dimethyl 3-(4-nitrophenyl) (*S*)-2-(difluoromethyl)propane-1,1,3-tricarboxylate (19)

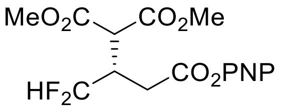

The title compound was prepared according to General Procedure 3 from (*E*)-4-nitrophenyl 4,4-difluorobut-2-enoate **12** (76.0 mg, 0.3 mmol), (2*R*,3*S*)-HyperBTM **5** (19.0 mg, 0.064 mmol) and dimethylmalonate **7** (0.036 mL, 0.3 mmol) in DMF (3.0 mL) at 0 ºC for 16 h. The reaction was concentrated under reduced pressure and purified by column chromatography (1:1 CH_2Cl_2:Petrol) to give the title compound (70.0 mg, 60%) as colourless oil. **ν_{max}** (film) / cm^{-1} 3091 (C-H), 2960 (C–H), 1776 (C=O), 1755 (C=O), 1734 (C=O); $[\alpha]_D^{20}$ +21.3 (*c* 0.1 in $CHCl_3$); **Chiral HPLC analysis**: Chiralcel OD-H (85:15 hexane:IPA, flow rate 1.00 mL.min^{-1}, 211 nm, 30 °C) t_R (minor): 18.7 min, t_R (major): 17.2 min, 85:15 er; **^{1}H NMR** (400 MHz, $CDCl_3$) δ_H: 3.00 (2H, d, *J* 6.3), 3.27 (1H, ddqd, *J* 17.8, 12.1, 6.2, 3.8), 3.78 – 3.80 (7H, m), 6.17 (1H, td, *J* 56.2, 3.8), 7.27 – 7.33 (2H, m), 8.24 – 8.31 (2H, m); **^{19}F NMR** (471 MHz, $CDCl_3$) δ_F: −124.57 (ddd, *J* 286.5, 56.3, 17.2), −121.34 (ddd, *J* 286.9, 56.1, 11.8); **^{13}C {^{1}H} NMR** (126 MHz, $CDCl_3$) δ_C: 30.4 (t, *J* 4.2), 39.3 (t, *J* 21.1), 49.4 (t, *J* 4.2), 53.2, 53.3, 115.9 (t, *J* 243.3), 122.6, 125.4, 145.6, 155.2, 167.9, 168.0, 169.2; **HRMS** (ESI^+) $C_{15}H_{15}F_2N_2O_8Na$ $[M+Na]^+$ found 398.0648, requires 398.0658 (−2.5 ppm).

(*S*)-1,1-Dimethyl 3-(4-nitrophenyl) 2-(perfluoroethyl)propane-1,1,3-tricarboxylate (20)

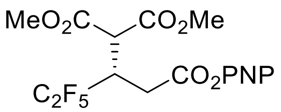

The title compound was prepared according to General Procedure 3 from (*E*)-4-nitrophenyl 4,4,5,5,5-pentafluoropent-2-enoate **13** (31.1 mg, 0.1 mmol), (2*S*,3*R*)-HyperBTM **5** (6.2 mg, 0.02 mmol) and dimethylmalonate **7** (0.011 mL, 0.1 mmol) in DMF (1.0 mL) at 0 ºC for 16 h. The reaction was concentrated under reduced pressure and purified by column chromatography (1:1 CH_2Cl_2: Petrol) to give the title compound (16.2 mg, 37%) as white solid. **mp** 86−87 ºC. **ν_{max}** (film) / cm^{-1} 2960 (C–H), 2918 (C–H), 2848 (C–H), 1739 (C=O), 1703 (C=O); $[\alpha]_D^{20}$ +17.6 (*c* 0.1 in $CHCl_3$); **Chiral HPLC analysis**: Chiralpak AD-H (90:10 hexane:IPA, flow rate 1.00 mL.min^{-1}, 211 nm, 30 °C) t_R (minor): 24.2 min, t_R (major): 11.9 min, >99:1 er; **^{1}H NMR** (500 MHz, $CDCl_3$) δ_H: 3.12 (1H, dd, *J* 18.4, 6.8), 3.33 (1H, dd, *J* 18.5, 4.4), 3.76 – 3.85 (1H, m), 3.79 (3H, s), 3.80 (3H, s), 3.97 (1H, d, *J* 4.0), 7.27 – 7.33 (2H, m), 8.24 – 8.30 (2H, m); **^{19}F NMR** (471 MHz, $CDCl_3$) δ_F: −118.23 (app dd, *J* 275.4, 16.8), −117.48 (app dd, 275.6, 14.2), −82.30 (app s); **^{13}C {^{1}H} NMR** (126 MHz, $CDCl_3$) δ_C: 30.4 (t, *J* 4.1), 37.2 (t, *J* 20.8), 48.3 (t, *J* 4.0), 53.3, 53.6, 115.2 (tq, *J*

258.2, 37.6), 119.0 (qt, *J* 286.7, 36.3), 122.5, 125.4, 145.7, 155.2, 167.4, 168.7. **HRMS** (ESI^+) $C_{16}H_{14}F_5NO_8Na$ $[M+Na]^+$ found 466.0527, requires 466.0532 (−1.1 ppm).

1,1-Dimethyl 3-(4-nitrophenyl) (*S*)-2-(chlorodifluoromethyl)propane-1,1,3-tricarboxylate (21)

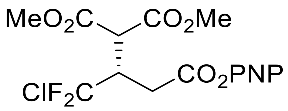

The title compound was prepared according to General Procedure 3 from (*E*)-4-nitrophenyl 4-chloro-4,4-difluorobut-2-enoate **14** (27.8 mg, 0.1 mmol), (2*S*,3*R*)-HyperBTM **5** (6.2 mg, 0.02 mmol) and dimethylmalonate **7** (0.011 mL, 0.1 mmol) in DMF (1.0 mL) at 0 ºC for 16 h. The reaction was concentrated under reduced pressure and purified by column chromatography (4:1 CH_2Cl_2: Petrol) to give the title compound (27.3 mg, 67%) as white solid. **mp** 78−80 ºC. ν_{max} (film) / cm^{-1} 3118 (C–H), 2958 (C–H), 2852 (C–H), 1759 (C=O), 1738 (C=O); $[\alpha]_D^{20}$ +14.0 (*c* 0.1 in $CHCl_3$); **Chiral HPLC analysis**: Chiralpak AD-H (90:10 hexane:IPA, flow rate 1.00 mL.min^{-1}, 211 nm, 30 °C) t_R (minor): 24.2 min, t_R (major): 11.9 min, >99:1 er. **^{1}H NMR** (500 MHz, $CDCl_3$) δ_H: 3.07 (1H, dd, *J* 17.7, 6.5), 3.29 (1H, dd, *J* 17.7, 5.1), 3.79 (6H, s), 3.82 – 3.93 (1H, m), 3.99 (1H, d, *J* 4.9), 7.29 – 7.35 (2H, m), 8.25 – 8.31 (2H, m); **^{19}F NMR** (470 MHz, $CDCl_3$) δ_F: −55.43 (dd, *J* 167.6, 4.0), −55.18 (dd, *J* 167.6, 4.0); **^{13}C {^{1}H} NMR** (126 MHz, $CDCl_3$) δ_C: 32.1 (t, *J* 2.3), 46.2 (t, *J* 23.7), 50.0 (t, *J* 2.0), 53.3, 53.6, 122.6, 125.4, 130.0 (t, *J* 295.5), 145.7, 155.3, 167.3, 167.4, 168.6. **HRMS** (ESI^+) $C_{15}H_{14}ClF_2NO_8Na$ $[M+Na]^+$ found 432.0259, requires 432.0268 (−2.1 ppm).

1,1-Dimethyl 3-(4-nitrophenyl) (*S*)-2-(bromodifluoromethyl)propane-1,1,3-tricarboxylate (22)

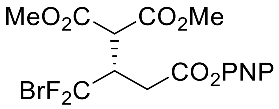

The title compound was prepared according to General Procedure 3 from (*E*)-4-nitrophenyl 4-bromo-4,4-difluorobut-2-enoate **15** (32.2 mg, 0.1 mmol), (2*S*,3*R*)-HyperBTM **5** (6.2 mg, 0.02 mmol) and dimethylmalonate **7** (0.011 mL, 0.1 mmol) in DMF (1.0 mL) at 0 ºC for 16 h. The reaction was concentrated under reduced pressure and purified by column chromatography (4:1 CH_2Cl_2:Petrol) to give the title compound (27.9 mg, 61%) as white solid. **mp** 82−83 ºC. ν_{max} (film) / cm^{-1} 3082 (C–H), 2956 (C–H), 2850 (C–H), 1762 (C=O), 1737 (C=O); $[\alpha]_D^{20}$ +3.0 (*c* 0.1 in $CHCl_3$); **Chiral HPLC analysis**: Chiralpak AD-H (90:10 hexane:IPA, flow rate 1.00 mL.min^{-1}, 211 nm, 30 °C) t_R (minor): 30.9 min, t_R (major): 23.8 min, >99:1 er; **^{1}H NMR** (400 MHz, $CDCl_3$) δ_H: 3.07 (1H, dd, *J* 17.8, 6.3), 3.27 (1H, dd, *J* 17.8, 5.2), 3.79 (6H, s), 3.86 (1H, tq, *J* 11.1, 5.4), 4.00 (1H, d, *J* 5.0), 7.29 – 7.37 (2H, m), 8.24 – 8.32 (2H, m); **^{19}F NMR** (471 MHz, $CDCl_3$) δ_F: −48.84 (t, *J* 11.0); **^{13}C {^{1}H} NMR** (101 MHz, $CDCl_3$) δ_C: 32.6 (t, *J* 2.5), 47.8 (t, *J* 21.3),

50.4 (t, *J* 2.3), 53.3, 53.6, 122.6, 123.4 (t, *J* 308.6), 125.4, 145.6, 155.3, 167.3, 168.5. **HRMS** (ESI$^+$) $C_{15}H_{14}BrF_2NO_8Na$ $[M+Na]^+$ found 475.9754, requires 475.9763 (−1.9 ppm).

2-Ethyl 1,1-dimethyl 3-(4-nitrophenyl) (*S*)-propane-1,1,2,3-tetracarboxylate (23)

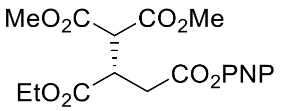

The title compound was prepared according to General Procedure 3 from ethyl (4-nitrophenyl) fumarate **16** (53.0 mg, 0.2 mmol), (2*S*,3*R*)-HyperBTM **5** (12.4 mg, 0.02 mmol) and dimethylmalonate **7** (0.022 mL, 0.2 mmol) in DMF (2.0 mL) at 0 ºC for 16 h. The reaction was concentrated under reduced pressure and purified by column chromatography (100% CH_2Cl_2) to give the title compound (51.3 mg, 65%) as colorless oil. $\mathbf{\nu_{max}}$ (film) / cm^{-1} 2968 (C–H), 1765 (C=O), 1749 (C=O); $[\boldsymbol{\alpha}]_{\boldsymbol{D}}^{\mathbf{20}}$ −19.3 (*c* 0.1 in $CHCl_3$); **Chiral HPLC analysis**: Chiralpak AD-H (90:10 hexane:IPA, flow rate 1.00 mL.min^{-1}, 211 nm, 30 °C) t_R (minor): 53.8 min, t_R (major): 41.5 min, 98:2 er; **^{1}H NMR** (400 MHz, $CDCl_3$) δ_H: 1.28 (3H, t, *J* 7.1), 3.00 (1H, dd, *J* 17.1, 4.8), 3.14 (1H, dd, *J* 17.1, 8.2), 3.67 (1H, ddd, *J* 8.2, 6.4, 4.8), 3.77 (3H, s), 3.78 (3H, s), 4.03 (1H, d, *J* 6.4), 4.16 – 4.24 (2H, m), 7.27 – 7.33 (2H, m), 8.23 – 8.30 (2H, m); **^{13}C {^{1}H} NMR** (101 MHz, $CDCl_3$) δ_C: 14.1, 33.5, 40.6, 51.8, 53.1, 62.0, 122.5, 125.3, 145.6, 155.4, 168.0, 168.3, 169.4, 171.3; **HRMS** (ESI$^+$) $C_{17}H_{19}NO_{10}Na$ $[M+Na]^+$ found 420.0897, requires 420.0901 (−1.0 ppm).

1,1-Dimethyl 3-(4-nitrophenyl) (*S*)-2-benzoylpropane-1,1,3-tricarboxylate (24)

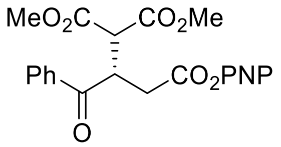

The title compound was prepared according to General Procedure 3 from (*E*)-4-nitrophenyl 4-oxo-4-phenylbut-2-enoate **17** (59.4 mg, 0.2 mmol), (2*S*,3*R*)-HyperBTM **5** (12.4 mg, 0.04 mmol) and dimethylmalonate **7** (0.022 mL, 0.2 mmol) in DMF (2.0 mL) at 0 ºC for 16 h. The reaction was concentrated under reduced pressure and purified by column chromatography (1:2 EtOAc:Petrol) to give the title compound (42.1 mg, 49%) as orange oil. $\mathbf{\nu_{max}}$ (film) / cm^{-1} 3086 (C-H), 2956 (C–H), 1751 (C=O), 1732 (C=O), 1683 (C=O); $[\boldsymbol{\alpha}]_{\boldsymbol{D}}^{\mathbf{20}}$ −29.6 (*c* 0.1 in $CHCl_3$); **Chiral HPLC analysis**: Chiralpak AS-H (95:5 hexane:IPA, flow rate 1.00 mL.min^{-1}, 211 nm, 40 °C) t_R (minor): 24.7 min, t_R (major): 27.5 min, 97:3 er; **^{1}H NMR** (500 MHz, $CDCl_3$) δ_H: 3.03 (1H, dd, *J* 16.9, 5.8), 3.09 (1H, dd, *J* 16.9, 7.0), 3.64 (3H, s), 3.76 (3H, s), 4.04 (1H, d, *J* 8.8), 4.69 (1H, ddd, *J* 8.9, 7.0, 5.8), 7.15 – 7.19 (2H, m), 7.47 – 7.53 (2H, m), 7.57 – 7.63 (1H, m), 8.00 – 8.04 (2H, m), 8.19 – 8.23 (2H, m); **^{13}C {^{1}H} NMR** (126 MHz, $CDCl_3$) δ_C: 34.6, 41.8,

52.8, 53.1, 53.2, 122.5, 125.3, 128.8, 129.1, 133.9, 135.5, 145.5, 155.1, 168.2, 168.4, 169.1, 199.3 (*C*(O)Ph). **HRMS** (ESI^+) $C_{21}H_{20}NO_9$ $[M+H]^+$ found 430.1127, requires 430.1132 (−1.2 ppm).

1,1-Dimethyl 3-(4-nitrophenyl) (*S*)-1-fluoro-2-(trifluoromethyl)propane-1,1,3-tricarboxylate (37)

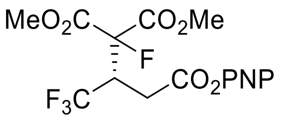

The title compound was prepared according to General Procedure 3 from (*E*)-4-nitrophenyl 4,4,4-trifluorobut-2-enoate **6** (52.2 mg, 0.2 mmol), (2*S*,3*R*)-HyperBTM **5** (12.4 mg, 0.04 mmol) and dimethyl 2-fluoromalonate **29** (30.0 mg, 0.2 mmol) in DMF (2.0 mL) at 0 ºC for 16 h. The reaction was concentrated under reduced pressure and purified by column chromatography (4:1 CH_2Cl_2:Petrol) to give the title compound (67.4 mg, 82%) as white solid. **mp** 54−56 ºC. ν_{max} (film) / cm^{-1} 2968 (C–H), 1768 (C=O), 1749 (C=O); $[\alpha]_D^{20}$ +36.0 (*c* 0.1 in $CHCl_3$); **Chiral HPLC analysis**: Chiralcel OD-H (90:10 hexane:IPA, flow rate 1.00 mL.min^{-1}, 211 nm, 30 °C) t_R (minor): 22.8 min, t_R (major): 15.6 min, 98:2 er; **^{1}H NMR** (400 MHz, $CDCl_3$) δ_H: 3.01 (2H, d, *J* 5.9), 3.87 (3H, s), 3.90 (3H, s), 4.03 – 4.19 (1H, m), 7.29 – 7.33 (2H, m), 8.25 – 8.29 (2H, m); **^{19}F NMR** (376 MHz, $CDCl_3$) δ_F: −173.85 (qd, *J* 9.8, 2.7), −66.88 (d, *J* 9.9); **^{13}C {^{1}H} NMR** (126 MHz, $CDCl_3$) δ_C: 30.2 (t, *J* 2.5), 44.4 (qd, *J* 27.8, 20.0), 54.3, 54.5, 92.5 (d, *J* 210.5), 122.5, 125.1 (q, *J* 281.4), 125.5, 145.8, 155.0, 164.2 (d, *J* 25.9), 164.5 (d, *J* 24.4), 167.7. **HRMS** (ESI^+) $C_{15}H_{13}F_4NO_8Na$ $[M+Na]^+$ found 434.0459, requires 434.0470 (−2.5 ppm).

1,1-Diethyl 3-(4-nitrophenyl) (*S*)-2-(trifluoromethyl)propane-1,1,3-tricarboxylate (38)

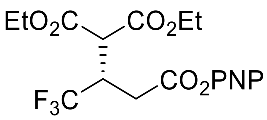

The title compound was prepared according to General Procedure 3 from (*E*)-4-nitrophenyl 4,4,4-trifluorobut-2-enoate **6** (52.2 mg, 0.2 mmol), (2*S*,3*R*)-HyperBTM **5** (12.4 mg, 0.02 mmol) and diethylmalonate **30** (0.030 mL, 0.2 mmol) in DMF (2.0 mL) at 0 ºC for 16 h. The reaction was concentrated under reduced pressure and purified by column chromatography (2:1 CH_2Cl_2:Petrol) to give the title compound (36.2 mg, 43%) as colourless oil. ν_{max} (film) / cm^{-1} 3088 (C–H), 2985 (C–H), 2899 (C–H), 1732 (C=O); $[\alpha]_D^{20}$ +33.6 (*c* 0.1 in $CHCl_3$); **Chiral HPLC analysis**: Chiralpak AD-H (90:10 hexane:IPA, flow rate 1.00 mL.min^{-1}, 211 nm, 30 °C) t_R (minor): 24.8 min, t_R (major): 15.4 min, >99:1 er; **^{1}H NMR** (500 MHz, $CDCl_3$) δ_H: 1.29 (6H, q, *J* 7.0), 3.01 (1H, dd, *J* 17.6, 7.4), 3.24 (1H, dd, *J* 17.6, 4.5), 3.66–3.77 (1H, m), 3.84 (1H, d, *J* 5.2), 4.25 (4H, qd, *J* 7.1, 1.6), 7.28 – 7.34 (2H, m), 8.24 – 8.31 (2H, m); **^{19}F NMR** (471 MHz, $CDCl_3$) δ_F: −70.4 (d, *J* 8.7); **^{13}C {^{1}H} NMR** (126 MHz, $CDCl_3$) δ_C: 14.0, 31.0 (m), 39.9 (q, *J* 27.8),

49.4 (m), 62.4, 62.7, 122.5, 125.3, 126.4 (q, *J* 280.2), 145.6, 155.3, 166.8, 168.5. **HRMS** (ESI$^+$) $C_{17}H_{18}F_3NO_8Na$ [M+Na]$^+$ found 444.0869, requires 444.0877 (−1.7 ppm).

1,1-Diisopropyl 3-(4-nitrophenyl) (*S*)-2-(trifluoromethyl)propane-1,1,3-tricarboxylate (39)

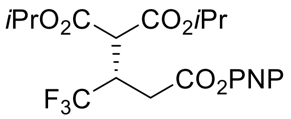

The title compound was prepared according to General Procedure 3 from (*E*)-4-nitrophenyl 4,4,4-trifluorobut-2-enoate **6** (100.0 mg, 0.4 mmol), (2*R*,3*S*)-HyperBTM **5** (23.5 mg, 0.08 mmol) and diisopropylmalonate **31** (0.072 mL, 0.4 mmol) in DMF (4.0 mL) at 0 ºC for 16 h. The reaction was concentrated under reduced pressure and purified by column chromatography (100% CH_2Cl_2) to give the title compound (54.2 mg, 32%) as colorless oil. **ν$_{max}$** (neat) / cm^{-1} 2984 (C–H), 1768 (C=O), 1730 (C=O), 1346, 1097, 910, 862; $[\alpha]_D^{20}$ –18.8 (*c* 1.5 in $CHCl_3$); **Chiral HPLC analysis**: Chiralcel OD-H (99:1 hexane:IPA, flow rate 1.00 mL.min^{-1}, 211 nm, 30 °C) t_R (minor): 18.6 min, t_R (major): 15.1 min, >99:1 er; **^{1}H NMR** (500 MHz, $CDCl_3$) δ_H: 1.23 – 1.30 (12H, m), 3.00 (1H, dd, *J* 17.6, 7.6), 3.22 (1H, dd, *J* 17.6, 4.2), 3.65 – 3.75 (1H, m), 3.77 (1H, d, *J* 5.3), 5.06 – 5.12 (2H, m), 7.27 – 7.34 (2H, m), 8.24 – 8.31 (2H, m); **^{19}F NMR** (376 MHz, $CDCl_3$) δ_F: −70.26; **^{13}C {^{1}H} NMR** (126 MHz, $CDCl_3$) δ_C: 21.6, 31.1 (m), 39.8 (q, *J* 27.7), 49.8 – 49.9 (m), 70.2, 70.6, 122.6, 126.5 (q, *J* 280.1), 125.4, 145.6, 155.3, 166.3, 166.4, 168.6. **HRMS** (ESI$^+$) $C_{19}H_{22}F_3NO_8Na$ [M+Na]$^+$ found 472.1197, requires 472.1190 (−1.4 ppm).

1,1-Bis(2-fluorobenzyl) 3-(4-nitrophenyl) (*S*)-2-(trifluoromethyl)propane-1,1,3-tricarboxylate (40)

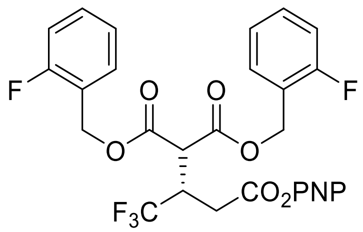

The title compound was prepared according to General Procedure 3 from (*E*)-4-nitrophenyl 4,4,4-trifluorobut-2-enoate **6** (130.5 mg, 0.5 mmol), (2*S*,3*R*)-HyperBTM **5** (31.0 mg, 0.1 mmol) and bis(2-fluorobenzyl) malonate **32** (160.5 mg, 0.5 mmol) in DMF (5.0 mL) at 0 ºC for 16 h. The reaction was concentrated under reduced pressure and purified by column chromatography (1:4 EtOAc:Petrol) to give the title compound (235.7 mg, 81%) as colourless oil. **ν$_{max}$** (film) / cm^{-1} 3078 (C–H), 2951 (C–H), 1759 (C=O), 1737 (C=O); $[\alpha]_D^{20}$+14.0 (*c* 0.1 in $CHCl_3$); **Chiral HPLC analysis**: Chiralcel OD-H (90:10 hexane:IPA, flow rate 1.00 mL.min^{-1}, 211 nm, 30 °C) t_R (minor): 35.1 min, t_R (major): 24.0 min, 99:1 er; **^{1}H NMR** (500 MHz, $CDCl_3$) δ_H: 3.02 (1H, dd, *J* 17.7, 7.4), 3.22 (1H, dd, *J* 17.7, 4.6), 3.75 (1H, m), 3.97

(1H, d, *J* 5.1), 5.20 – 5.30 (4H, m), 7.02 – 7.15 (4H, m), 7.25 – 7.38 (6H, m), 8.23 – 8.30 (2H, m); **^{19}F NMR** (471 MHz, $CDCl_3$) δ_F: −117.8 (dt, *J* 11.7, 6.4), −117.7 (dt, *J* 11.8, 6.2), −70.4 (d, *J* 8.7); **^{13}C {^{1}H} NMR** (126 MHz, $CDCl_3$) δ_C: 30.9, 39.9 (q, *J* 27.9), 49.2, 62.1 (d, *J* 4.4), 62.4 (d, *J* 4.2), 115.6 (d, *J* 4.5), 115.8 (d, *J* 4.4), 121.8 (d, *J* 5.5), 121.9 (d, *J* 5.5), 122.5, 124.4 (app t, *J* 3.7), 125.4, 126.3 (q, *J* 280.4) 130.8 – 131.0 (m) 145.6, 155.2, 161.10 (dd, *J* 249.1, 2.5), 166.3, 166.4, 168.4; **HRMS** (ESI$^+$) $C_{27}H_{20}F_5NO_8Na$ $[M+Na]^+$ found 604.0993, requires 604.1001 (−1.3 ppm).

1,1-Dibenzyl 3-(4-nitrophenyl) (*S*)-2-(trifluoromethyl)propane-1,1,3-tricarboxylate (41)

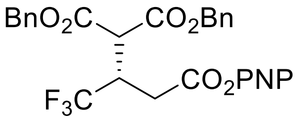

The title compound was prepared according to General Procedure 3 from (*E*)-4-nitrophenyl 4,4,4-trifluorobut-2-enoate **6** (52.2 mg, 0.2 mmol), (2*S*,3*R*)-HyperBTM **5** (12.4 mg, 0.04 mmol) and dibenzyl malonate **33** (0.022 mL, 0.2 mmol) in DMF (2.0 mL) at 0 ºC for 16 h. The reaction was concentrated under reduced pressure and purified by column chromatography (1:4 EtOAc:Petrol) to give the title compound (78.5 mg, 72%) as white solid. **mp** 60−62 ºC. **ν_{max}** (film) / cm^{-1} 3086 (C–H), 3064 (C–H), 3035 (C–H), 2956 (C–H), 1759 (C=O), 1734 (C=O); $[\alpha]_D^{20}$ +22.6 (*c* 0.1 in $CHCl_3$); **Chiral HPLC analysis**: Chiralpak AD-H (90:10 hexane:IPA, flow rate 1.00 mL.min^{-1}, 211 nm, 30 °C) t_R (minor): 30.3 min, t_R (major): 34.3 min, >99:1 er; **^{1}H NMR** (500 MHz, $CDCl_3$) δ_H: 3.01 (1H, dd, *J* 17.7, 7.4), 3.21 (1H, dd, *J* 17.6, 4.6), 3.70 – 3.82 (1H, m), 3.97 (1H, d, *J* 5.1), 5.09 – 5.30 (4H, m), 7.19 – 7.41 (12H, m), 8.21 – 8.32 (2H, m); **^{19}F NMR** (376 MHz, $CDCl_3$) δ_F: −70.3; **^{13}C {^{1}H} NMR** (126 MHz, $CDCl_3$) δ_C: 30.9 (m), 39.9 (q, *J* 28.0), 49.4 (m), 68.2, 68.4, 122.5, 125.4, 126.4 (q, *J* 280.3), 128.5 – 128.8, 134.7, 145.6, 155.2, 166.5, 166.6, 168.4. **HRMS** (ESI$^+$) $C_{27}H_{22}F_3NO_8Na$ $[M+Na]^+$ found 568.1181, requires 568.1190 (−1.6 ppm).

4-Nitrophenyl (*S*)-3-(dicyanomethyl)-4,4,4-trifluorobutanoate (42)

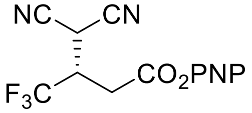

The title compound was prepared according to General Procedure 3 from (*E*)-4-nitrophenyl 4,4,4-trifluorobut-2-enoate **6** (79.0 mg, 0.3 mmol), (2*R*,3*S*)-HyperBTM **5** (18.5 mg, 0.06 mmol) and malononitrile **34** (20.0 mg, 0.3 mmol) diisopropylethylamine (0.005 mL, 0.03 mmol) in MeCN (3.0 mL) at 0 ºC for 5 h. The reaction was concentrated under reduced pressure and purified by column chromatography (4:1 CH_2Cl_2:Petrol) to give the title compound (47 mg, 48%) as colourless oil. **ν_{max}** (film) / cm^{-1} 2968 (C–H), 2899 (C–H), 1749 (C=O), 1220 (C≡N); $[\alpha]_D^{20}$ +37.2 (*c* 0.06 in $CHCl_3$); **Chiral HPLC analysis**: Chiralpak

AD-H (95:5 hexane:IPA, flow rate 1.00 mL.min^{-1}, 211 nm, 30 °C) t_R (minor): 29.4 min, t_R (major): 89.5 min, >99:1 er. **^{1}H NMR** (500 MHz, $CDCl_3$) δ_H: 3.15 (1H, dd, *J* 18.0, 7.7), 3.30 (1H, dd, *J* 18.0, 5.1), 3.51 – 3.60 (1H, m), 4.46 (1H, d, *J* 4.6), 7.30 – 7.38 (2H, m), 8.28 – 8.36 (2H, m); **^{19}F NMR** (471 MHz, $CDCl_3$) δ_F: −69.36 (d, *J* 7.6); **^{13}C {^{1}H} NMR** (126 MHz, $CDCl_3$) δ_C: 22.3 (q, *J* 3.0), 30.9 (m), 41.3 (q, *J* 29.4), 109.4, 109.7, 122.3, 124.3 (q, *J* 281.3), 125.6, 146.1, 154.4, 166.8. **HRMS** (ESI$^+$) $C_{13}H_8F_3N_3O_4Na$ [M+Na]$^+$ found 350.0355, requires 350.0360 (−1.4 ppm).

4-Nitrophenyl (*S*)-5-((4-(*tert*-butyl)benzyl)thio)-4-(((4-(*tert*-butyl)benzyl)thio)carbonyl)-5-oxo-3-(trifluoromethyl)pentanoate (43)

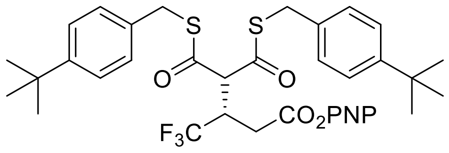

The title compound was prepared according to General Procedure 3 from (*E*)-4-nitrophenyl 4,4,4-trifluorobut-2-enoate **6** (200.0 mg, 0.77 mmol), (2*S*,3*R*)-HyperBTM **5** (47.0 mg, 0.15 mmol) and *S*,*S*-bis(4-(tert-butyl)benzyl) propanebis(thioate) **35** (328.0 mg, 0.77 mmol) diisopropylethylamine (0.013 mL, 0.08 mmol) in MeCN (7.0 mL) at 0 ºC for 3 h. The crude reaction mixture was concentrated under reduced pressure and recrystallized from MeCN to give the title compound (398 mg, 58%) as white solid. **mp** 107−108 ºC. **ν_{max}** (film) / cm^{-1} 2958 (C–H), 1770 (C=O), 1681 (C=O), 1346, 1201, 1161, 983; $[\alpha]_D^{20}$ +54.7 (*c* 1.0 in $CHCl_3$); **Chiral HPLC analysis**: Chiralpak AD-H (90:10 hexane:IPA, flow rate 1.00 mL.min^{-1}, 211 nm, 30 °C) t_R (minor): 24.1 min, t_R (major): 12.9 min, >99:1 er; **^{1}H NMR** (400 MHz, $CDCl_3$) δ_H: 1.30 (18H), 2.94 (1H, dd, *J* 17.5, 7.2), 3.22 (1H, dd, *J* 17.5, 4.3), 3.79 – 3.94 (1H, m), 4.11 – 4.32 (5H, m), 7.18 – 7.21 (4H, m), 7.29 – 7.31 (6H, m), 8.25 – 8.31 (2H, m); **^{19}F NMR** (376 MHz, $CDCl_3$) δ_F: –69.08; **^{13}C {^{1}H} NMR** (126 MHz, $CDCl_3$) δ_C: 31.1 (m), 31.4, 34.2, 34.3, 34.7, 41.0 (q, *J* 27.6), 122.6, 125.4, 125.9, 126.2 (q, 280.7), 128.7, 132.6, 145.6, 151.0, 155.2, 168.2, 190.6, 190.9. **HRMS** (ESI$^+$) $C_{35}H_{37}F_3NO_6S_2$ [M–H]$^-$ found 688.2020, requires 688.2020 (0.0 ppm).

Ethyl (*S*)-2-oxo-6-phenyl-4-(trifluoromethyl)-3,4-dihydro-2*H*-pyran-5-carboxylate (44)

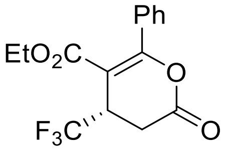

The title compound was prepared according to General Procedure 3 from (*E*)-4-nitrophenyl 4,4,4-trifluorobut-2-enoate **6** (100 mg, 0.4 mmol), (2*S*,3*R*)-HyperBTM **5** (23.5 mg, 0.08 mmol) and ethyl

benzoylacetate **36** (0.07 mL, 0.4 mmol) in DMF (3.5 mL) at 0 ºC for 16 h. The reaction was concentrated under reduced pressure and purified by column chromatography (1:1 CH_2Cl_2:Hexane) to give the title compound (80 mg, 66%) as a white solid. **mp** 66–67 ºC. **ν_{max}** (film) / cm^{-1} 2981 (C–H), 1788 (C=O), 1701 (C=O), 1225, 1153, 1039, 995; $[\alpha]_D^{20}$ +5.0 (*c* 1.0 in $CHCl_3$); **Chiral HPLC analysis**: Chiralpak AD-H (90:10 hexane:IPA, flow rate 1.00 mL.min^{-1}, 211 nm, 30 °C) t_R (minor): 8.4 min, t_R (major): 7.7 min, >99:1 er; **^{1}H NMR** (400 MHz, $CDCl_3$) δ_H: 0.97 (3H, t, *J* 7.1), 2.91 (1H, dd, *J* 17.1, 7.9), 3.11 (1H, dd, *J* 17.1, 1.4), 3.94 – 4.10 (3H, m), 7.33 – 7.55 (5H, m); **^{19}F NMR** (376 MHz, $CDCl_3$) δ_F: –72.4; **^{13}C {^{1}H} NMR** (126 MHz, $CDCl_3$) δ_C: 13.6, 27.6 (q, *J* 2.3), 37.9 (q, *J* 29.9), 61.7, 103.1, 125.8 (q, *J* 281.4), 128.2, 128.7, 130.8, 132.6, 162.2, 163.7, 165.6. **HRMS** (ESI$^+$) $C_{15}H_{14}F_3O_4Na$ $[M+Na]^+$ found 337.0657, requires 337.0658 (−0.3 ppm).

5. Determination of Product Configuration by X-Ray Crystallography

X-ray diffraction data were collected at 148 K using a Rigaku XtaLAB P200 diffractometer [Cu Kα radiation (λ = 1.54184 Å)]. Data were collected using CrystalClear[5] and processed (including correction for Lorentz, polarization and absorption) using CrysAlisPro.[6] Structures were solved by dual-space (SHELXT[7]), direct (SIR2011[8]) or charge-flipping (Superflip[9]) methods and refined by full-matrix least-squares against F_2 (SHELXL-2018/3[10]). Non-hydrogen atoms were refined anisotropically, and all hydrogen atoms were refined using a riding model. All calculations were performed using the CrystalStructure[11] interface. Crystals suitable for X-ray diffraction analysis were obtained using the vapor diffusion technique with ethyl acetate and petroleum ether at room temperature. ORTEP plot of the crystal structure of **22**, and thermal ellipsoid is set at 50% probability.

CCDC compound number	2145495
6empirical formula	$C_{15}H_{14}BrF_2NO_8$
fw	454.18
crystal description	colourless plate
crystal size [mm]	0.10×0.04×0.02
space group	C_2
a [Å]	15.4843(6)
b [Å]	8.0244(3)
c [Å]	14.6382(6)
vol [Å]3	1755.12(12)
α [°]	
β [°]	105.210(4)
γ [°]	
Z	4
ρ (calc) [g/cm3]	1.719
μ [mm−1]	3.819
F(000)	912.0
reflections collected	9017
independent reflections (R_{int})	3392 (0.0247)
data/parameters	3392/244
GOF on F2	1.09
R1 [I > 2σ(I)]	0.0409
wR2 (all data)	0.1160
largest diff. peak/hole [e/Å3]	0.43, −0.95
Flack parameter	0.09(3)

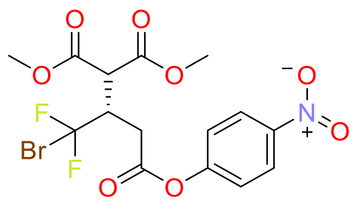

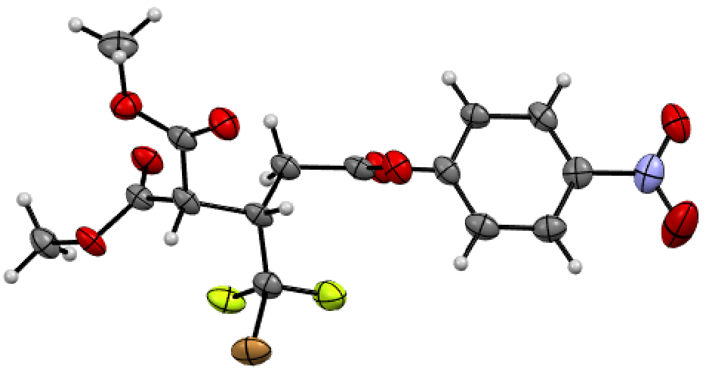

6. Computational Details

6.1 Methodology

All calculations were performed in Gaussian 09 suite of programs.[12] Where applicable, the initial geometries for the calculations were adapted from the optimised geometries in the study from Wang et al.[13] Optimisation were performed at the M06-2X[14]/6-31G(d,p)/$IEFPCM_{THF}$ level, using a polarisable continuum model in the integrated equation formalism (IEFPCM)[15] together with the parameters for THF, followed by evaluation of the harmonic vibrational frequencies at the same level. Structures were characterised as either true energy minima (all real frequencies) or a first order saddle point i.e. a transition state (a single imaginary frequency). Visual inspection of the single imaginary vibrational modes ensured the desired transition states had been found. Energies were refined by single point energy calculations at the M06-2X-D3[16]/6-311++G(2df, 2dp)//$IEFPCM_{THF}$ level. Subsequently, the potential energies from this level were combined with the thermal corrections at the M06-2X/6-31G(d,p)//$IEFPCM_{THF}$ level to give Gibbs free energies and enthalpies at 298.15 K and 1 atm. The reaction profile was calculated at 273.15 K as follows:

$$\Delta G^{273} = \Delta H^{298} - (273.15\ K)\Delta S^{298} \qquad \text{(eq 1)}$$

The enantiomeric ratio (*er*) was calculated from a Boltzmann equilibrium at that temperature.

6.2 Results and Discussion

Our target system bears considerable resemblance to that from Wang et al.[13] for the synthesis of pyridones and pyranones. Both systems are catalysed by isothiourea HyperBTM (**5**) and include Michael addition reactions to fluorinated α,β-unsaturated esters. The key differences are that we use a different aryl alcohol ester (*para*-nitrophenolate, OPNP, instead of 2,4,6-trichlorophenolate) and a different nucleophile for Michael addition (dimethyl malonate ester **7** instead of 2-acylbenzazole). In the system of Wang et al.,[13] catalytic turnover is achieved from an intramolecular cyclisation step, whereas in our system, catalytic turnover is driven by the free aryl oxide. Because of this close similarity of both systems, it is reasonable to assume that they would have similar transition state and intermediate geometries in the initial steps. We therefore used the study from Wang el al. as inspiration for our own calculations, which were performed at the same level of theory. The resulting profile including the key steps is summarised in Figure S1.

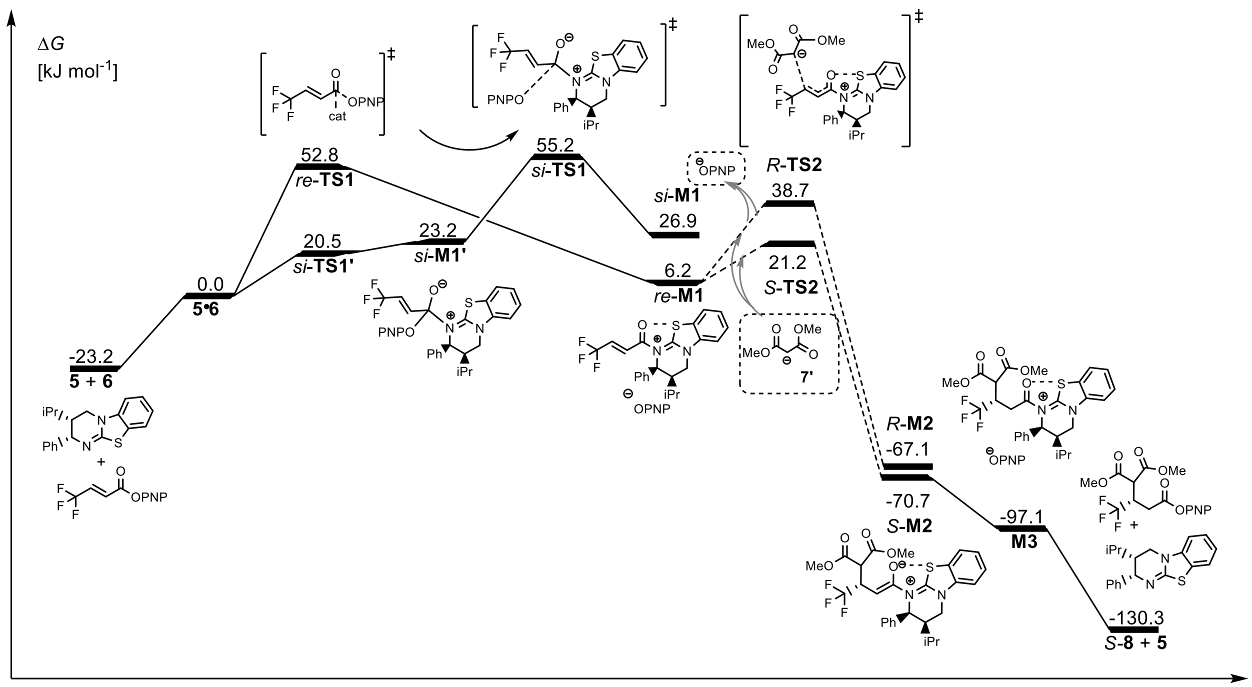

Figure S1: Reaction profile for Michael addition of dimethyl malonate (**7'**) to α,β-unsaturated ester (**6**) catalysed by HyperBTM (**5**) at 273.15 K, M06-2X-D3/6-311++G(2df,2dp)/ IEFPCM$_{THF}$//M06-2X/6-31G(d,p)/IEFPCM$_{THF}$ level.

Following the procedure from Wang et al.[13] the free energies are reported relative to an encounter complex between catalyst **5** and our model reactant **6** (R = CF_3 in Scheme 4 in the main paper), denoted **5•6**. This is to minimise artifacts from entropies of associative steps that are evaluated from standard thermodynamic expressions based on ideal-gas approximation. At 0°C, formation of **5•6** is computed endergonic by ΔG = 23.2 kJ mol^{-1}.[17] The first key intermediate, **M1**, is obtained as contact ion pair between a cationic isothiouronium complex and the OPNP^{-} leaving group. Depending on which face of the prochiral enone moiety the phenolate is located, two diastereomeric forms are possible, of which the *si* form is less stable despite a potentially favourable π–π interaction (see Figure S2), presumably because the steric clash between the two aromatic moieties leads to an unfavourably large charge separation in the zwitterion.

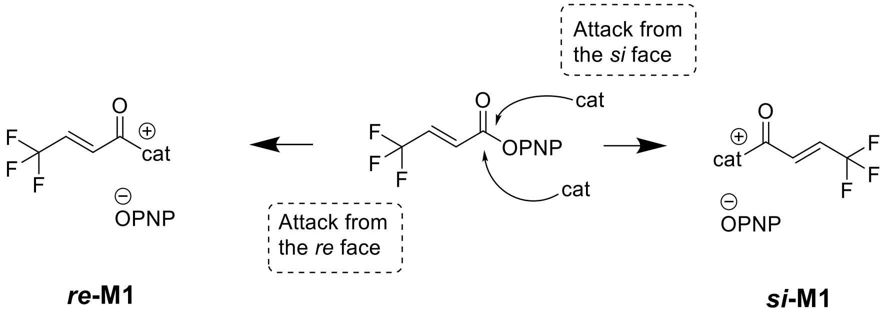

(a)

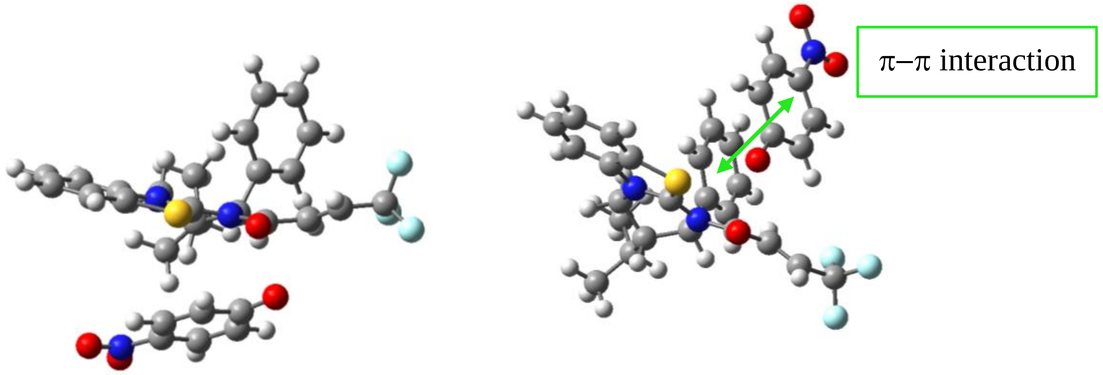

(b)

Figure S2: (a) Schematic sketch of formation of *re*- and *si*-**M1**; (b) three-dimensional plot of *re*- and *si*-**M1**, the latter is less stable despite the highlighted π–π interaction.

Two transition states were located leading from **5•6** to *re*- **M1** and *si*-**M1**, labelled *re*-**TS1** and *si*-**TS1**, respectively, in Figure S1. It turned out that one of them, *si*-**TS1**, does not connect to the reactant complex directly, but to a shallow minimum, a tetrahedral intermediate (*si*-**M1'**). The barrier connecting this intermediate to **5•6** is so low on the potential energy surface, however, that after the single-point energy and thermodynamic corrections the corresponding transition state (*si*-**TS1'**) is lower in free energy than *si*-**M1'**, indicating that both exist only as points along the reaction pathway, not as stationary points proper. The lowest barrier leading to one of the diastereomeric **M1** intermediates (the more stable of the two, actually), is found via *re*-**TS1** at $\Delta G^{\ddagger} = 52.8$ kJ mol^{-1}.

Again following the protocol of Wang et al.[13] we assume facile exchange between the phenolate and the Michael nucleophile, the deprotonated malonate ester (**7'**), and have located the transition states for attack of the latter at the β-carbon of the α, β-unsaturated ketone moiety in **M1**, as well as the resulting zwitterionic intermediates, **M2**. This is the point where the stereochemistry of the final product, (*R* or *S*) is determined.

In addition to the stereochemistry in the product, there is some conformational flexibility about the newly formed C-C single bond. We have trialled several such conformations (for both *R* and *S* intermediates **M2** and transition states, **TS2**) and report only the results for the most stable of each in Figure S1.

It turns out that *S*-**M2** is more stable than *R*-**M2**, but only by $\Delta\Delta G$ = 3.6 kJ mol^{-1}. The steric clash that, arguably, favours *S*-**M2** over *R*-**M2** is illustrated in Figure S3.

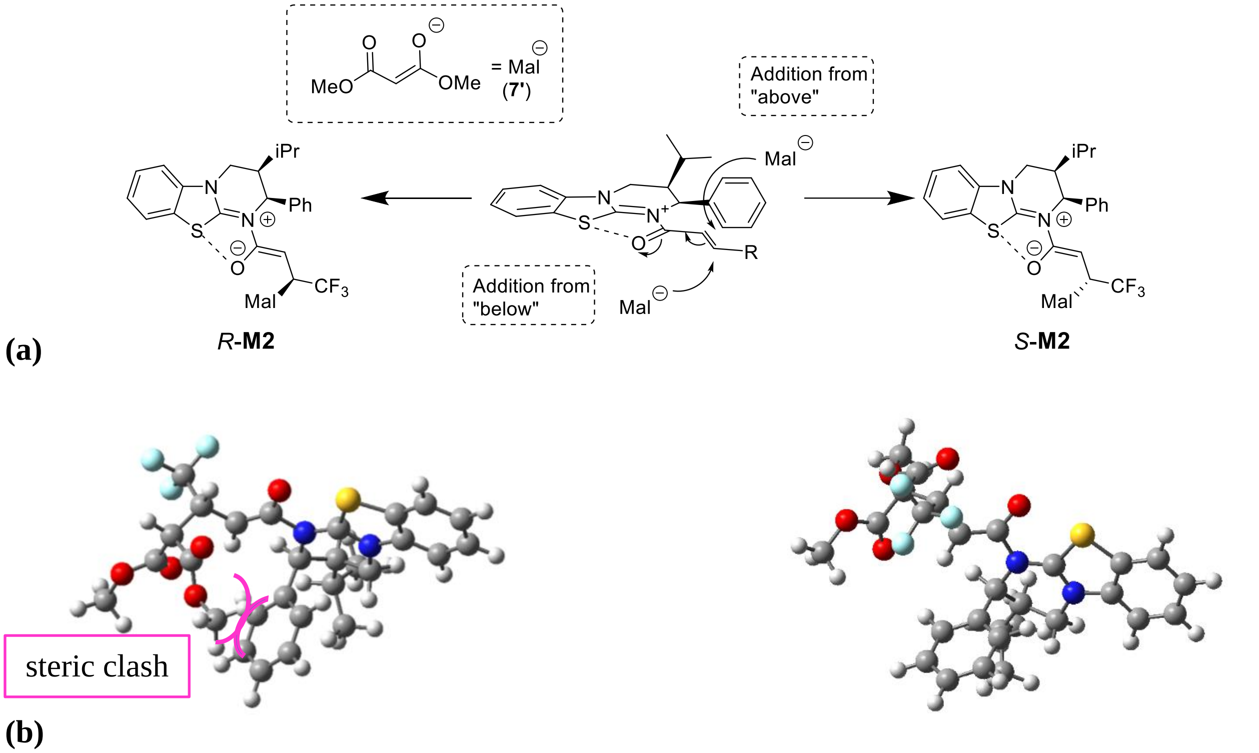

Figure S3: (a) Schematic sketch of formation of *R*- and *S*-**M2**; (b) three-dimensional plot of *R*- and *S*-**M2**, the latter is slightly more stable because of the steric clash highlighted.

There is also some conformational flexibility in the malonate moiety itself. In the **M2** products, like in the free neutral malonate ester **7**, the two carbonyl groups adopt a gauche conformation (as opposed to an anti orientation with C_2- or pseudo C_2-symmetry). These conformations would connect to the deprotonated malonate **7'** in its *cis* configuration (C_{2v}-symmetry), however for the free anion **7'**, the *trans* configuration (C_s-symmetry) turned out to be slightly more stable (by ΔG = -4.9 kJ mol^{-1}).[11] We located a total of 10 transition states for **TS2** involving both *cis*-**7'** and *trans*-**7'**; the relative free energies of these TSs are collected in Table S1. Those leading to *S* products tend to be more stable than those leading to *R*. The free energy difference between the lowest of each, *S*-**TS2** and *R*-**TS2** (both involving *trans*-**7'**), is $\Delta\Delta G^{\ddagger}$ = 17.5 kJ mol^{-1}.

Table S1: Optimised C···C distances (M06-2X/6-31G(d,p)/IEFPCM$_{THF}$ level) and relative free energies (M06-2X-D3/6-311++G(2df,2dp)/ IEFPCM$_{THF}$//M06-2X/6-31G(d,p)/IEFPCM$_{THF}$ level, 273.15 K)

Nucleophile	TS	$r_{C\cdots C}$ [Å]	$\Delta\Delta G^{\ddagger}_{rel}$ [kJ mol^{-1}]
trans-**7'**	*S*-**TS2**	2.40	0.0
"	*S*-**TS2a**	2.43	1.2
"	*S*-**TS2b**	2.51	5.2
"	*S*-**TS2c**	2.56	24.3
"	*R*-**TS2**	2.57	17.5
"	*R*-**TS2a**	2.62	19.4
cis-**7'**	*S*-**TS2d**	2.54	10.3
"	*S*-**TS2e**	2.60	18.5
"	*R*-**TS2b**	2.62	19.2
"	*R*-**TS2c**	2.63	23.4

The structures of *S*-**TS2** and *R*-**TS2** are shown in Figure S4 (which is a stereo version of Figure 1 in the main paper). A similar steric clash as in the product *R*-**M2** (Figure S3) is seen in *R*-**TS2** (Figures 1 and S4). It is arguably this clash that causes an elongated C···C distance in that TS (2.57 Å at the M06-2X level), compared to the same distance in *S*-**TS2** (2.40 Å). In fact, there appears to be a loose correlation between that distance and the barrier height, the latter tending to increase with the former (see $r_{C\cdots C}$ values in Table S1).

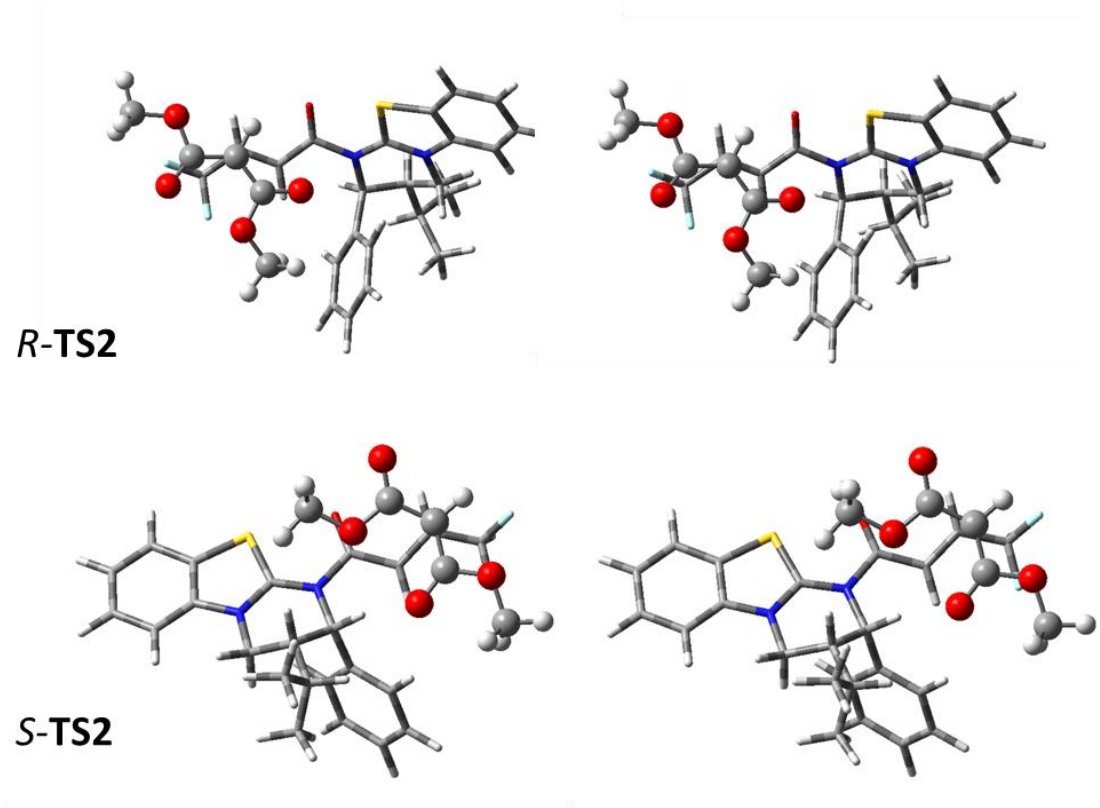

Figure S4: Stereoplot of *S*- and *R*-**TS2** (M06-2X/6-31G(d,p)/IEFPCM optimized, malonate rendered as ball-and-stick, HyperBTM as tube).

Note that *S*- and *R*-**TS2** do not connect directly to *S*- and *R*-**M2**, but to slightly higher-lying rotamers (*S*-**M2'** and *R*-**M2'**, not shown), which can convert to the more stable intermediates *S*-**M2** and *R*-**M2** via simple rotation about C-C single bonds. This step, addition of the malonate, is computed so exergonic (e.g. ΔG = -78.9 kJ mol^{-1} from *re*-**M1** to *S*-**M2**) that the barriers for the reverse reaction are essentially unsurmountable under the mild reaction conditions (e.g. $\Delta G^{\ddagger}$ = 91.9. kJ mol^{-1} from *S*-**M2** to *S*-**TS2**). This addition is therefore irreversible and under kinetic control. The stereochemistry of the final product should thus be determined by the free-energy difference between the (selectivity-determining) transition states *S*-**TS2** and *R*-**TS2**. This aforementioned difference of $\Delta\Delta G^{\ddagger}$ = 17.5 kJ mol^{-1} corresponds to a computed *er* of 99.95:0.05 at 273.15 K, in excellent agreement with the experimental values exceeding 99:1 (see Table 1 in the main paper). For a full prediction of the stereocontrol one could calculate the amount of *S*- and *R*-product from appropriate Boltzmann averages over all relative barriers leading to these products ($\Delta\Delta G^{\ddagger}_{rel}$ values in Table S1, weighted by the Boltzmann equilibrium of nucleophiles *cis*-**7'** and *trans*-**7'**), which would afford a very similar outcome, namely essentially exclusive formation of *S*-product.[19]

The reaction is completed by the protonation of the enolate moiety in **M2** and substitution of the HyperBTM moiety with the aryl enolate, affording the final product (**8**) and regenerating the organocatalyst (**5**). Starting from the lowest intermediate *S*-**M2**, the intermediate of the first step of this sequence was modelled via reaction with free aryl phenol, HOPNP affording a contact ion pair *S*-**M3** (again following the procedure of Wang et al.,[13] in order to avoid artifacts from charge separation with the simple solvation model). The energetics of this step include the driving force for formation of the phenol via deprotonation of the neutral malonate ester (**7**), according to

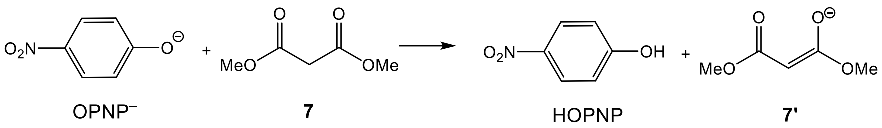

, ΔG = 54.4 kJ mol^{-1} (eq 2),

which produces the Michael nucleophile **7'** needed earlier. Formation of *S*-**M3** and its decay into the final products, *S*-**8** and **5**, are computed to be so favourable (with driving forces for each elementary step between $\Delta G \approx$ -26 to -33 kJ mol^{-1}, see the last two steps on the profile in Figure S1) that no kinetic hindrance and, thus, no bearing on the stereocontrol is to be expected. Therefore, no other stereoisomers were considered for **M3** and no transition states connecting to it were located at this stage.

7. References

[1] Morrill, L. C.; Douglas, J.; Lebl, T.; Slawin, A. M. Z.; Fox, D. J.; Smith, A. D. *Chem. Sci.* **2013**, *4*, 4146-4155.

[2] Greenhalgh, M. D.; Qu, S.; Slawin, A. M. Z.; Smith, A. D. *Chem. Sci.* **2018**, *9*, 4909–4918.

[3] Matviitsuk, A.; Greenhalgh, M. D.; Antúnez, D. J. B.; Slawin, A. M. Z.; Smith, A. D. *Angew. Chem., Int. Ed.* **2017**, *56*, 12282–12287.

[4] Liu, H.; Slawin, A. M. Z.; Smith, A. D. *Org. Lett.* **2020**, *22*, 1301–1305.

[5] CrystalClear-SM Expert v2.1. Rigaku Americas, The Woodlands, Texas, USA, and Rigaku Corporation, Tokyo, Japan, 2015

[6] CrysAlisPro v1.171.38.46. Rigaku Oxford Diffraction, Rigaku Corporation, Oxford, U.K. 2015

[7] Sheldrick, G. M. *Acta Crystallogr., Sect. A.* **2015**, *71*, 3–8.

[8] M. C. Burla, R. Caliandro, M. Camalli, B. Carrozzini, G. L. Cascarano, C. Giacovazzo, M. Mallamo, A. Mazzone, G., Polidori, R. Spagna, *J. Appl. Cryst*. **2012**, *45*, 357–361.

[9] Palatinus, L. Chapuis, G. *J. Appl. Cryst.* **2007**, *40*, 786–790.

[10] G. M. Sheldrick, *Acta Crystallogr., Sect. C.* **2015**, *71*, 3–8.

[11] CrystalStructure v4.3.0. Rigaku Americas, The Woodlands, Texas, USA, and Rigaku Corporation, Tokyo, Japan, 2018.

[12] M. J. Frisch, G. W. Trucks, J. R. Cheeseman, G. Scalmani, M. Caricato, H. P. Hratchian, X. Li, V. Barone, J. Bloino, G. Zheng, T. Vreven, J. A. Montgomery, G. A. Petersson, G. E. Scuseria, H. B. Schlegel, H. Nakatsuji, A. F. Izmaylov, R. L. Martin, J. L. Sonnenberg, J. E. Peralta, J. J. Heyd, E. Brothers, F. Ogliaro, M. Bearpark, M. A. Robb, B. Mennucci, K. N. Kudin, V. N. Staroverov, R. Kobayashi, J. Normand, A. Rendell, R. Gomperts, V. G. Zakrzewski, M. Hada, M. Ehara, K. Toyota, R. Fukuda, J. Hasegawa, M. Ishida, T. Nakajima, Y. Honda, O. Kitao, H. Nakai, Gaussian 09, Revision A.1, Gaussian Inc., Wallingford CT, 2009.

[13] Wang, C.; Li, S.-J.; Zhang, Q.-C.; Wei D.; Ding, L. *Catal. Sci. Technol*. **2020**, *10*, 3664–3669.

[14] (a) Zhao, Y.; Truhlar, D. G. *Acc. Chem. Res*. **2008**, *41*, 157‐167; (b) Zhao, Y.; Truhlar, D. G. *Theor. Chem. Acc*. **2008**, *120*, 215‐241.

[15] (a) Barone V.; Cossi, M. *J. Phys. Chem. A* **1998**, *102*, 1995‐2001. (b) Mennucci, B.; Tomasi, J. *J. Chem. Phys.* **1997**, *106*, 5151‐5158. (c) Scalmani, G,; Frisch, M. J. *J. Chem. Phys*. **2010**, *132*, 114110‐114125.

[16] Grimme, S.; Antony, J.; Ehrlich, S.;Krieg, H. *J. Chem. Phys*. **2010**, *132*, 154104‐154123.

[17] According to a Counterpoise calculation (Boys, S. F.; Bernardi, F. *Mol. Phys.* **1970,** *19*, 553-566) at the M06-2X/6-311++G(2df, 2dp) level, ca. 10 kJ mol^{-1} of this value is due to basis-set superposition error (BSSE). Because BSSE is relatively small and expected to be very similar for rate- and selectivity determining transitions states, no further BSSE corrections were applied.

[18] The cis and trans configurations of the deprotonated malonate ester are expected to be close in energy, as both have been characterized by X-ray crystallography, e.g.: (a) Huang, Z.; Hartwig, J. F. *Angew. Chem. Int. Ed.* **2 012**, *51*, 1028 –1032 (b) Zhang, Y.; Schmitt, M.; Falivene, L.; Caporaso, L.; Cavallo, L.; Chen, E. Y.-X. *J. Am. Chem. Soc.* **2013**, *135*, 17925−17942.

[19] Note that if only the slightly less stable form of the nucleophile (*cis*-**7'**) would have been considered, the corresponding lowest *S*- and *R*-TSs (*S*-**TS2d** and *R*-**TS2b**, respectively, in Table S1) would have afforded $\Delta\Delta G^{\ddagger}$ = 8.9 kJ mol^{-1}, corresponding to a computed *er* of 98:2 at 273.15 K in favour of the observed *S*-enantiomer, still in very good (albeit slightly worse) agreement with experiment.

8. Appendix 1: NMR Spectra

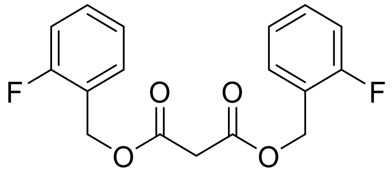

32

^{1}H, $CDCl_3$, 500 MHz

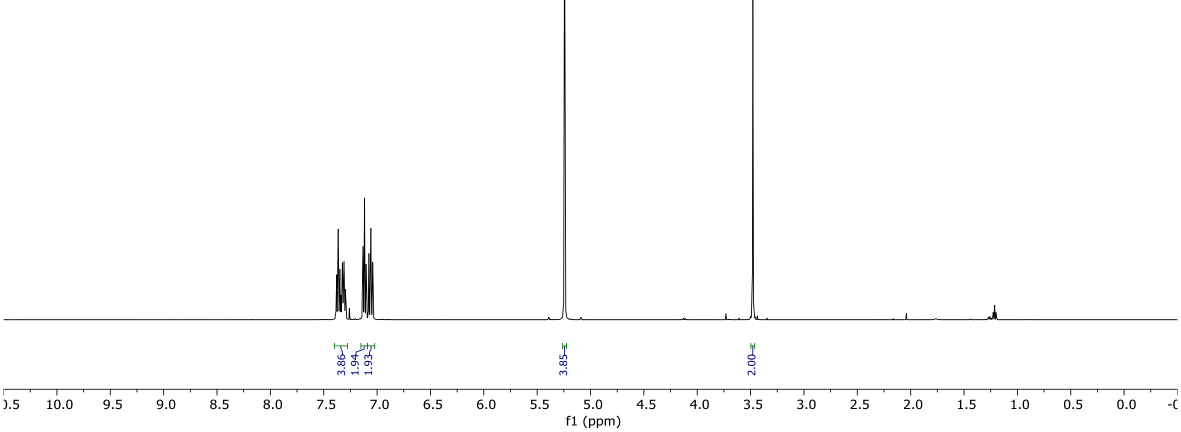

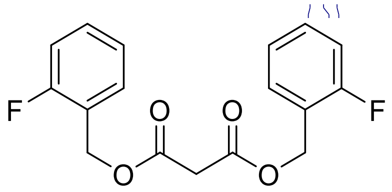

32

^{13}C {^{1}H}, $CDCl_3$, 126 MHz

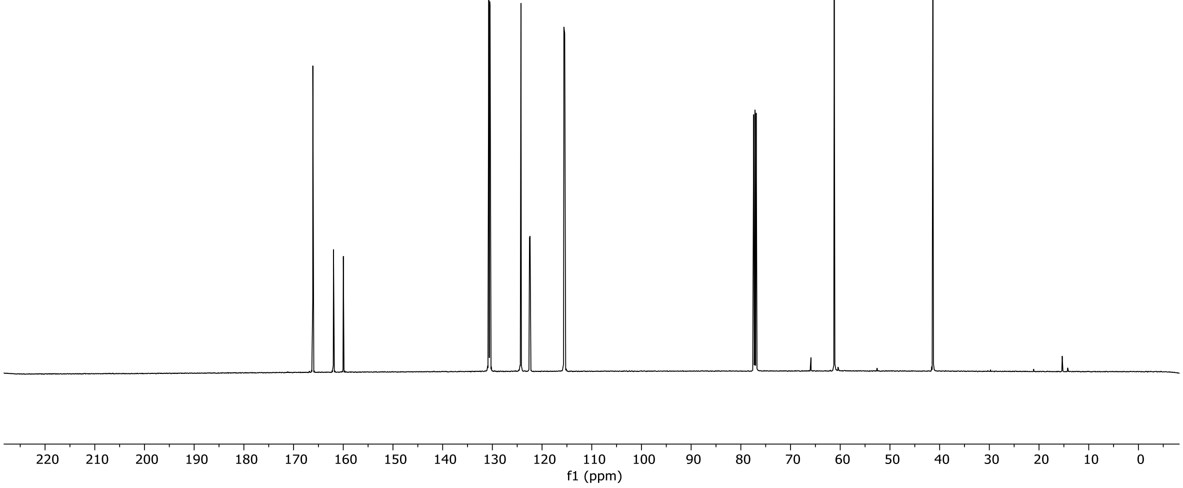

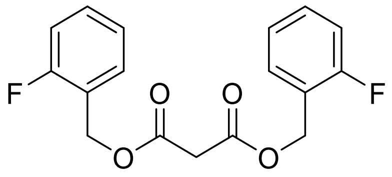

32

^{19}F, $CDCl_3$, 470 MHz

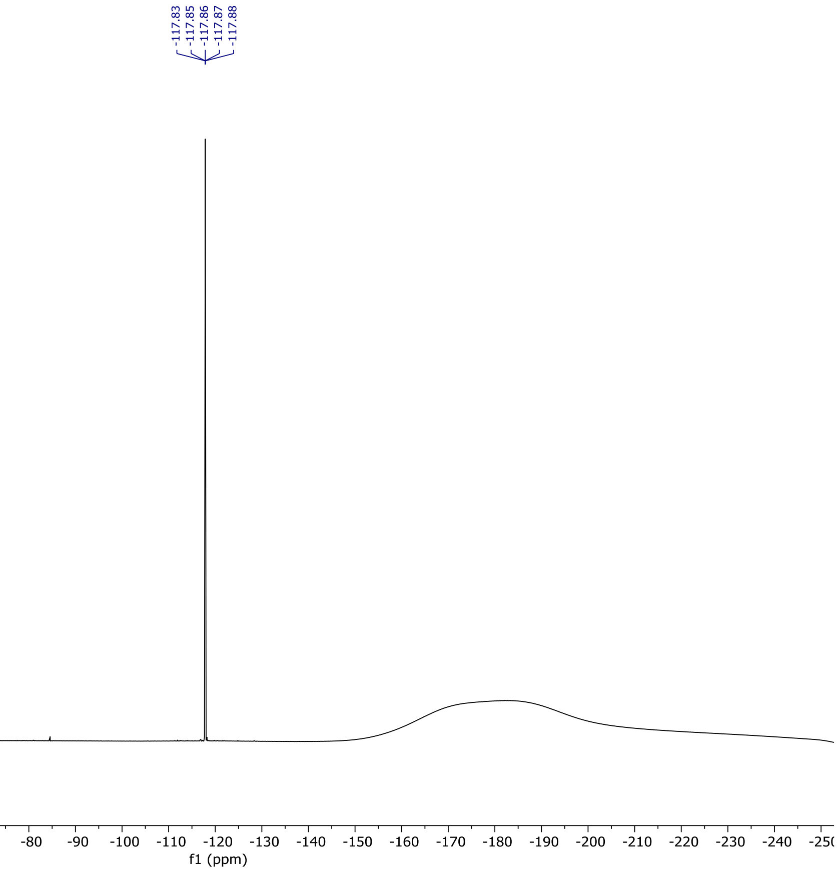

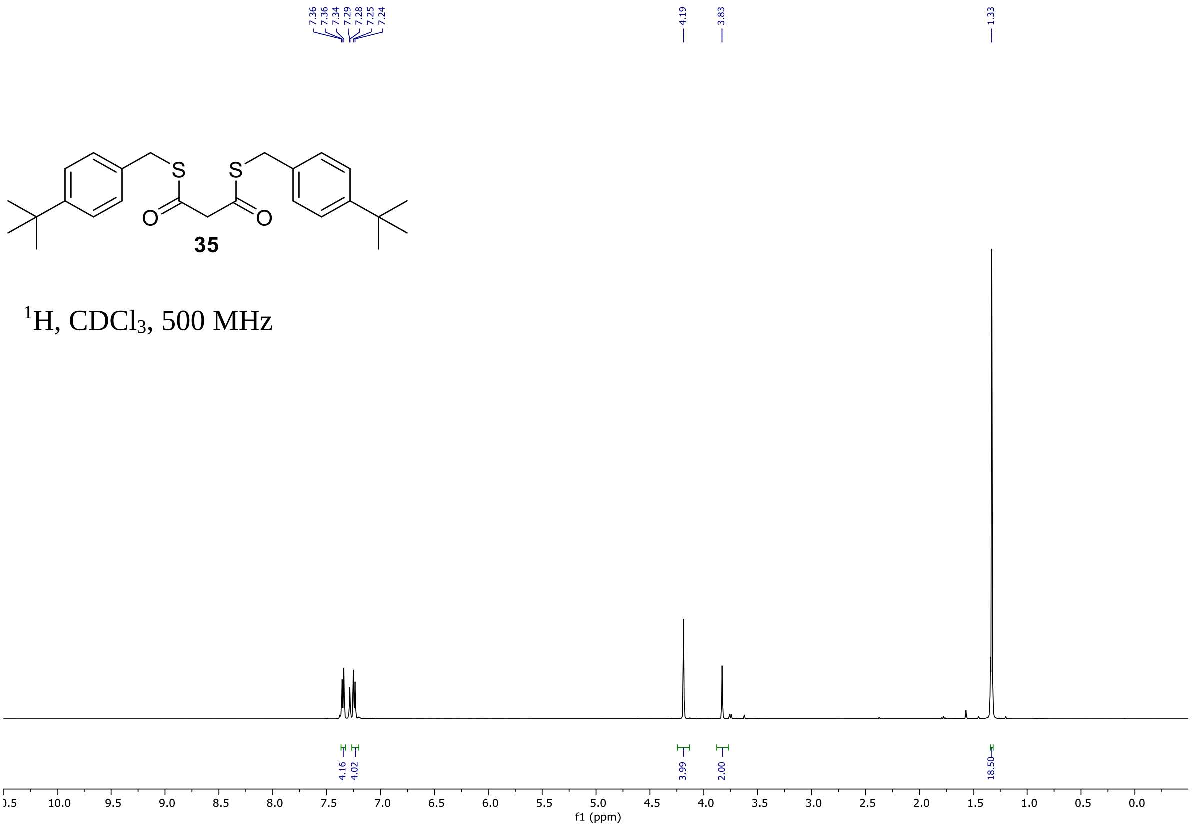
7.36 7.36 7.34 7.29 7.28 7.25 7.24
4.19
3.83
1.33
35
^{1}H, $CDCl_3$, 500 MHz
4.16 4.02 3.99 2.00 18.50
f1 (ppm)

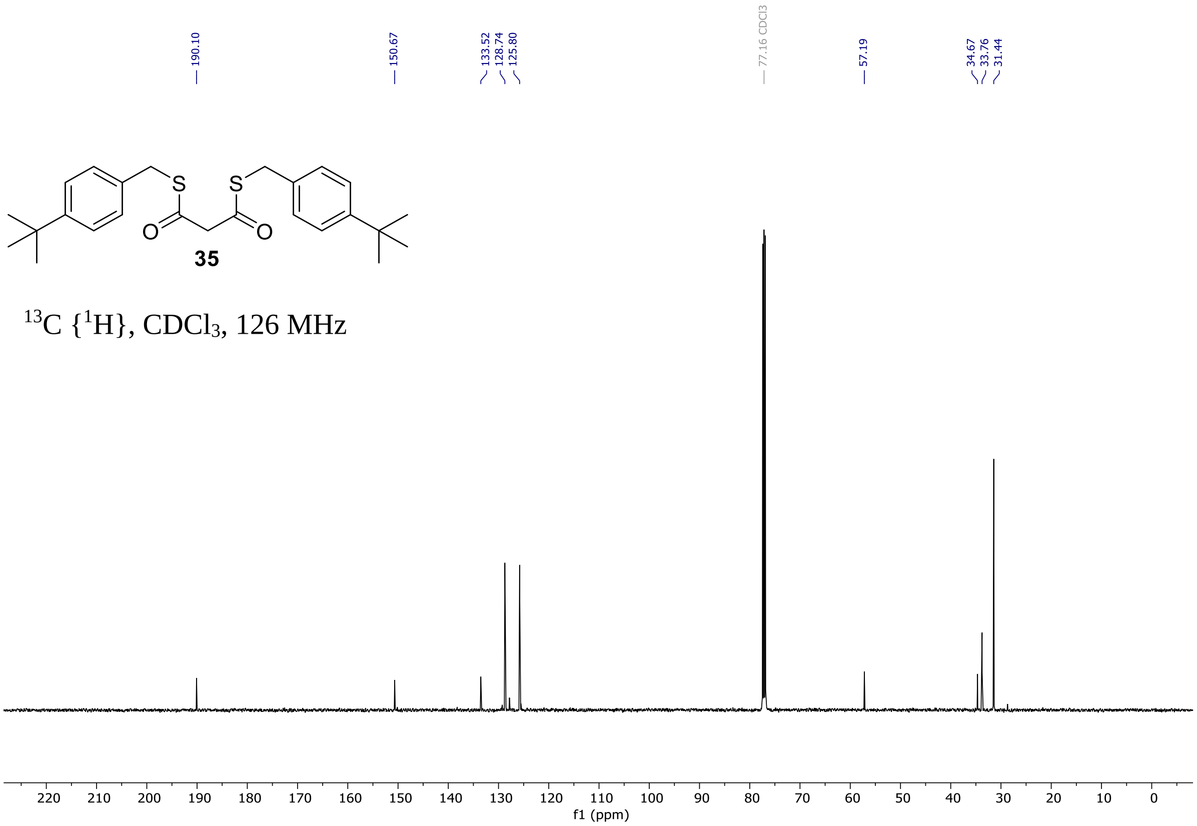
190.10
150.67
133.52 128.74 125.80
77.16 CDCl3
57.19
34.67 33.76 31.44
35
^{13}C {^{1}H}, $CDCl_3$, 126 MHz
f1 (ppm)

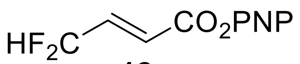

12

^{1}H, $CDCl_3$, 500 MHz

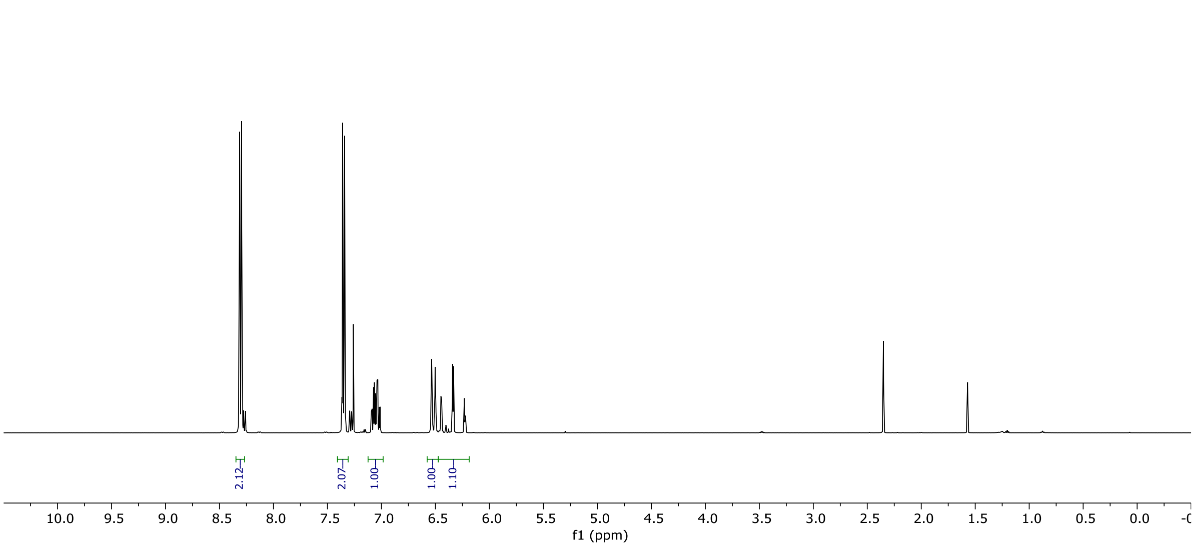

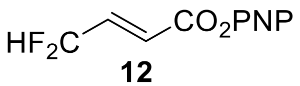

12

^{13}C {^{1}H}, $CDCl_3$, 126 MHz

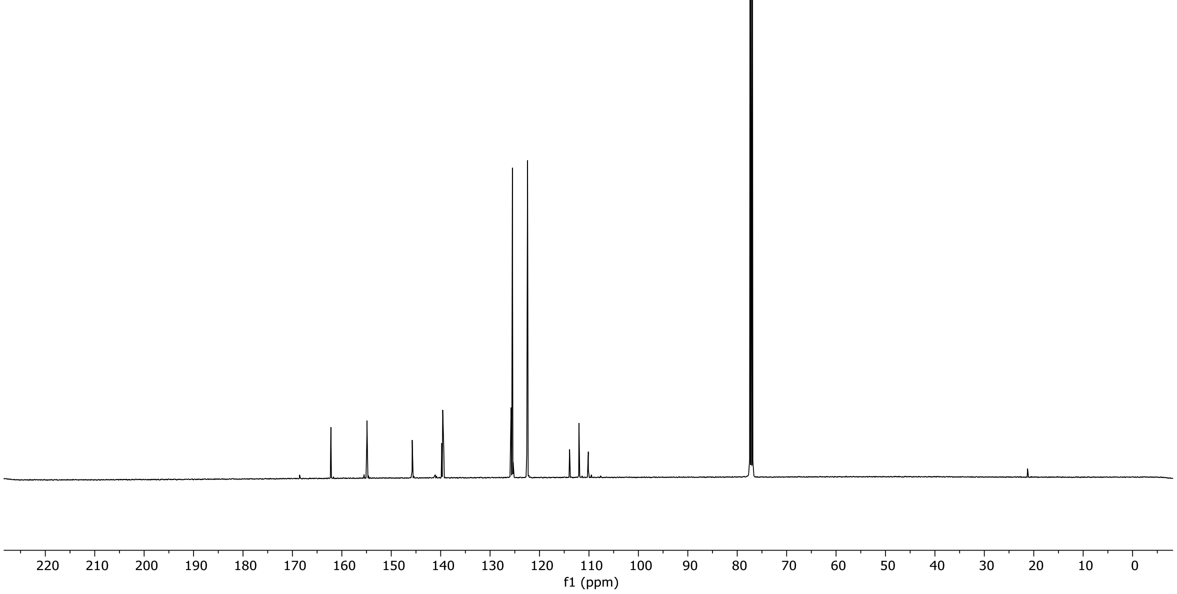

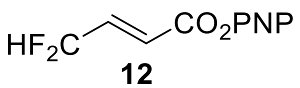

12

^{19}F, $CDCl_3$, 470 MHz

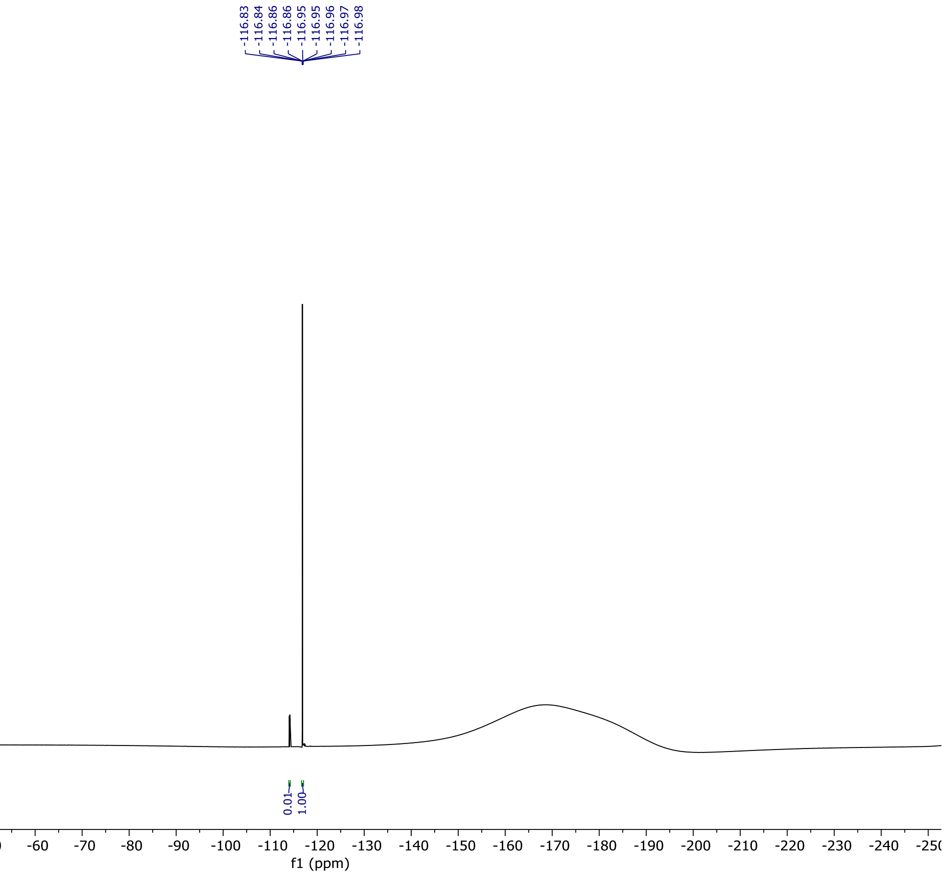

13

^{1}H, $CDCl_3$, 500 MHz

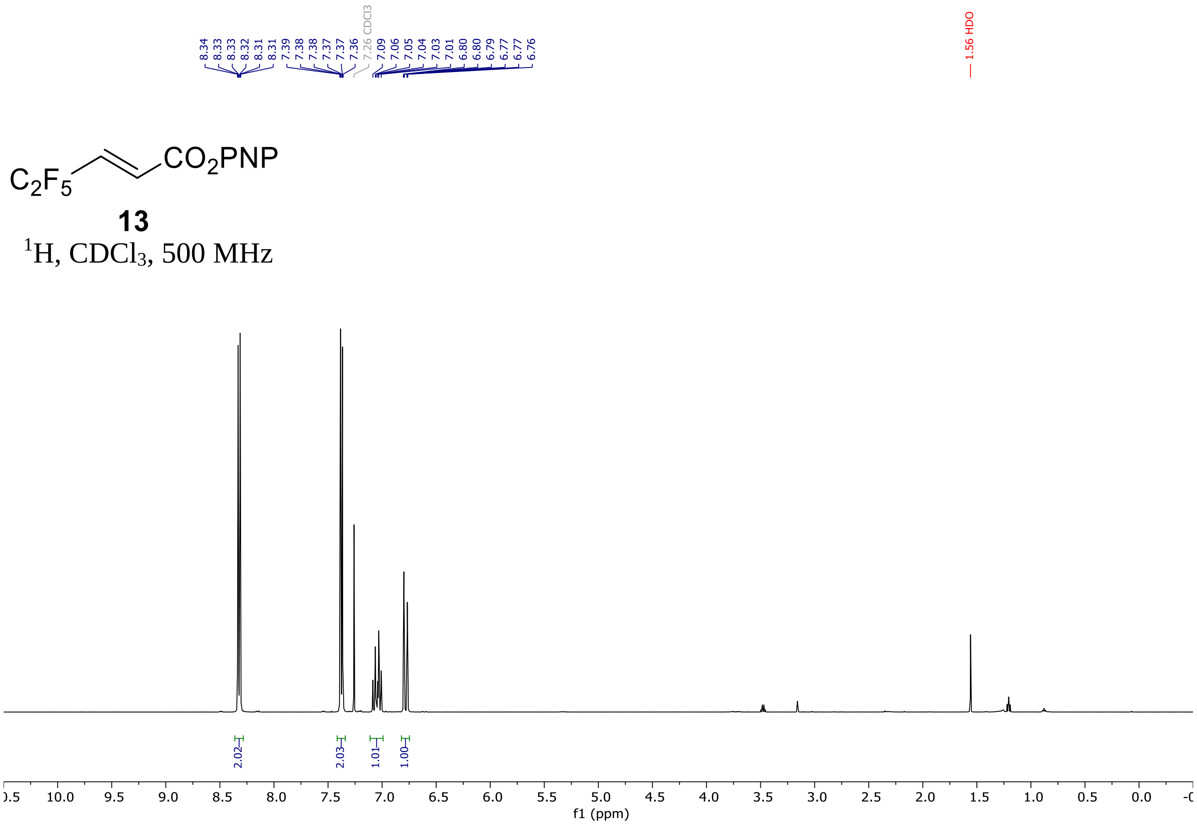

13

^{13}C {^{1}H}, $CDCl_3$, 126 MHz

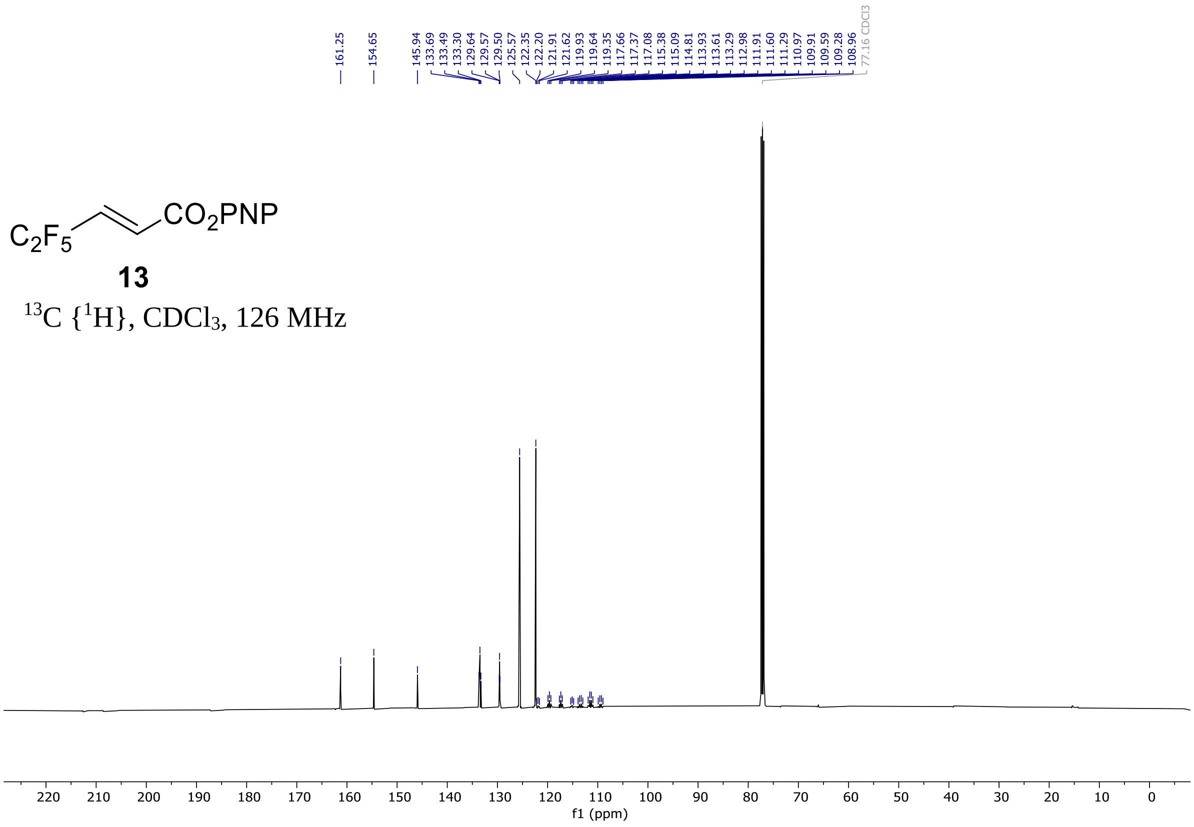

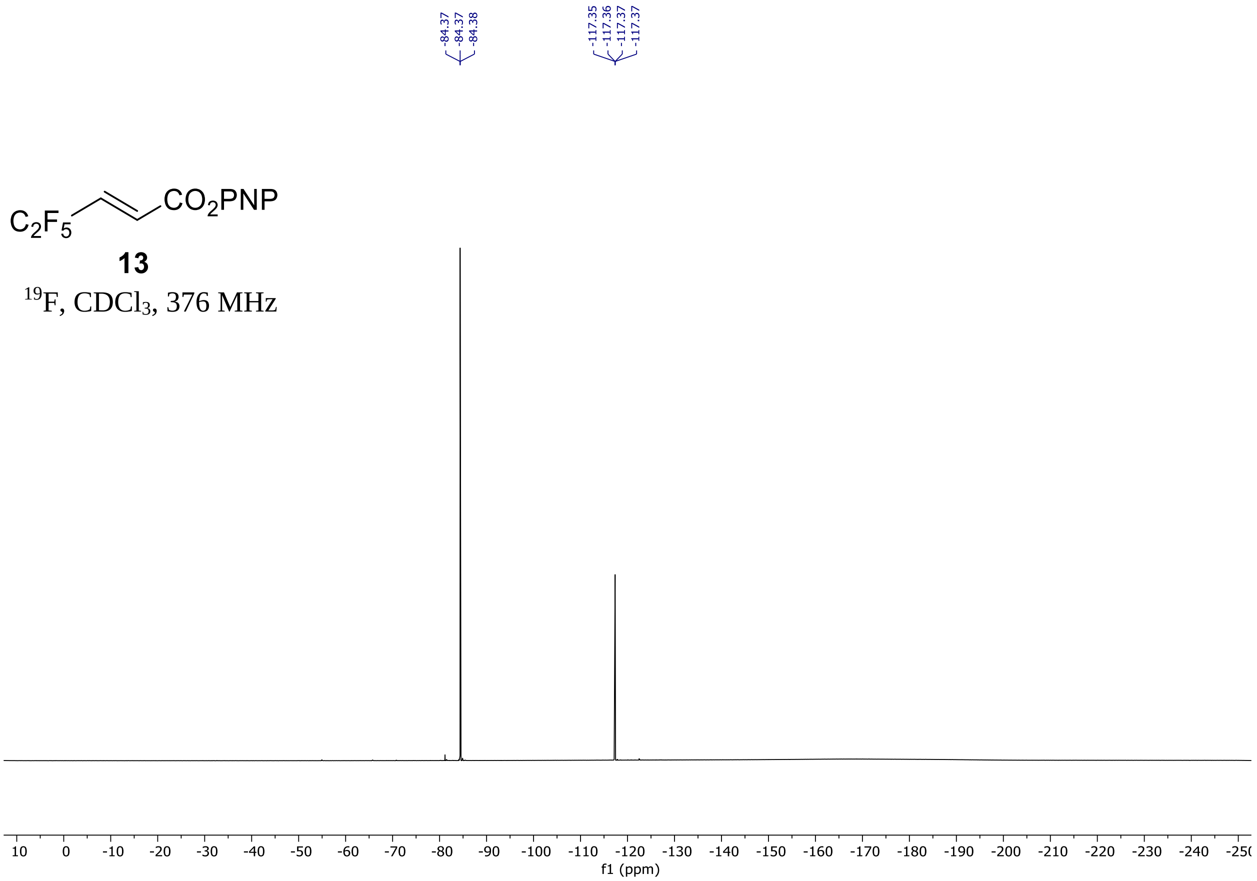

13

^{19}F, $CDCl_3$, 376 MHz

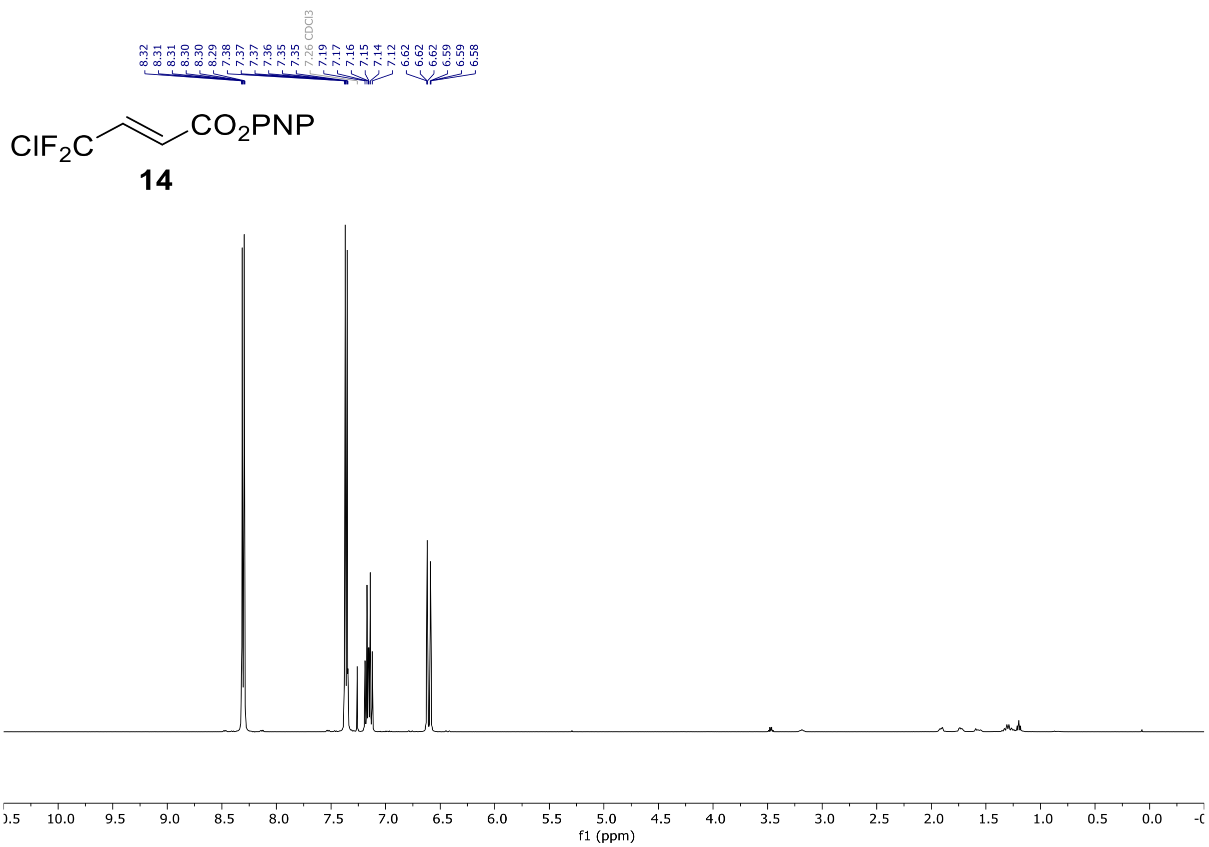

14

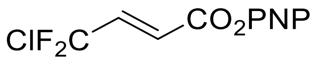

14

$^{13}C\{^{1}H\}$, $CDCl_3$, 126 MHz

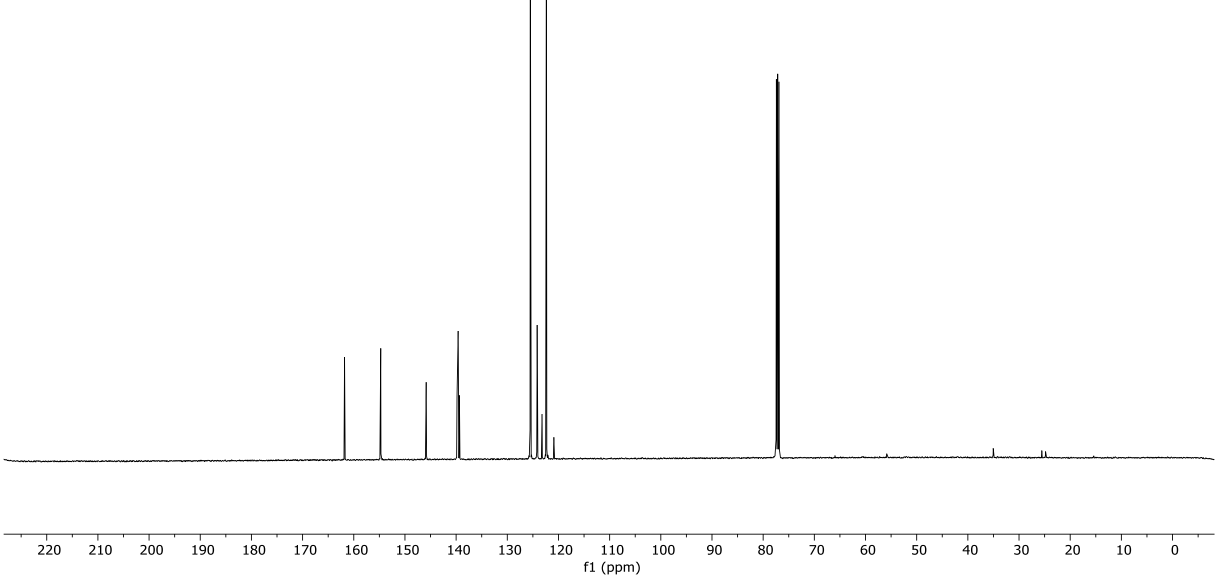

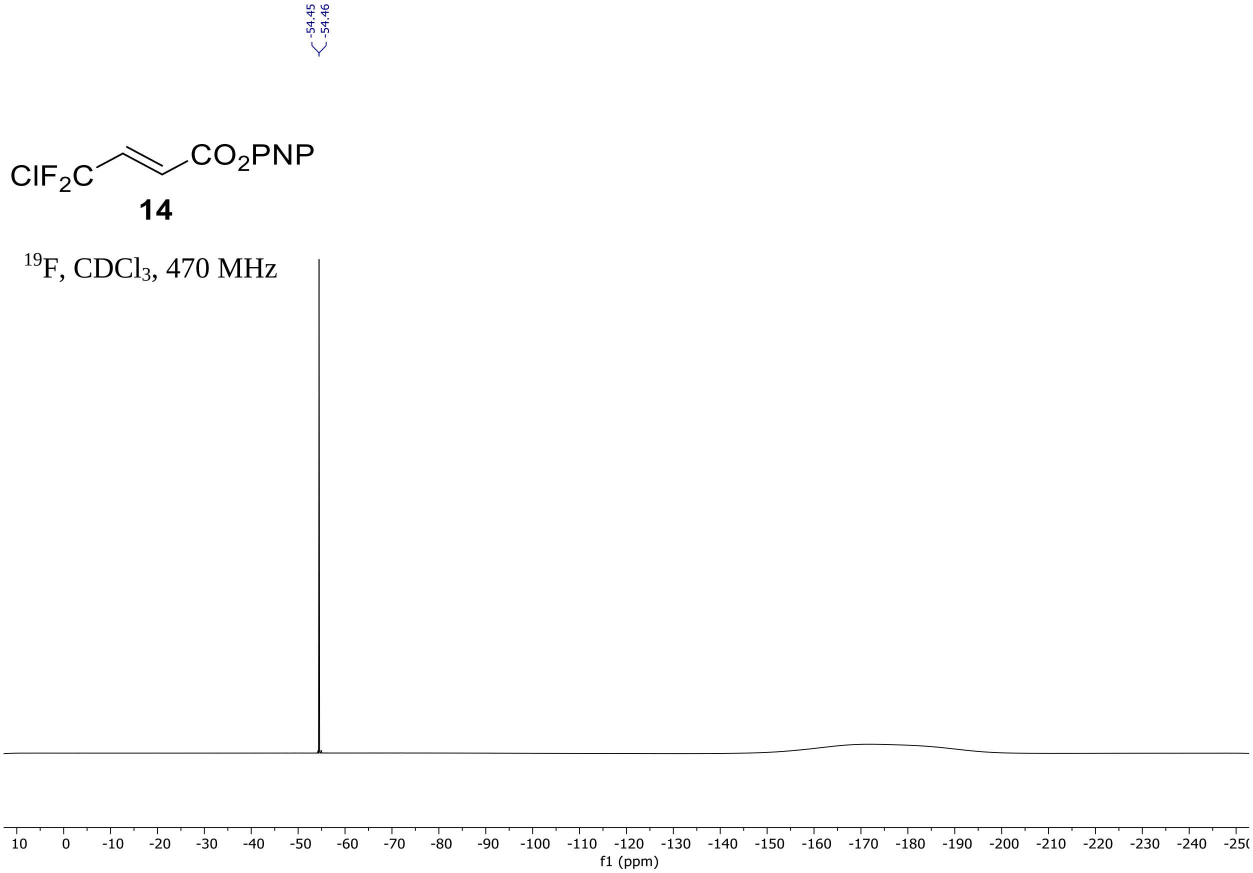

-54.45
-54.46
ClF2C CO2PNP
14
19F, CDCl3, 470 MHz
10 0 -10 -20 -30 -40 -50 -60 -70 -80 -90 -100 -110 -120 -130 -140 -150 -160 -170 -180 -190 -200 -210 -220 -230 -240
f1 (ppm)

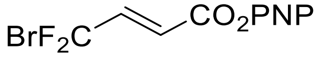

15

^{1}H, $CDCl_3$, 500 MHz

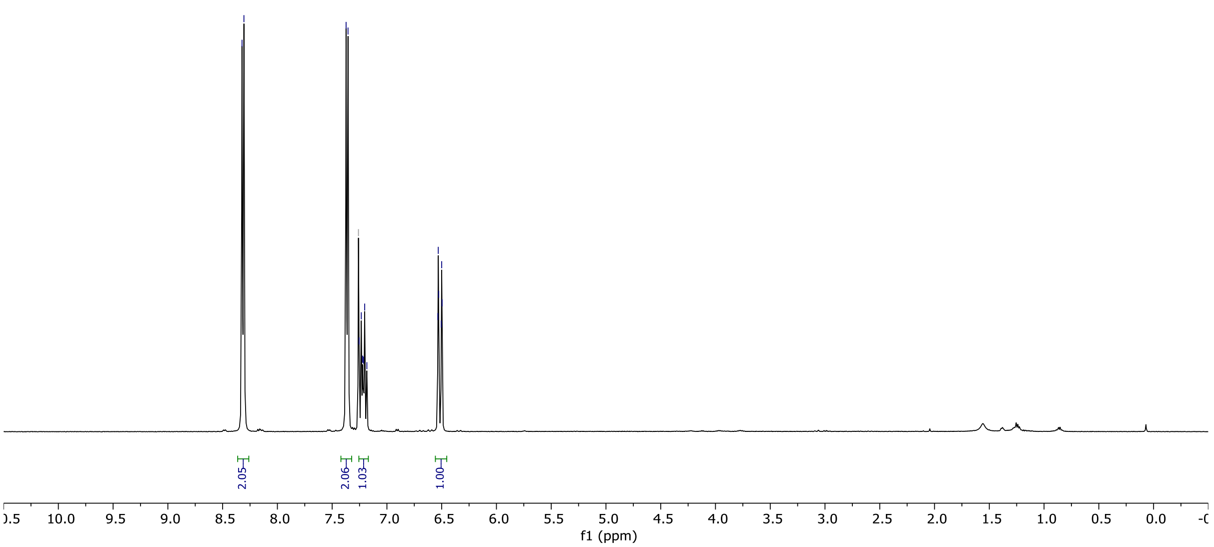

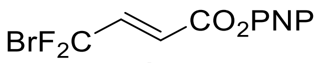

15

^{13}C {^{1}H}, $CDCl_3$, 126 MHz

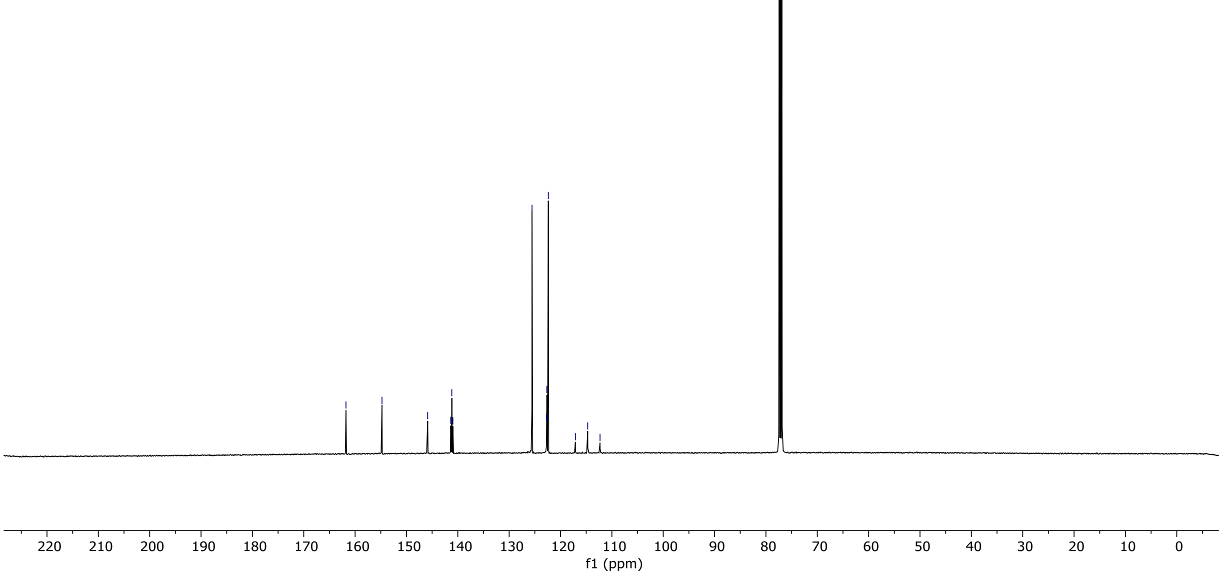

-50.61

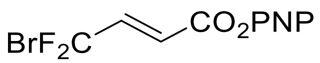

15

^{19}F, $CDCl_3$, 470 MHz

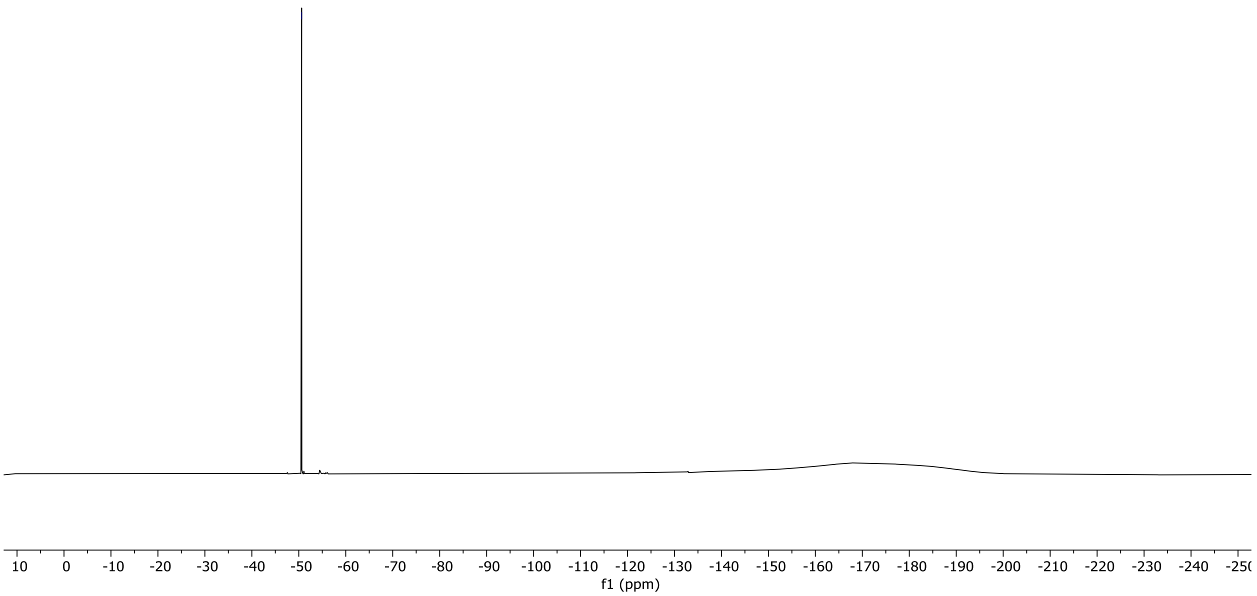

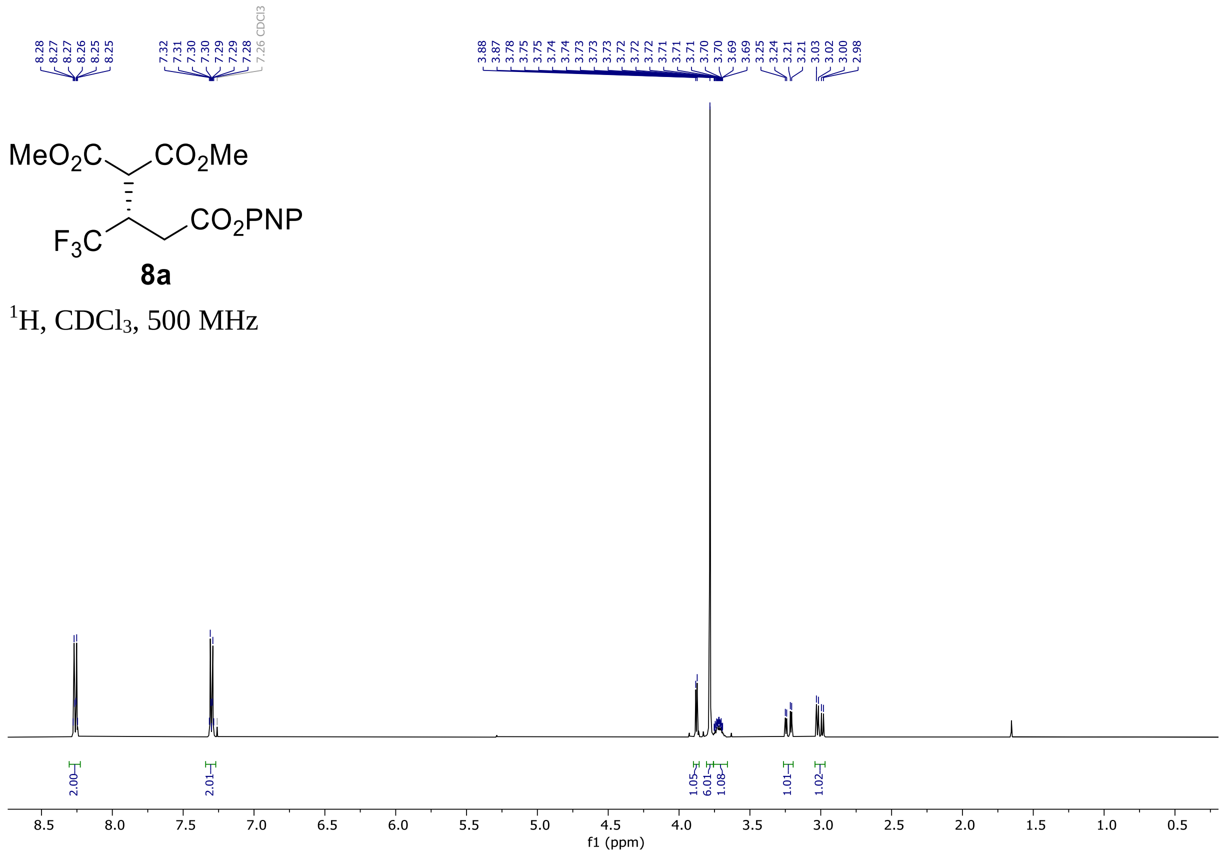
MeO2C CO2Me
F3C CO2PNP
8a
1H, CDCl3, 500 MHz
f1 (ppm)

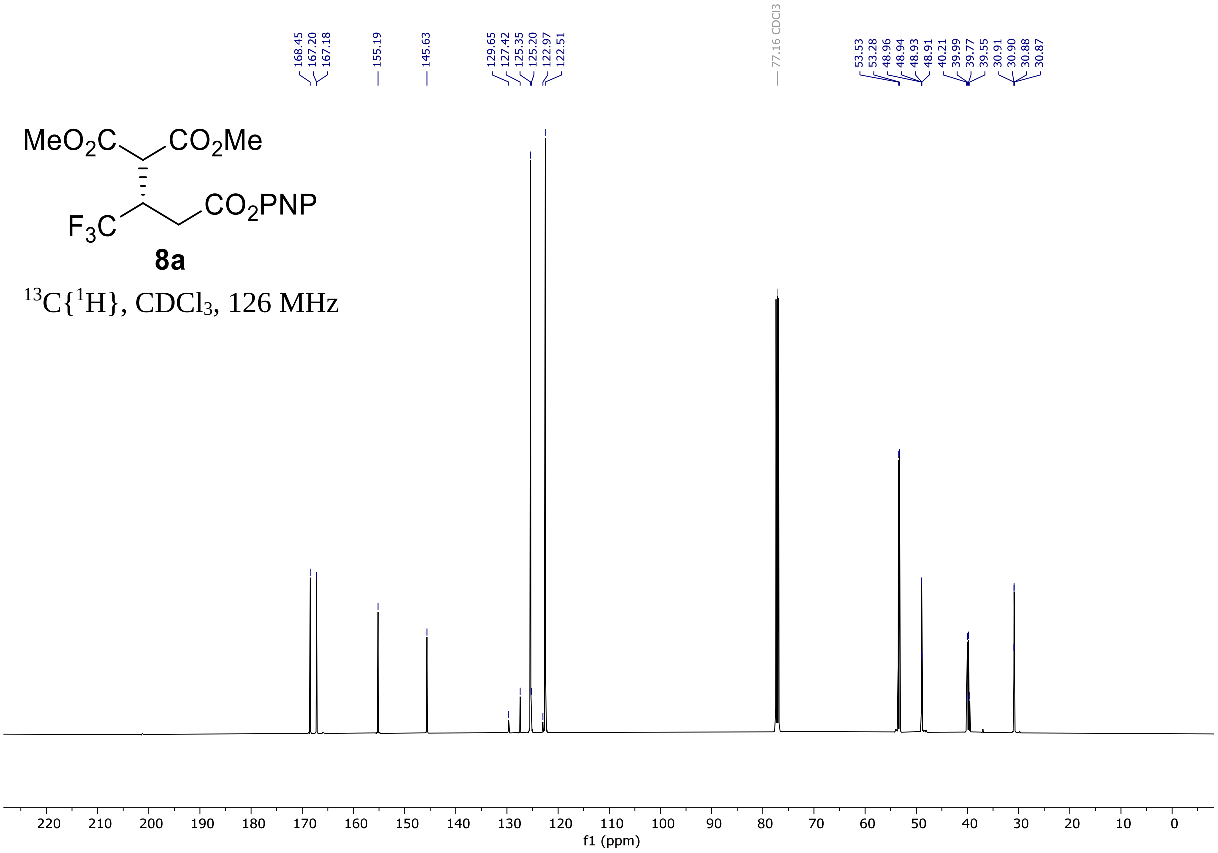
MeO2C CO2Me
F3C CO2PNP
8a
13C{1H}, CDCl3, 126 MHz
f1 (ppm)

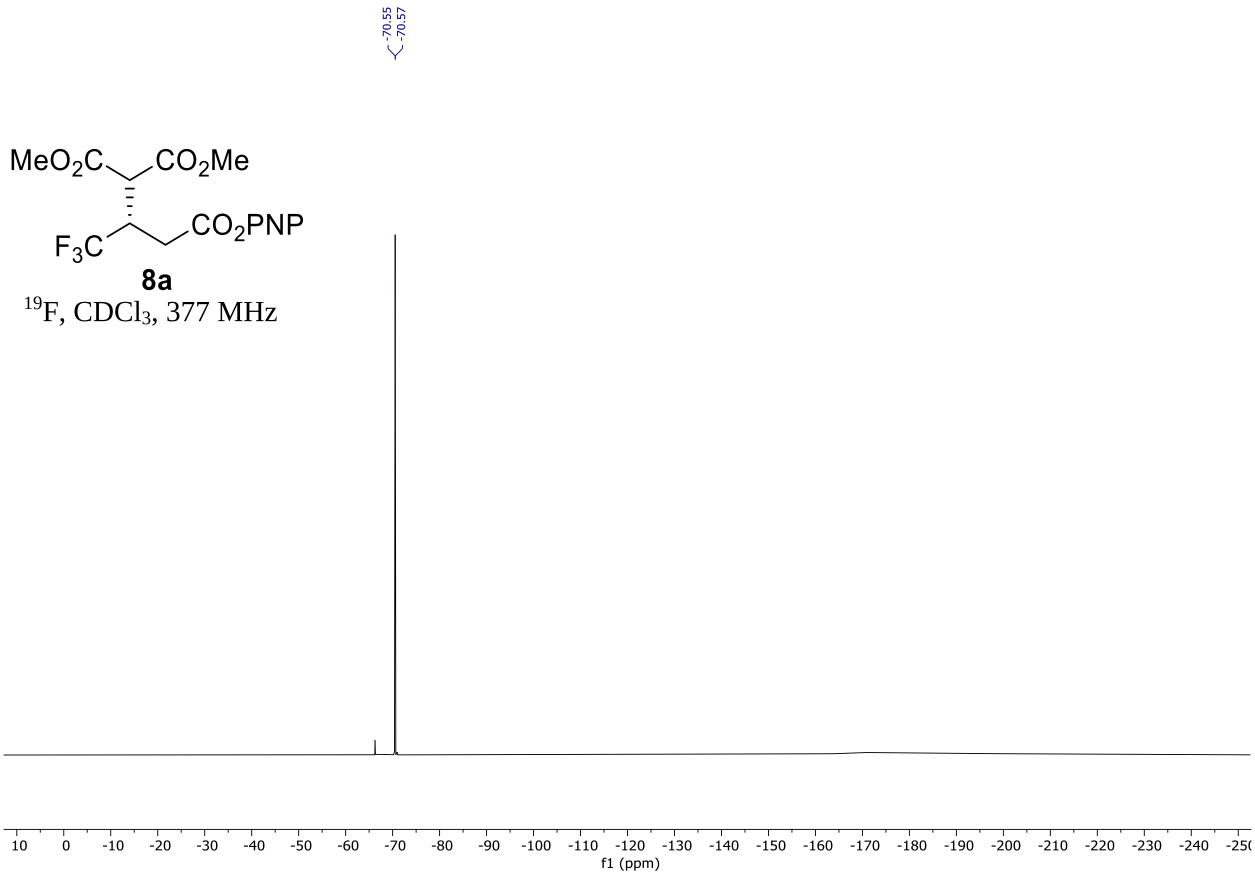
-70.55
-70.57
MeO_2C
CO_2Me
CO_2PNP
F_3C
8a
^{19}F, $CDCl_3$, 377 MHz
10 0 -10 -20 -30 -40 -50 -60 -70 -80 -90 -100 -110 -120 -130 -140 -150 -160 -170 -180 -190 -200 -210 -220 -230 -240 -250
f1 (ppm)

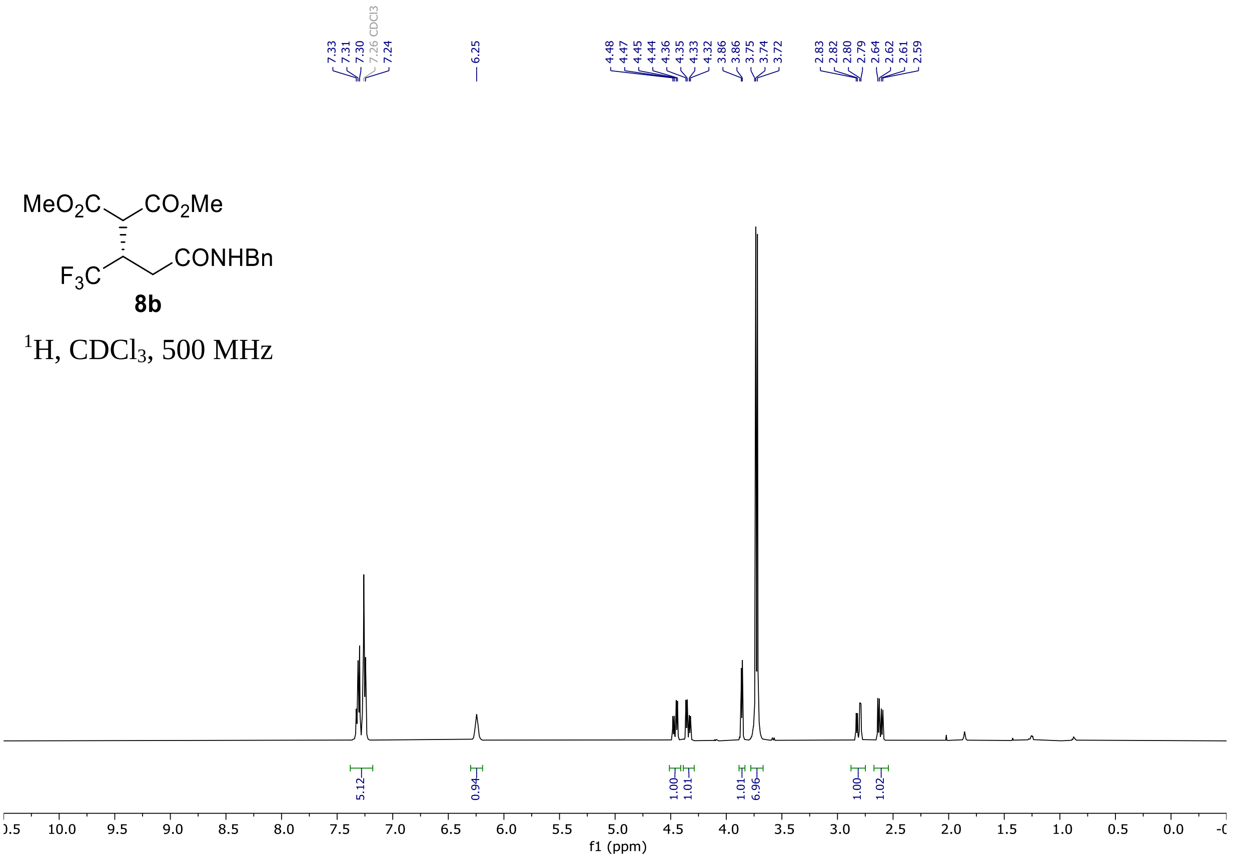

7.33
7.31
7.30
7.26 CDCl3
7.24
6.25
4.48
4.47
4.45
4.44
4.36
4.35
4.33
4.32
3.86
3.86
3.75
3.74
3.72
2.83
2.82
2.80
2.79
2.64
2.62
2.61
2.59
MeO2C
CO2Me
CONHBn
F3C
8b
1H, CDCl3, 500 MHz
5.12
0.94
1.00
1.01
1.01
6.96
1.00
1.02
f1 (ppm)

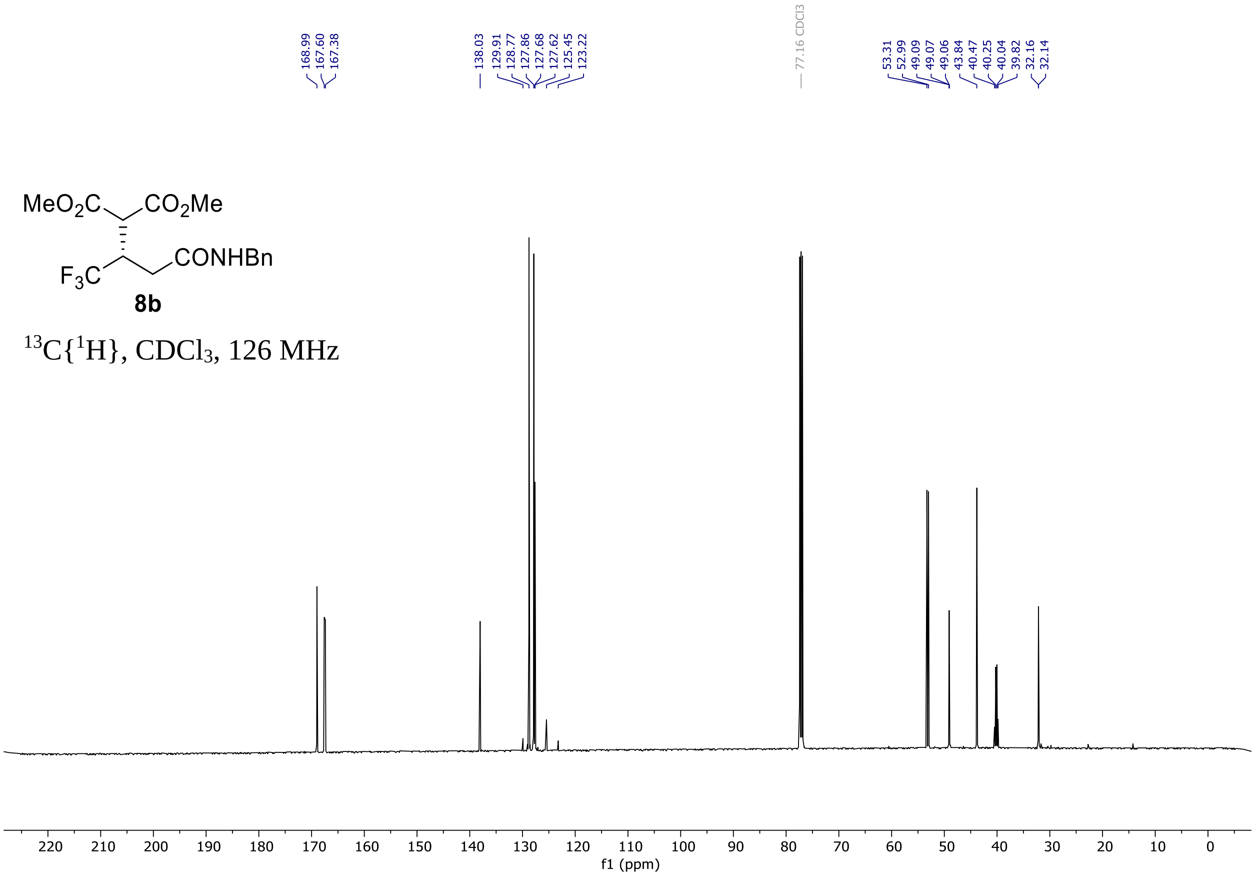

168.99
167.60
167.38
138.03
129.91
128.77
127.86
127.68
127.62
125.45
123.22
77.16 CDCl3
53.31
52.99
49.09
49.07
49.06
43.84
40.47
40.25
40.04
39.82
32.16
32.14
MeO2C
CO2Me
CONHBn
F3C
8b
13C{1H}, CDCl3, 126 MHz
f1 (ppm)

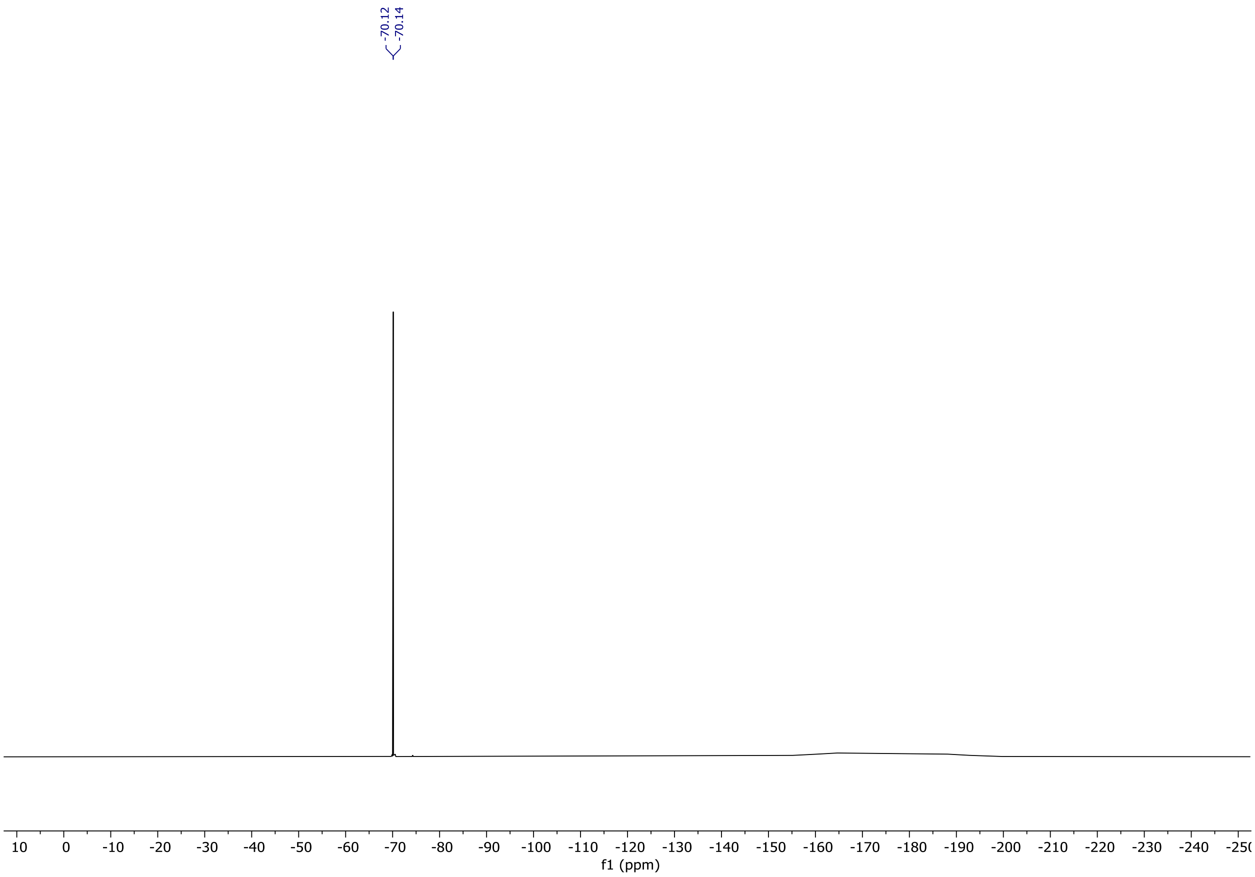

-70.12
-70.14
10
0
-10
-20
-30
-40
-50
-60
-70
-80
-90
-100
-110
-120
-130
-140
-150
-160
-170
-180
-190
-200
-210
-220
-230
-240
f1 (ppm)

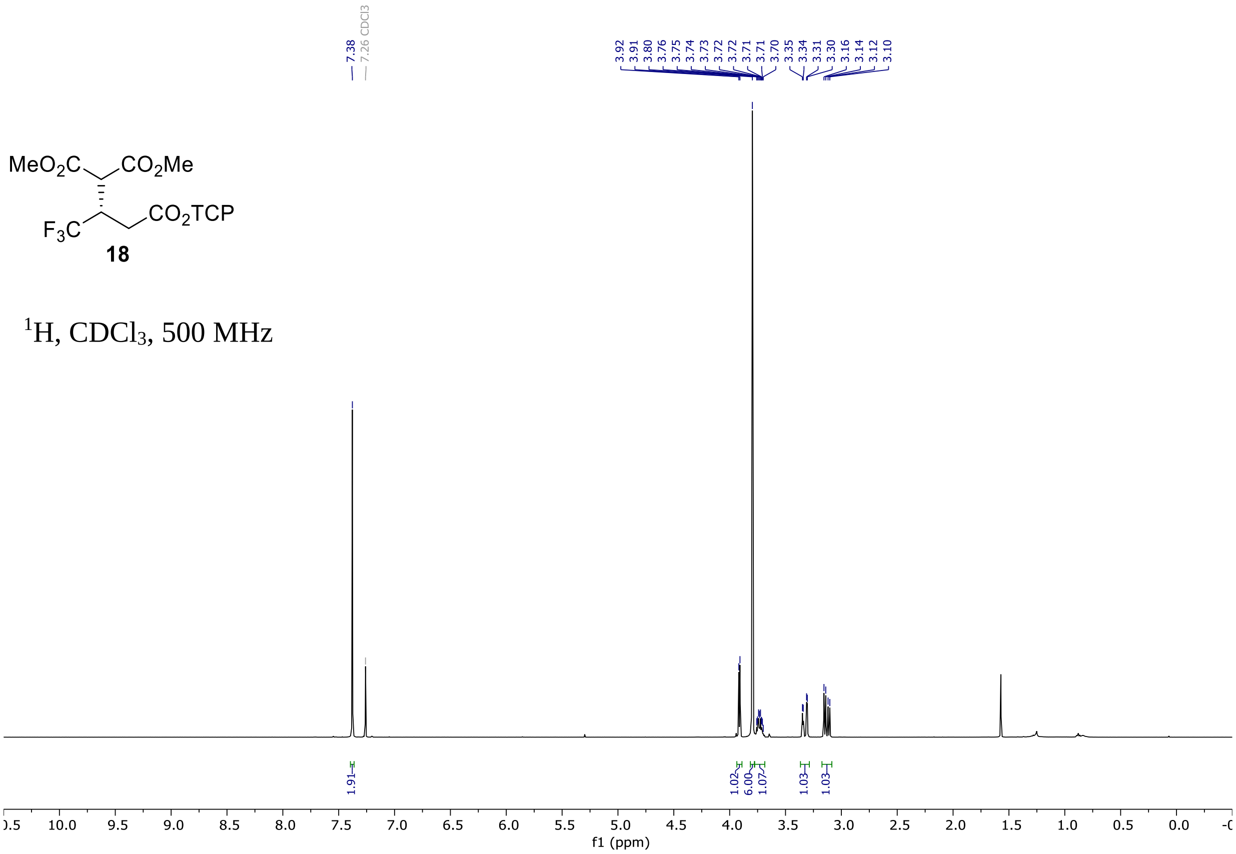
MeO2C CO2Me
F3C CO2TCP
18
1H, CDCl3, 500 MHz
7.38
7.26 CDCl3
3.92 3.91 3.80 3.76 3.75 3.74 3.73 3.72 3.72 3.71 3.71 3.70 3.35 3.34 3.31 3.30 3.16 3.14 3.12 3.10
1.91
1.02 6.00 1.07
1.03 1.03
f1 (ppm)

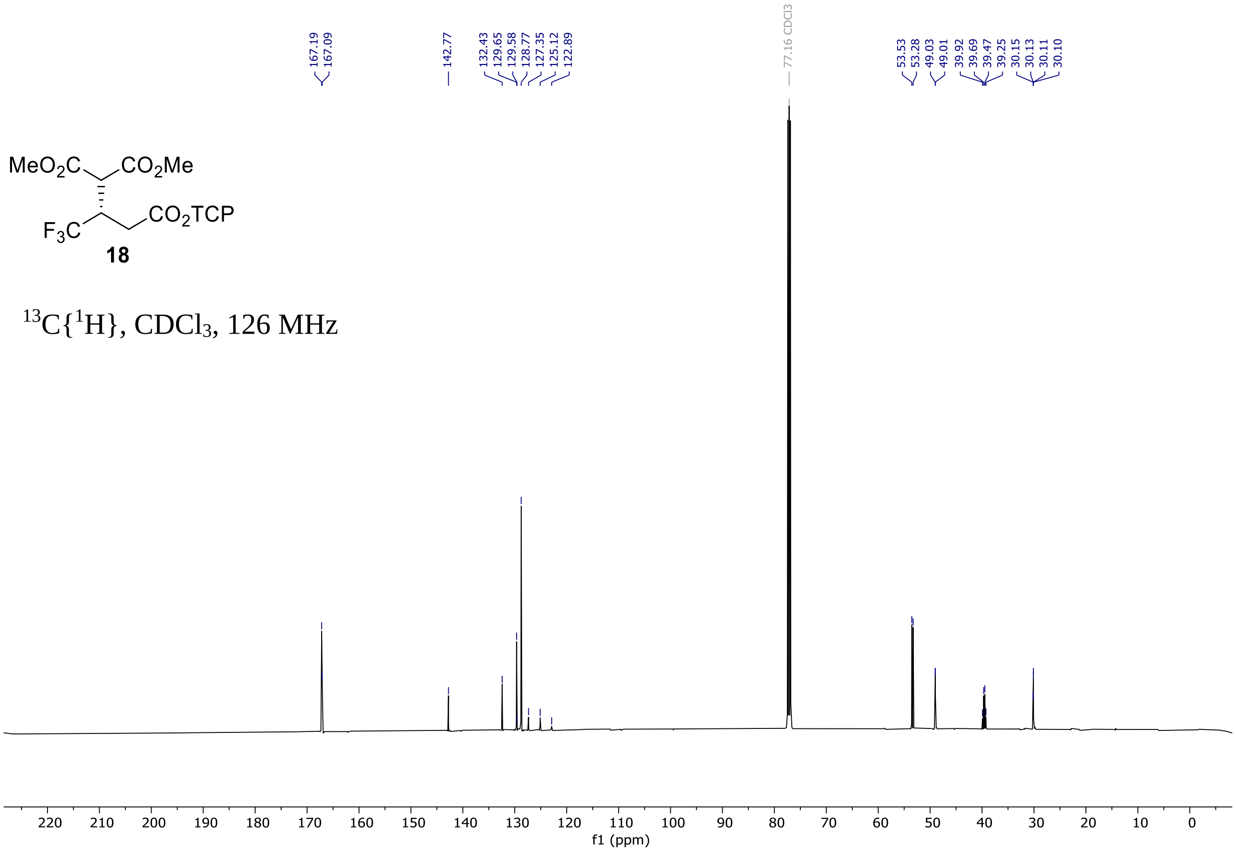
MeO2C CO2Me
F3C CO2TCP
18
13C{1H}, CDCl3, 126 MHz
167.19 167.09
142.77
132.43 129.65 129.58 128.77 127.35 125.12 122.89
77.16 CDCl3
53.53 53.28 49.03 49.01 39.92 39.69 39.47 39.25 30.15 30.13 30.11 30.10
f1 (ppm)

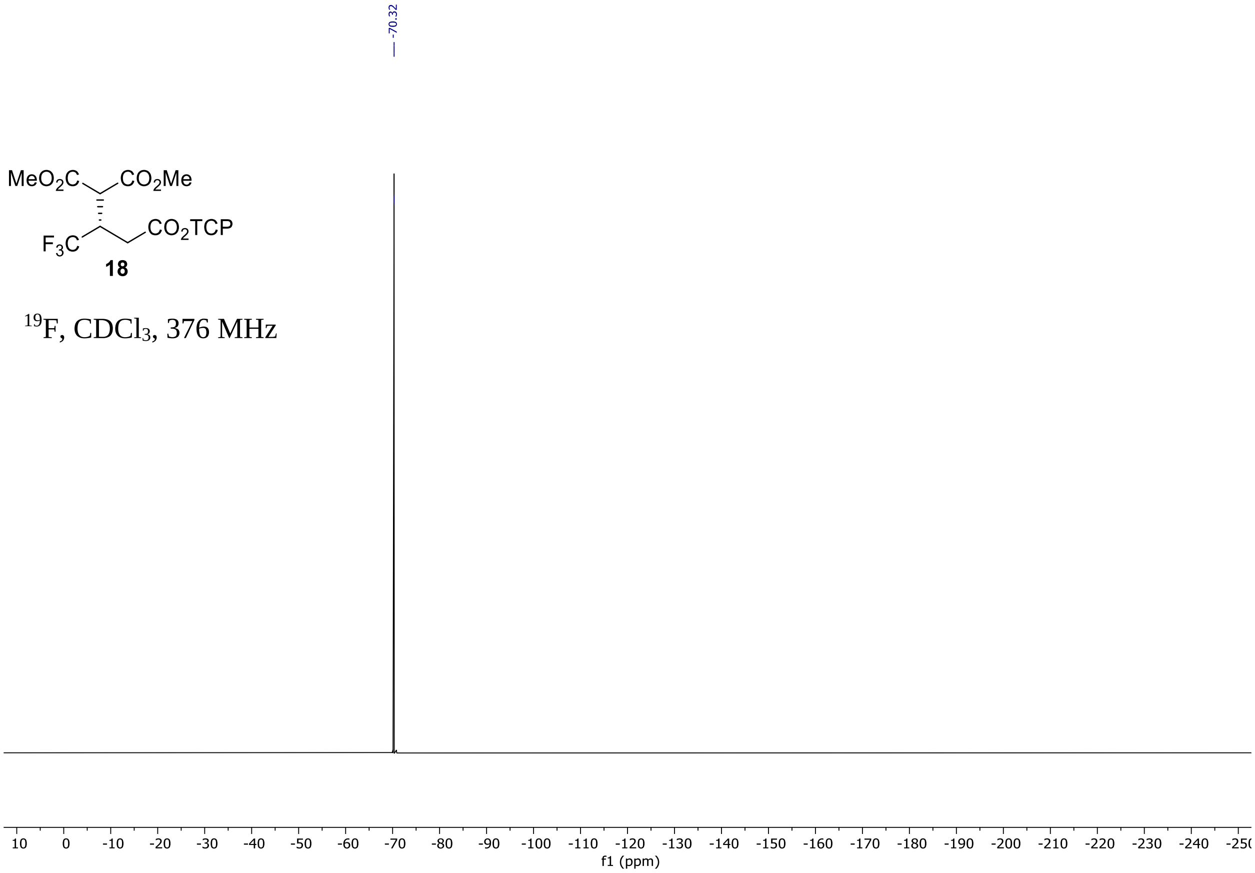

-70.32
MeO2C
CO2Me
CO2TCP
F3C
18
19F, CDCl3, 376 MHz
10 0 -10 -20 -30 -40 -50 -60 -70 -80 -90 -100 -110 -120 -130 -140 -150 -160 -170 -180 -190 -200 -210 -220 -230 -240
f1 (ppm)

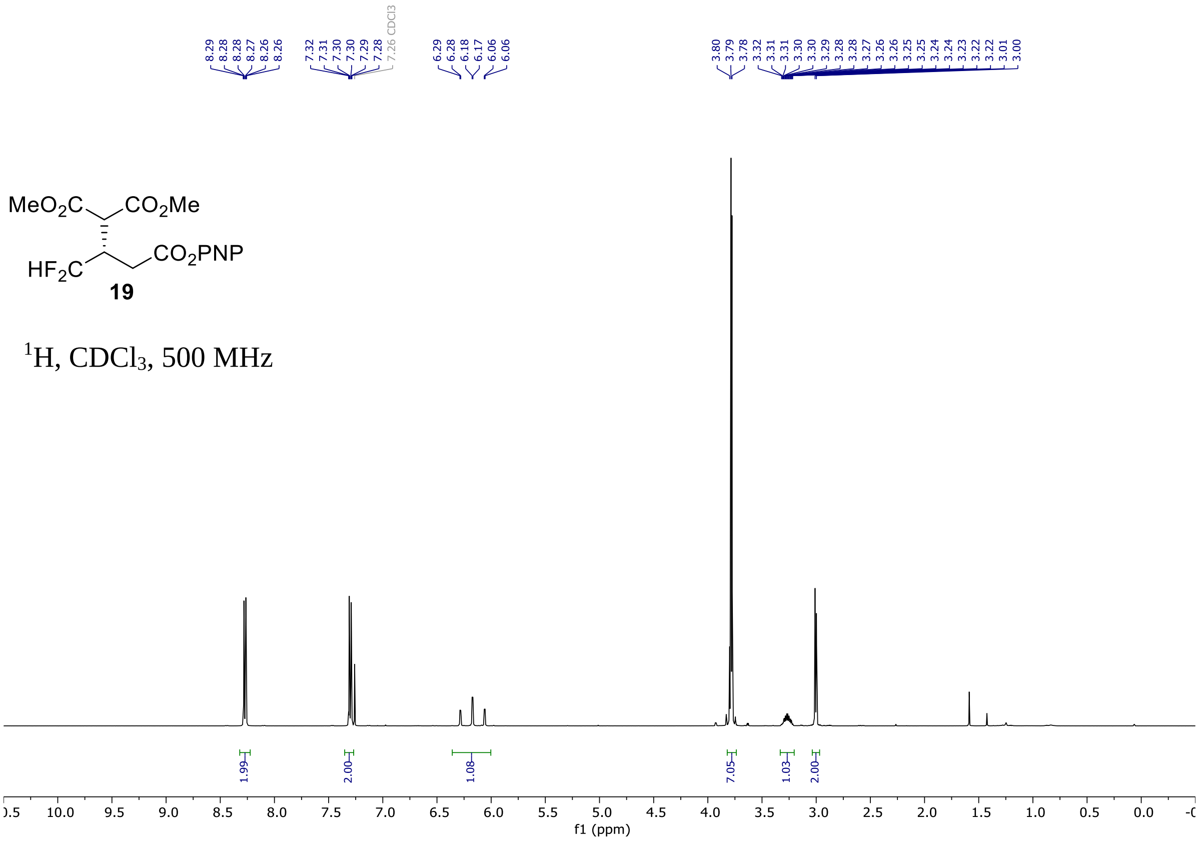

MeO2C CO2Me
HF2C CO2PNP
19
1H, CDCl3, 500 MHz
8.29 8.28 8.28 8.27 8.26 8.26
7.32 7.31 7.30 7.30 7.29 7.28
7.26 CDCl3
6.29 6.28 6.18 6.17 6.06 6.06
3.80 3.79 3.78 3.32 3.31 3.31 3.30 3.30 3.29 3.28 3.28 3.27 3.26 3.26 3.25 3.25 3.24 3.24 3.23 3.22 3.22 3.01 3.00
1.99 2.00 1.08 7.05 1.03 2.00
f1 (ppm)

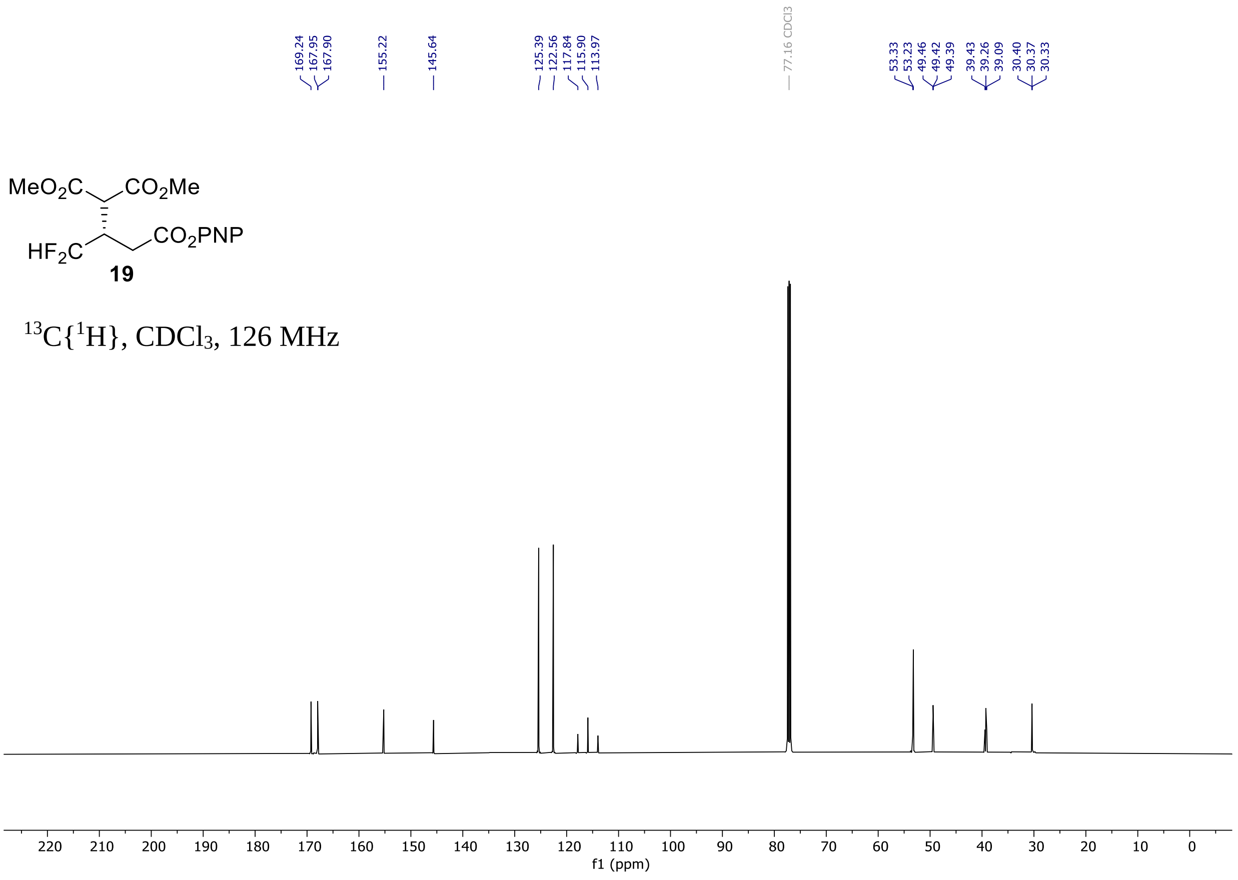

MeO2C CO2Me
HF2C CO2PNP
19
13C{1H}, CDCl3, 126 MHz
169.24 167.95 167.90
155.22
145.64
125.39 122.56 117.84 115.90 113.97
77.16 CDCl3
53.33 53.23 49.46 49.42 49.39
39.43 39.26 39.09
30.40 30.37 30.33
f1 (ppm)

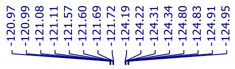

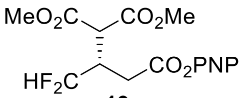

19

^{19}F, $CDCl_3$, 471 MHz

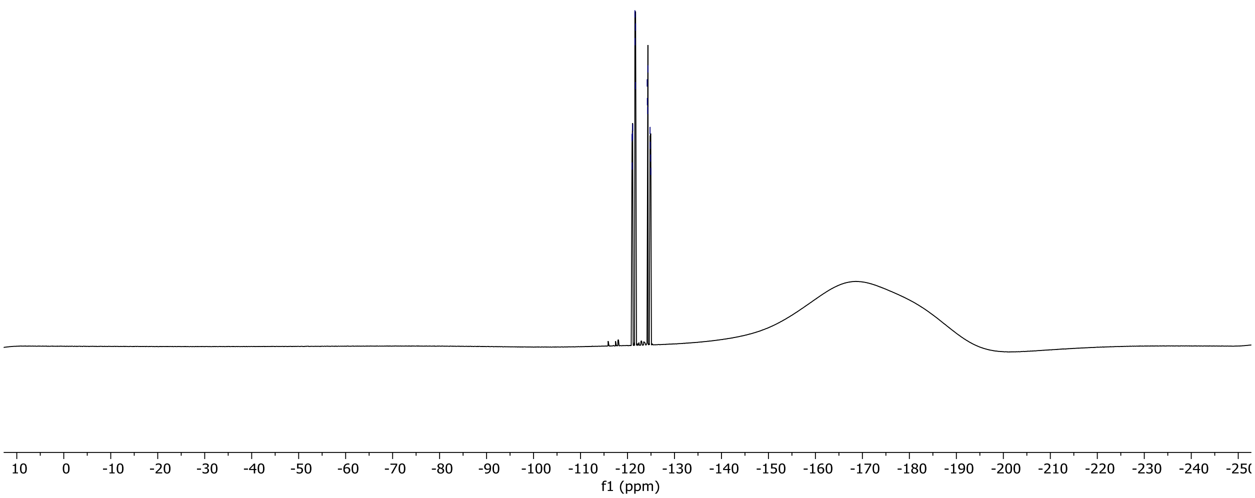

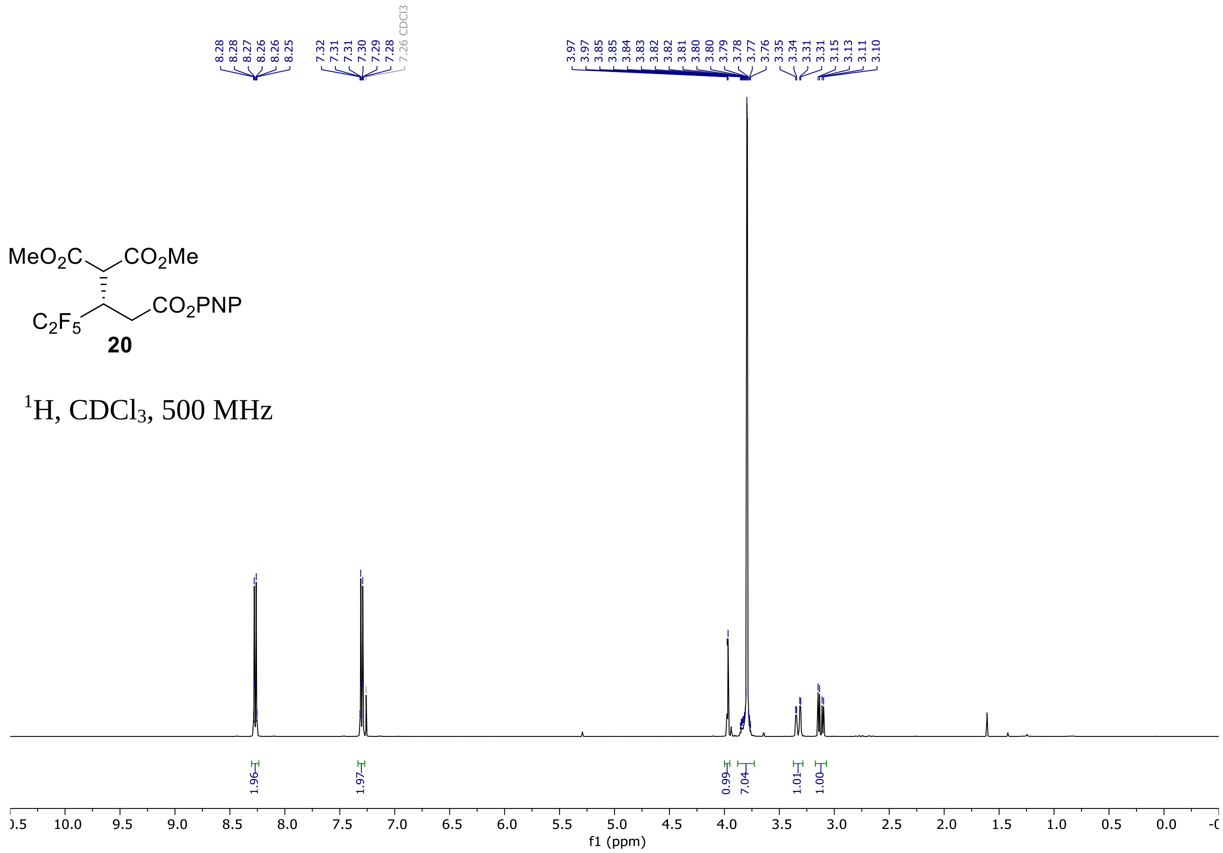
8.28 8.28 8.27 8.26 8.26 8.25
7.32 7.31 7.31 7.30 7.29 7.28
7.26 CDCl3
3.97 3.97 3.85 3.85 3.84 3.83 3.82 3.82 3.81 3.80 3.80 3.79 3.78 3.77 3.76
3.35 3.34 3.31 3.31 3.15 3.13 3.11 3.10
MeO_2C
CO_2Me
C_2F_5
CO_2PNP
20
^{1}H, $CDCl_3$, 500 MHz
1.96
1.97
0.99
7.04
1.01
1.00
f1 (ppm)

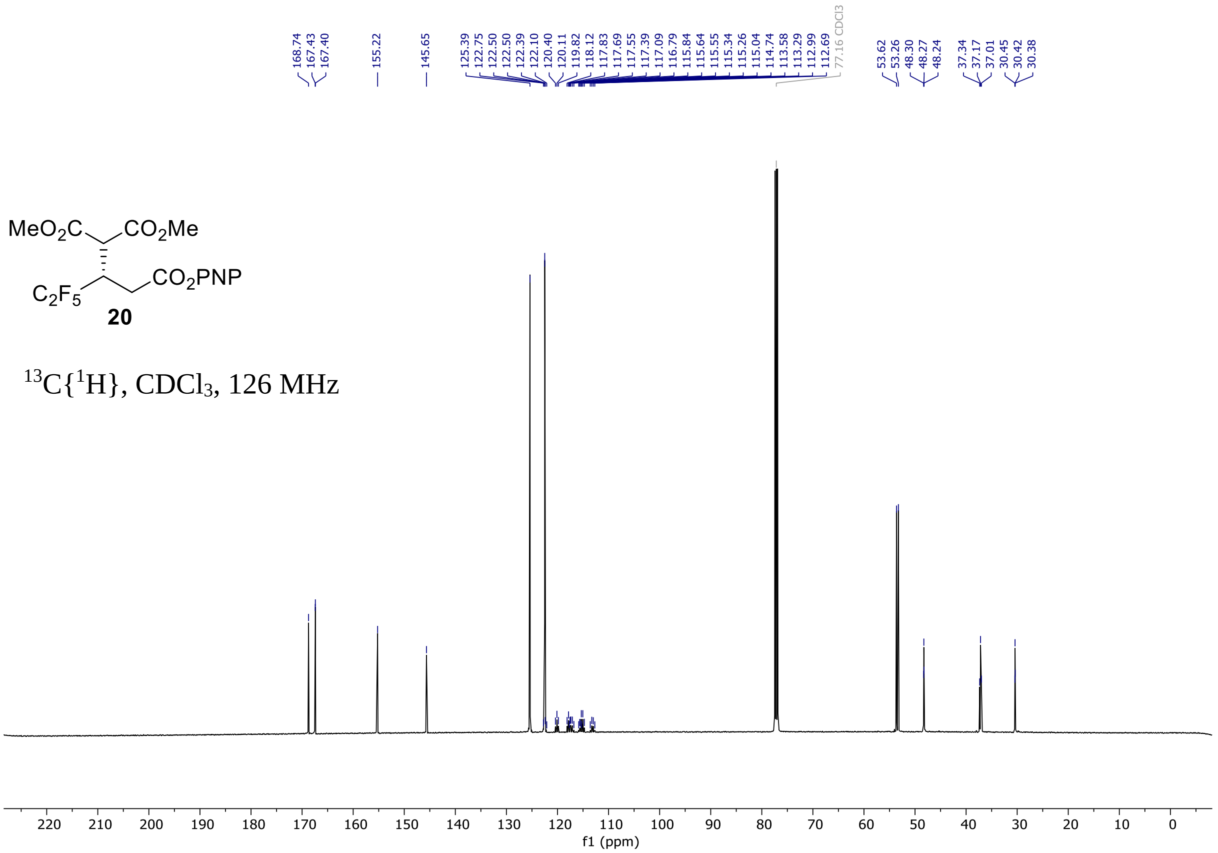
168.74 167.43 167.40
155.22
145.65
125.39 122.75 122.50 122.50 122.39 122.10 120.40 120.11 119.82 118.12 117.83 117.69 117.55 117.39 117.09 116.79 115.84 115.64 115.55 115.34 115.26 115.04 114.74 113.58 113.29 112.99 112.69
77.16 CDCl3
53.62 53.26 48.30 48.27 48.24
37.34 37.17 37.01 30.45 30.42 30.38
MeO_2C
CO_2Me
C_2F_5
CO_2PNP
20
$^{13}C\{^{1}H\}$, $CDCl_3$, 126 MHz
f1 (ppm)

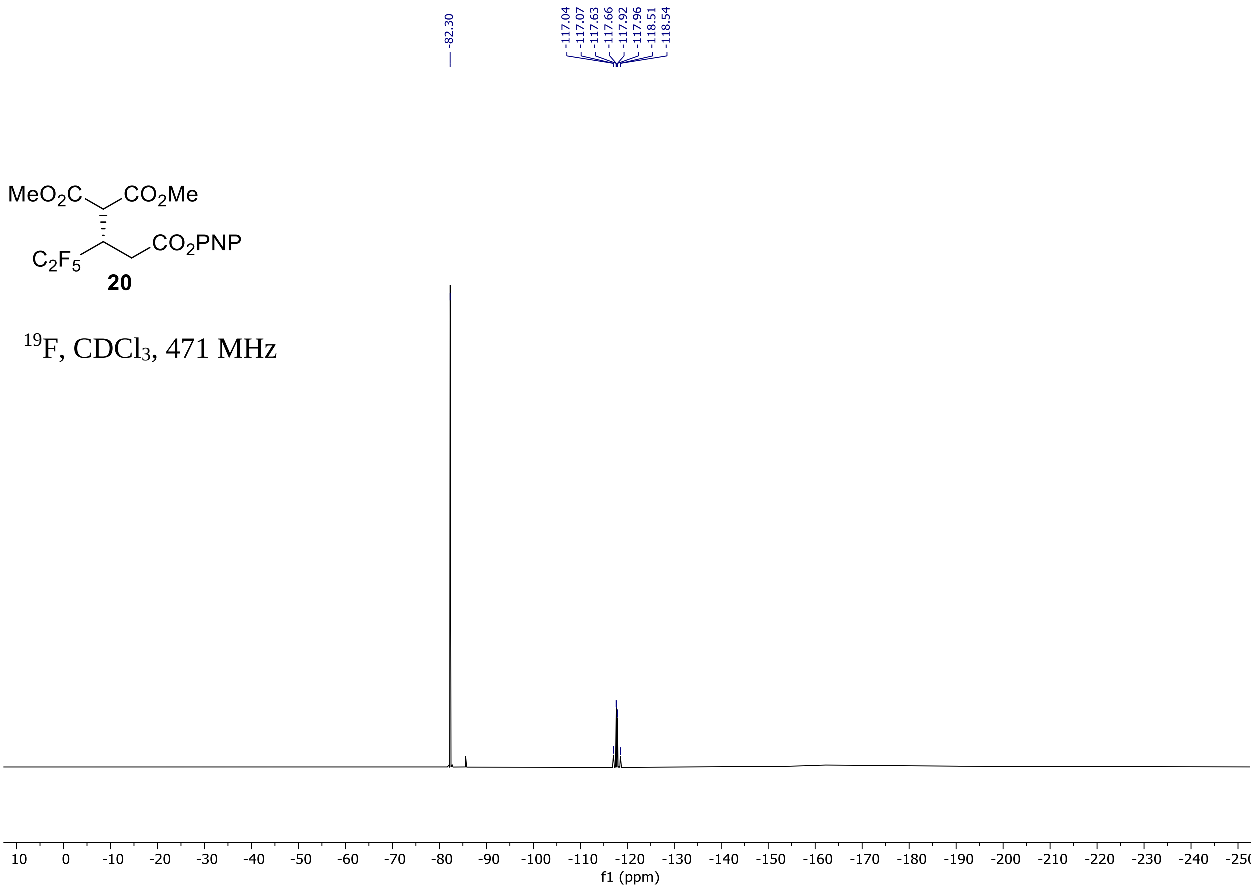

^{19}F, $CDCl_3$, 471 MHz

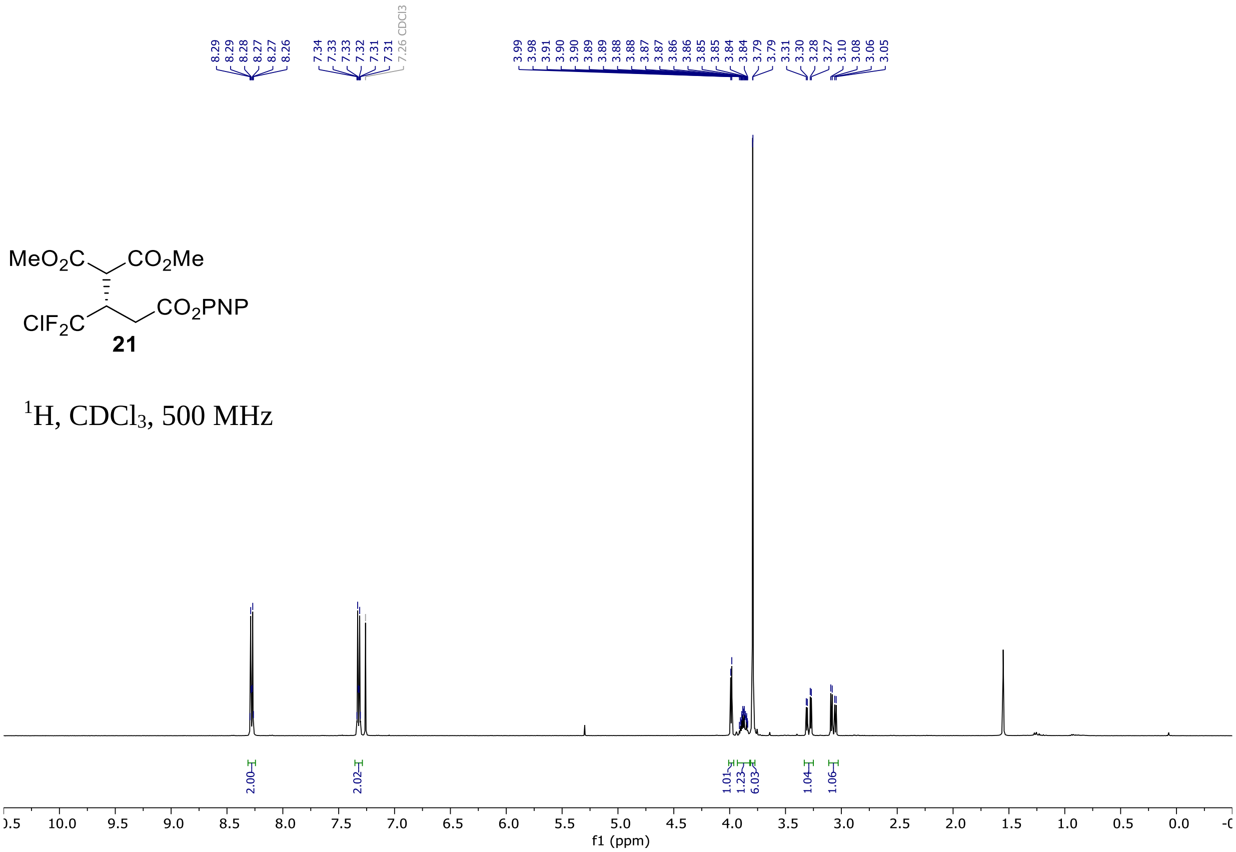
8.29
8.29
8.28
8.27
8.27
8.26
7.34
7.33
7.33
7.32
7.31
7.31
7.26 CDCl3
3.99
3.98
3.91
3.90
3.90
3.89
3.89
3.88
3.88
3.87
3.87
3.86
3.86
3.85
3.85
3.84
3.84
3.79
3.79
3.31
3.30
3.28
3.27
3.10
3.08
3.06
3.05
MeO2C
CO2Me
ClF2C
CO2PNP
21
1H, CDCl3, 500 MHz
2.00
2.02
1.01
1.23
6.03
1.04
1.06
f1 (ppm)

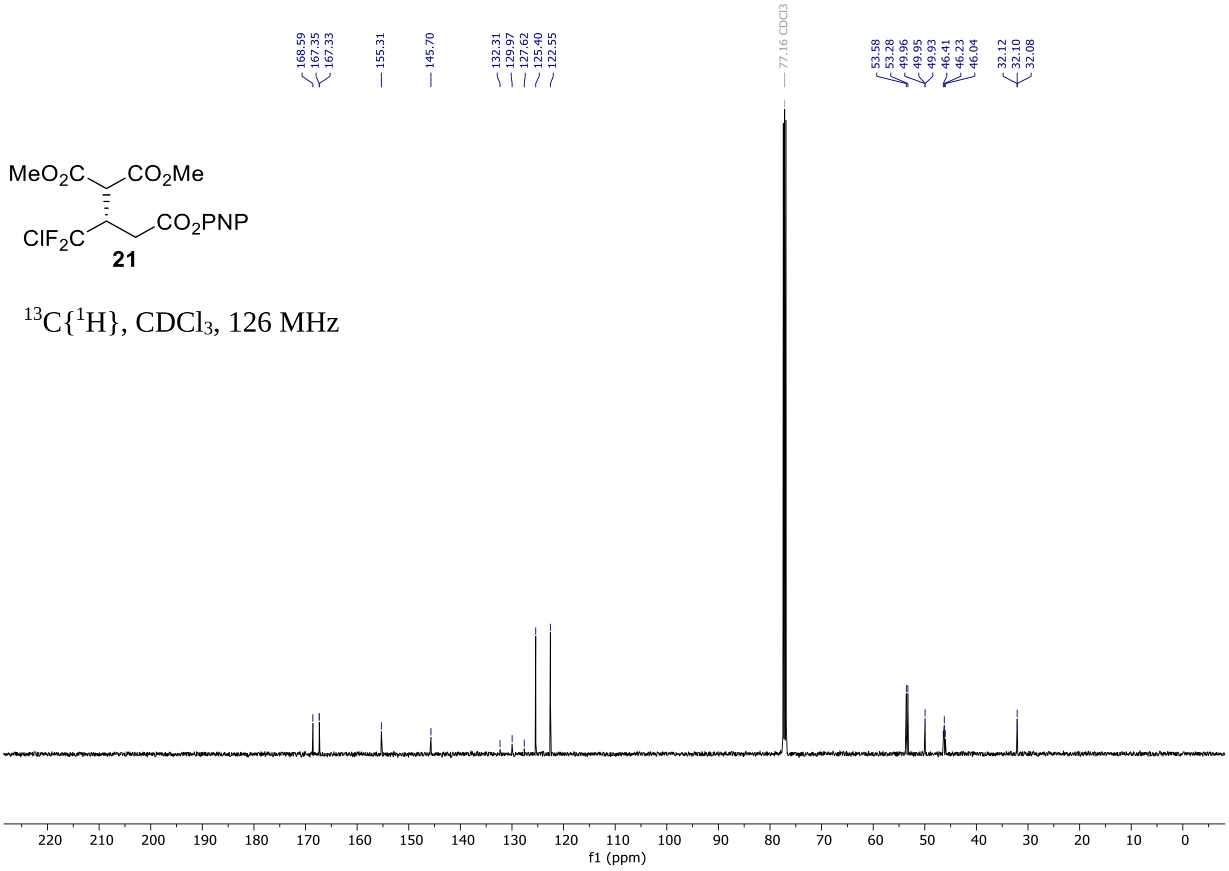
168.59
167.35
167.33
155.31
145.70
132.31
129.97
127.62
125.40
122.55
77.16 CDCl3
53.58
53.28
49.96
49.95
49.93
46.41
46.23
46.04
32.12
32.10
32.08
MeO2C
CO2Me
ClF2C
CO2PNP
21
13C{1H}, CDCl3, 126 MHz
f1 (ppm)

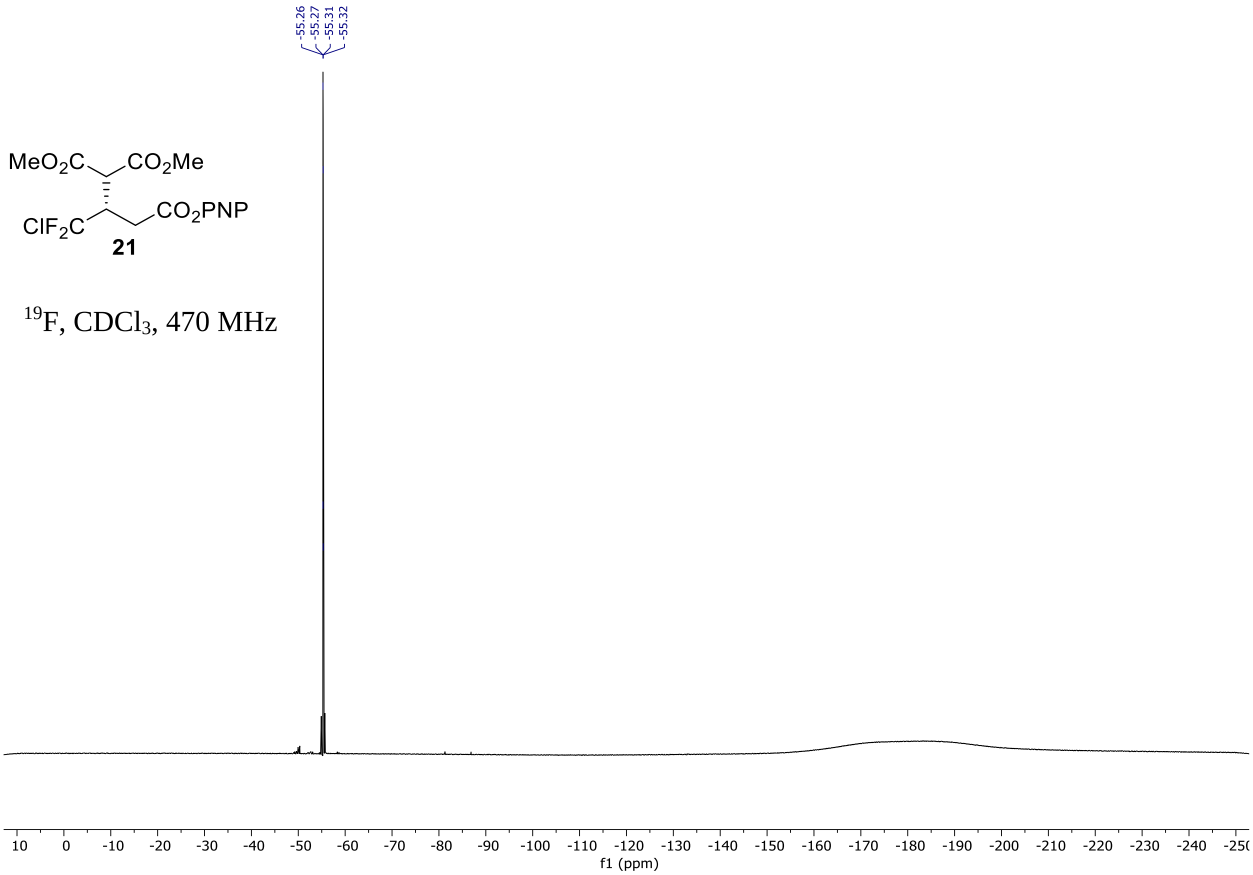

^{19}F, $CDCl_3$, 470 MHz

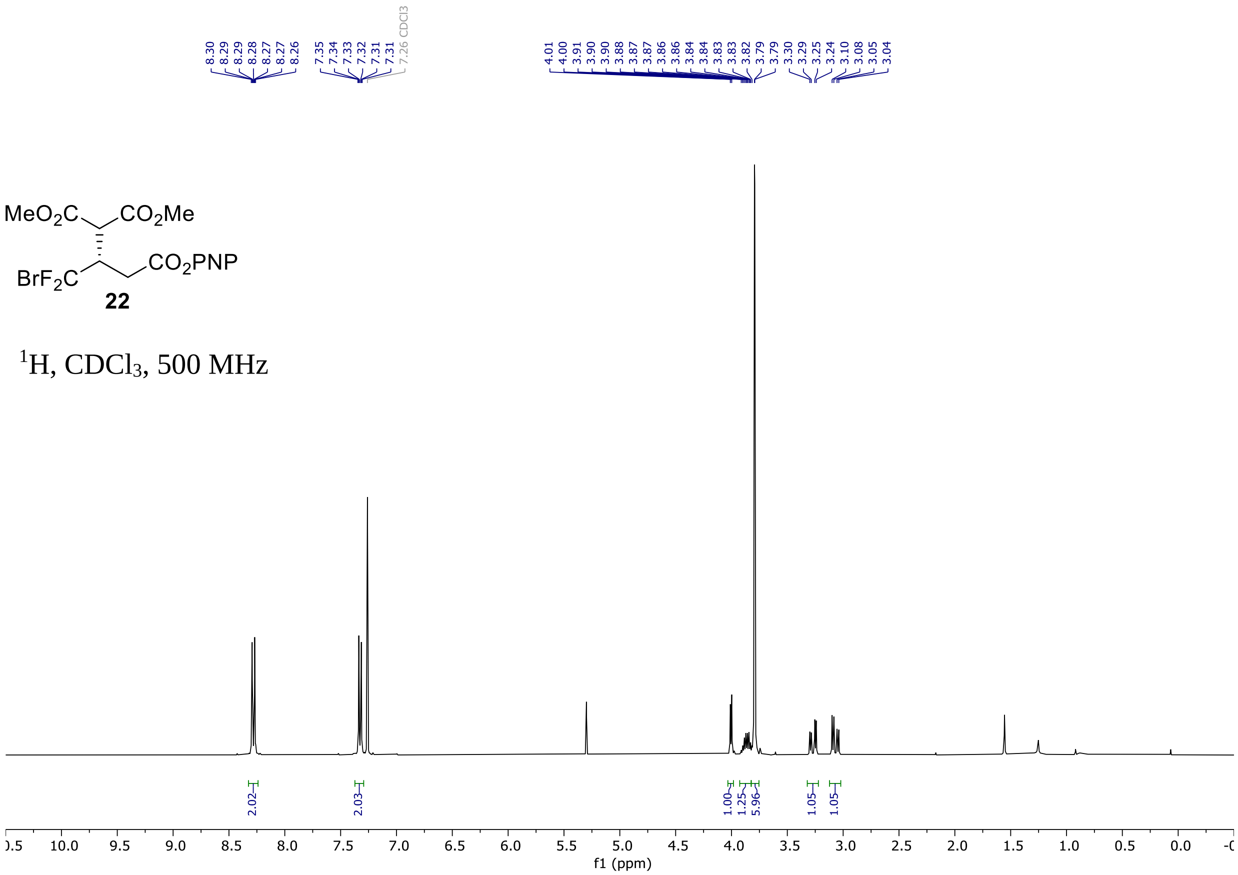
8.30 8.29 8.29 8.28 8.27 8.27 8.26
7.35 7.34 7.33 7.32 7.31 7.31 7.26 CDCl3
4.01 4.00 3.91 3.90 3.90 3.88 3.87 3.87 3.86 3.86 3.84 3.84 3.83 3.83 3.82 3.79 3.79 3.30 3.29 3.25 3.24 3.10 3.08 3.05 3.04
MeO2C CO2Me
BrF2C CO2PNP
22
1H, CDCl3, 500 MHz
2.02 2.03 1.00 1.25 5.96 1.05 1.05
f1 (ppm)

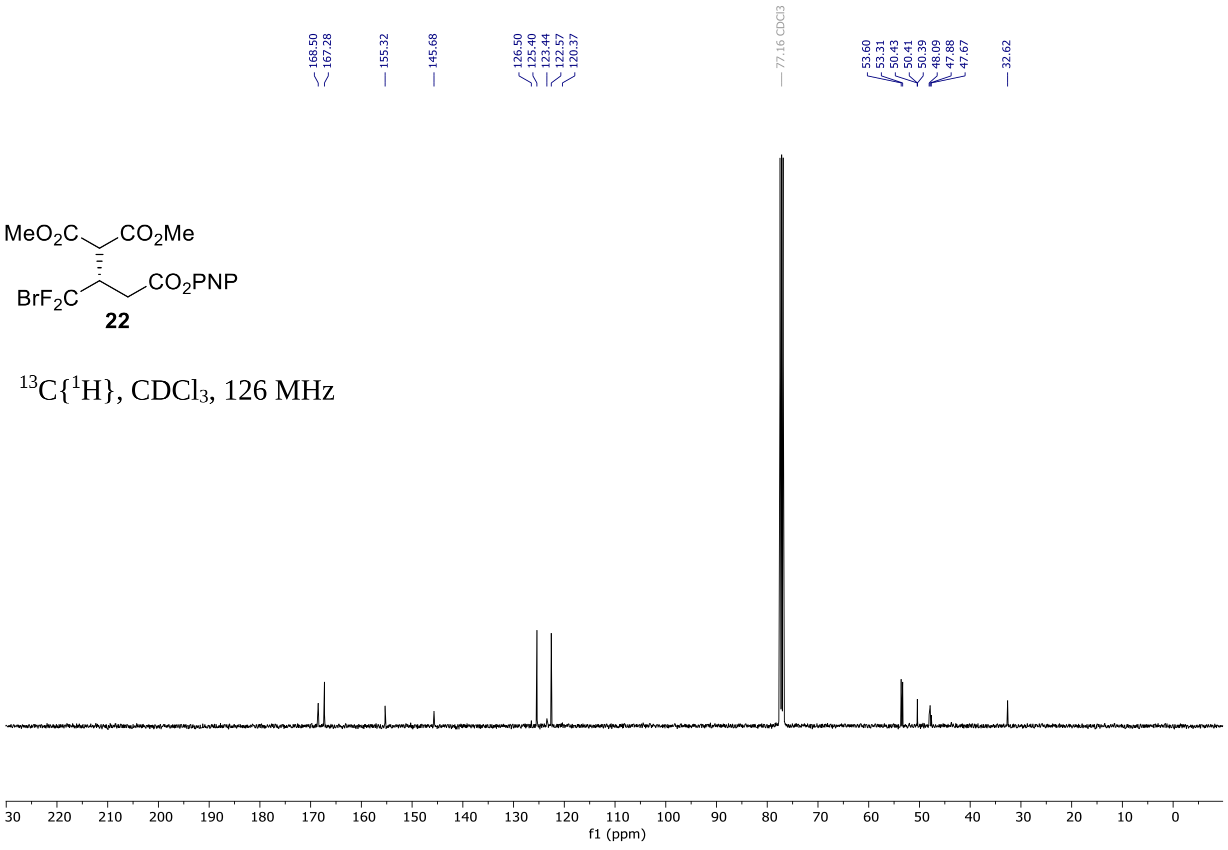
168.50 167.28 155.32 145.68 126.50 125.40 123.44 122.57 120.37 77.16 CDCl3 53.60 53.31 50.43 50.41 50.39 48.09 47.88 47.67 32.62
MeO2C CO2Me
BrF2C CO2PNP
22
13C{1H}, CDCl3, 126 MHz
f1 (ppm)

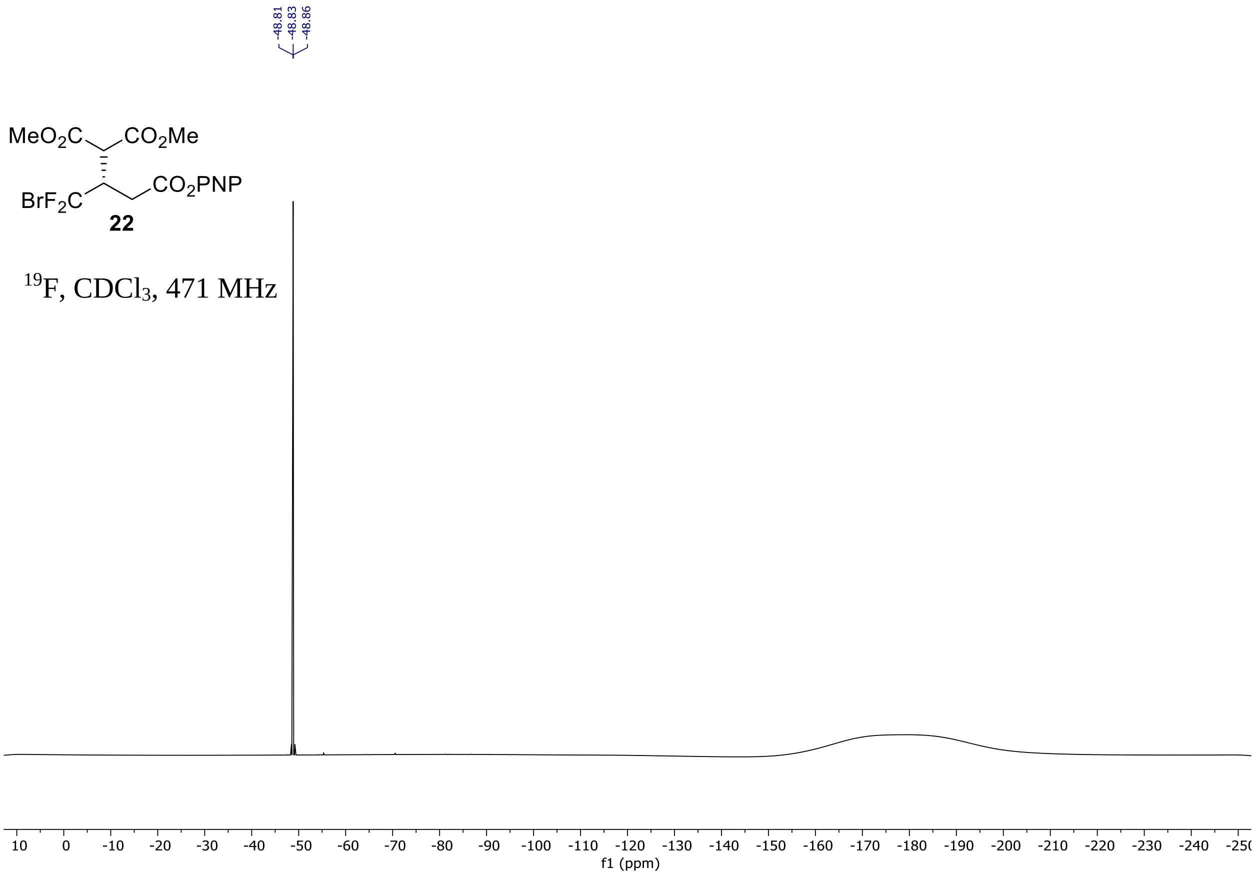
-48.81
-48.83
-48.86
MeO2C
CO2Me
BrF2C
CO2PNP
22
19F, CDCl3, 471 MHz
10 0 -10 -20 -30 -40 -50 -60 -70 -80 -90 -100 -110 -120 -130 -140 -150 -160 -170 -180 -190 -200 -210 -220 -230 -240 -250
f1 (ppm)

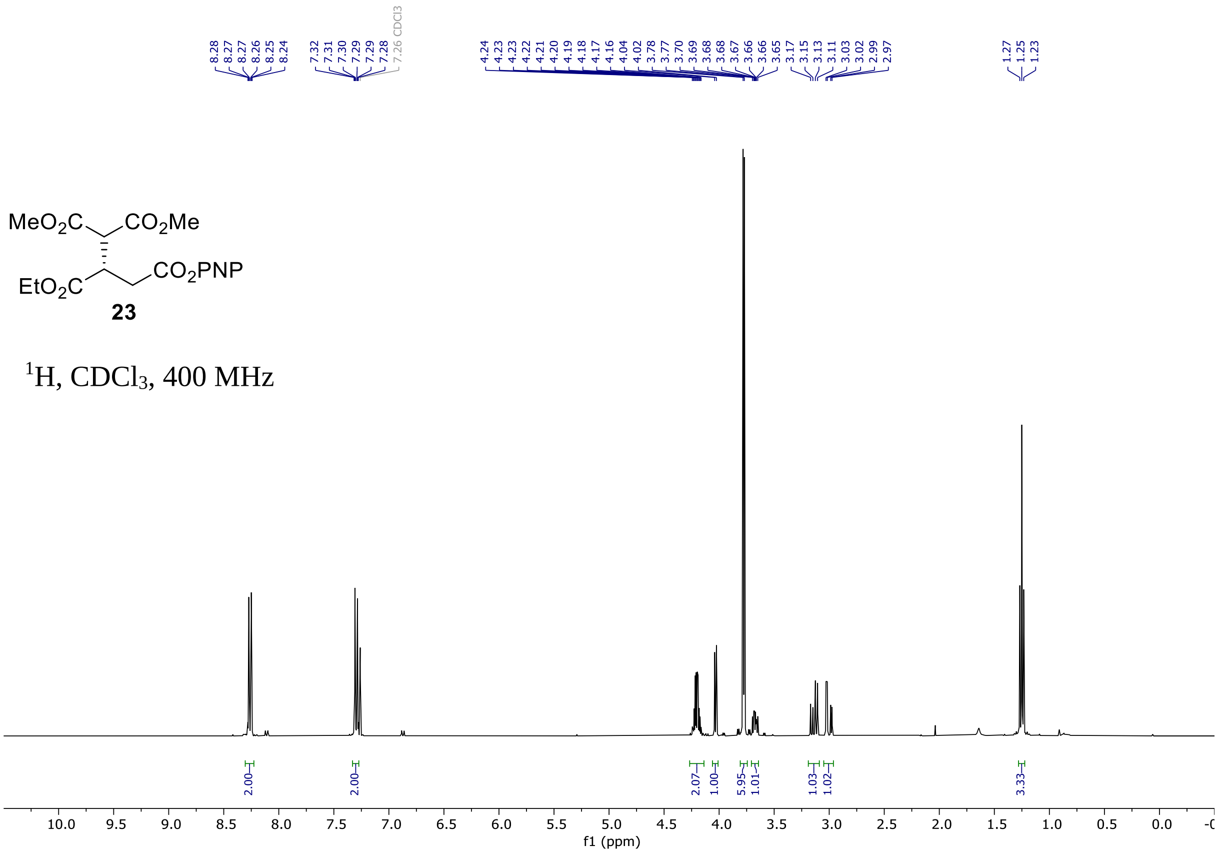

MeO2C
CO2Me
CO2PNP
EtO2C
23
1H, CDCl3, 400 MHz
8.28
8.27
8.27
8.26
8.25
8.24
7.32
7.31
7.30
7.29
7.29
7.28
7.26 CDCl3
4.24
4.23
4.23
4.22
4.21
4.20
4.19
4.18
4.17
4.16
4.04
4.02
3.78
3.77
3.70
3.69
3.68
3.67
3.66
3.65
3.17
3.15
3.13
3.11
3.03
3.02
2.99
2.97
1.27
1.25
1.23
2.00
2.00
2.07
1.00
5.95
1.01
1.03
1.02
3.33
f1 (ppm)

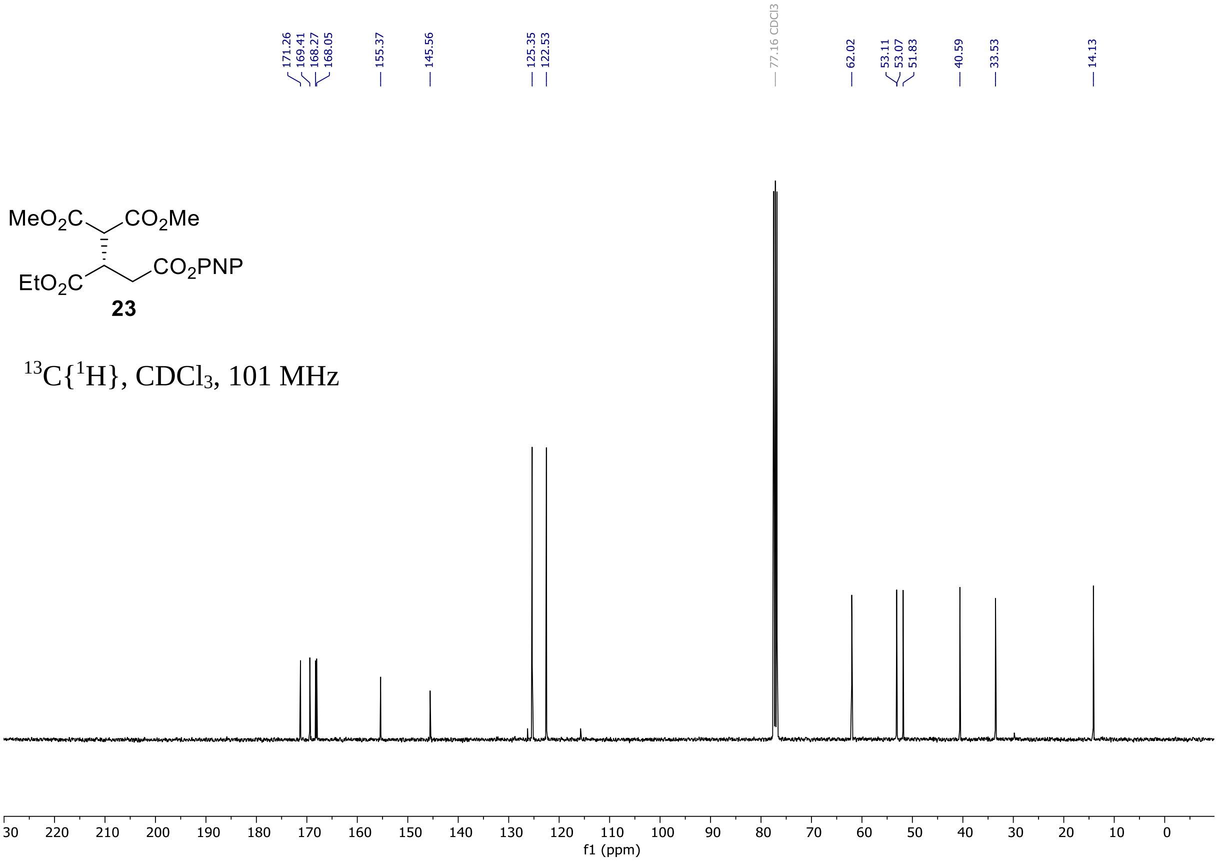

171.26
169.41
168.27
168.05
155.37
145.56
125.35
122.53
77.16 CDCl3
62.02
53.11
53.07
51.83
40.59
33.53
14.13
MeO2C
CO2Me
CO2PNP
EtO2C
23
13C{1H}, CDCl3, 101 MHz
f1 (ppm)

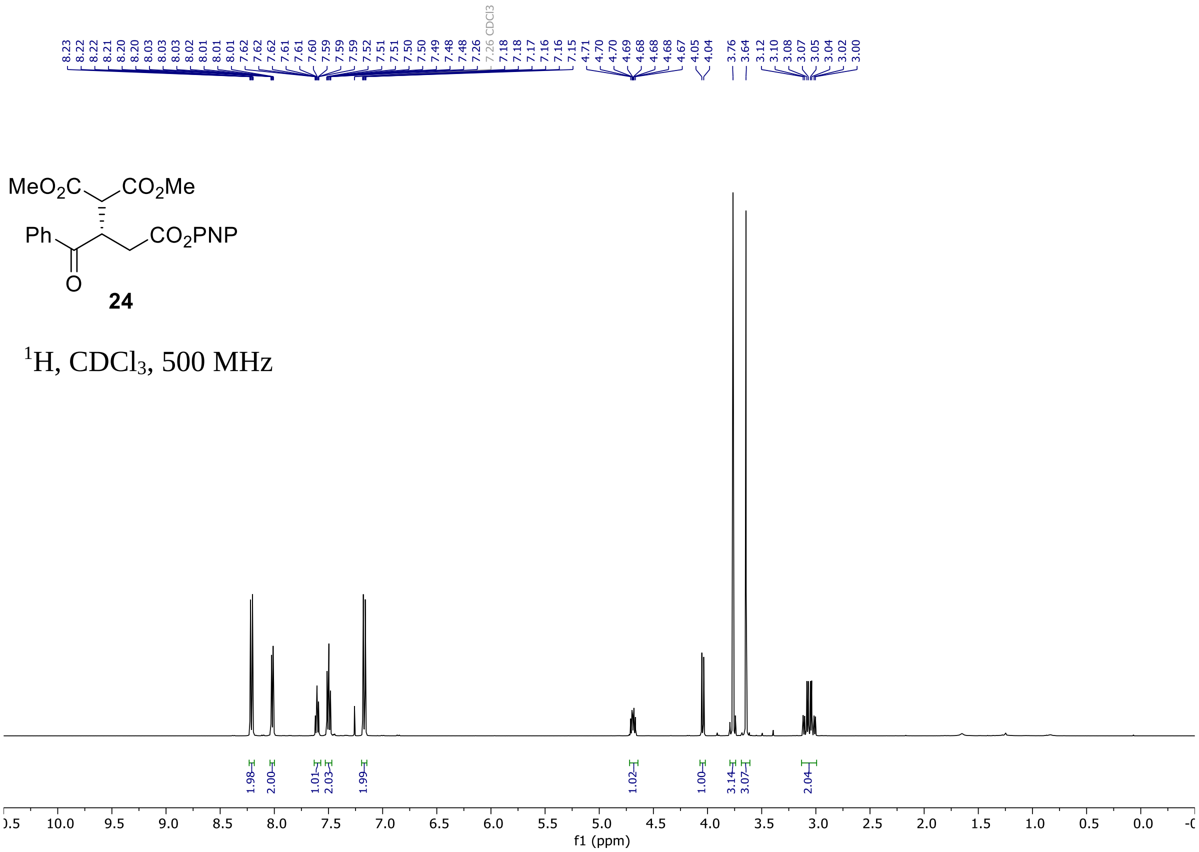

8.23 8.22 8.22 8.21 8.20 8.20 8.03 8.03 8.03 8.02 8.02 8.01 8.01 7.62 7.62 7.62 7.61 7.61 7.60 7.59 7.59 7.59 7.52 7.51 7.51 7.50 7.49 7.48 7.48 7.26 7.26 CDCl3 7.18 7.18 7.17 7.16 7.16 7.15 4.71 4.70 4.69 4.68 4.68 4.68 4.67 4.05 4.04 3.76 3.64 3.12 3.10 3.08 3.07 3.05 3.04 3.02 3.00
MeO2C
CO2Me
Ph
CO2PNP
O
24
1H, CDCl3, 500 MHz
1.98 2.00 1.01 2.03 1.99 1.02 1.00 3.14 3.07 2.04
f1 (ppm)

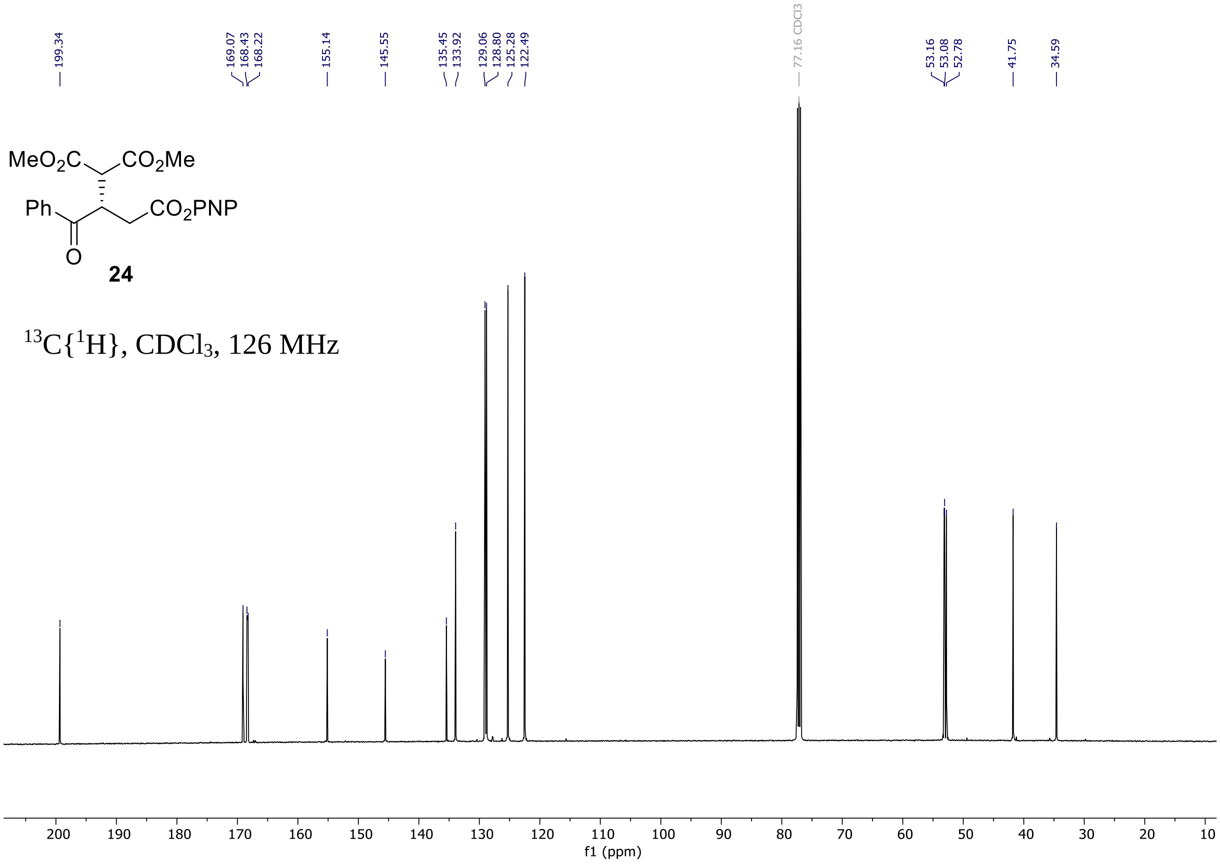

199.34 169.07 168.43 168.22 155.14 145.55 135.45 133.92 129.06 128.80 125.28 122.49 77.16 CDCl3 53.16 53.08 52.78 41.75 34.59
MeO2C
CO2Me
Ph
CO2PNP
O
24
13C{1H}, CDCl3, 126 MHz
f1 (ppm)

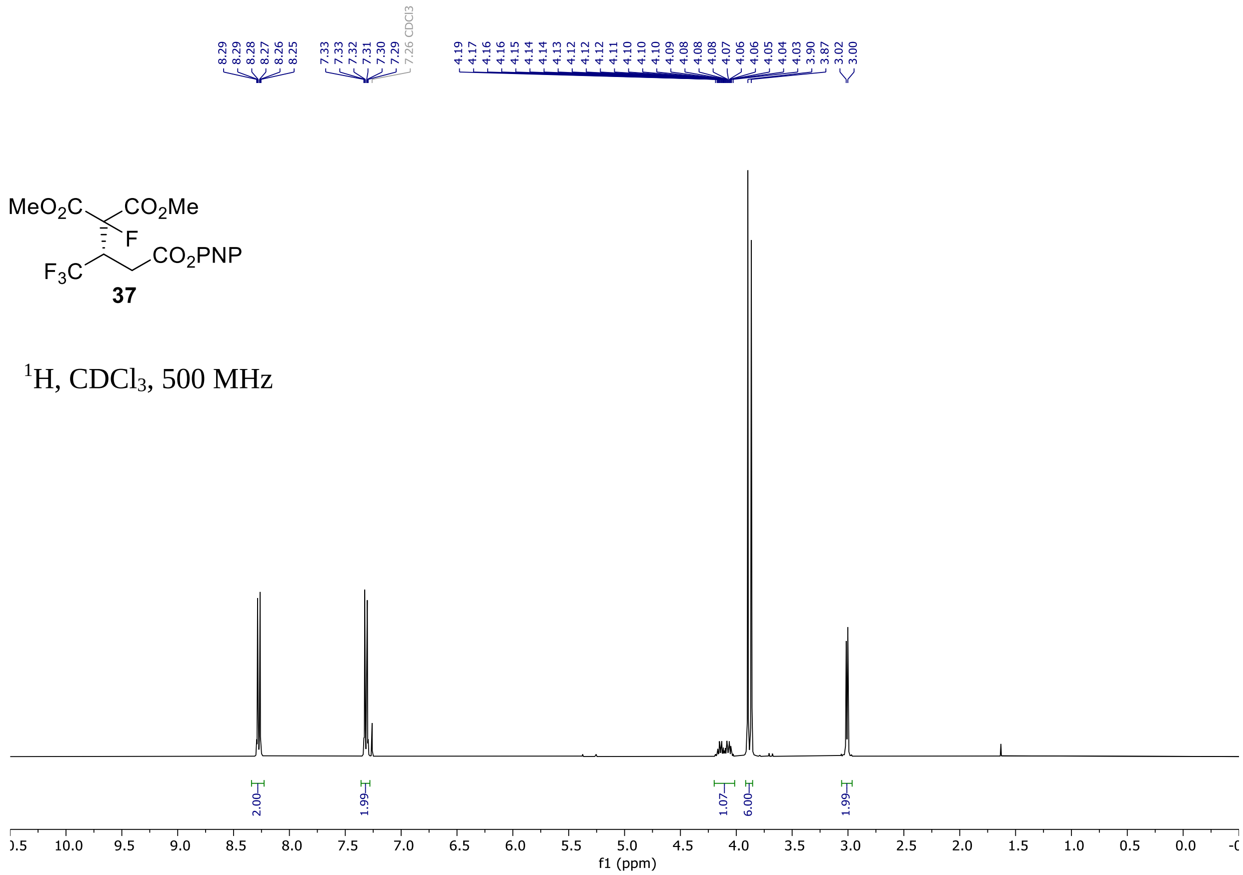
MeO2C CO2Me
F
CO2PNP
F3C
37
1H, CDCl3, 500 MHz
f1 (ppm)

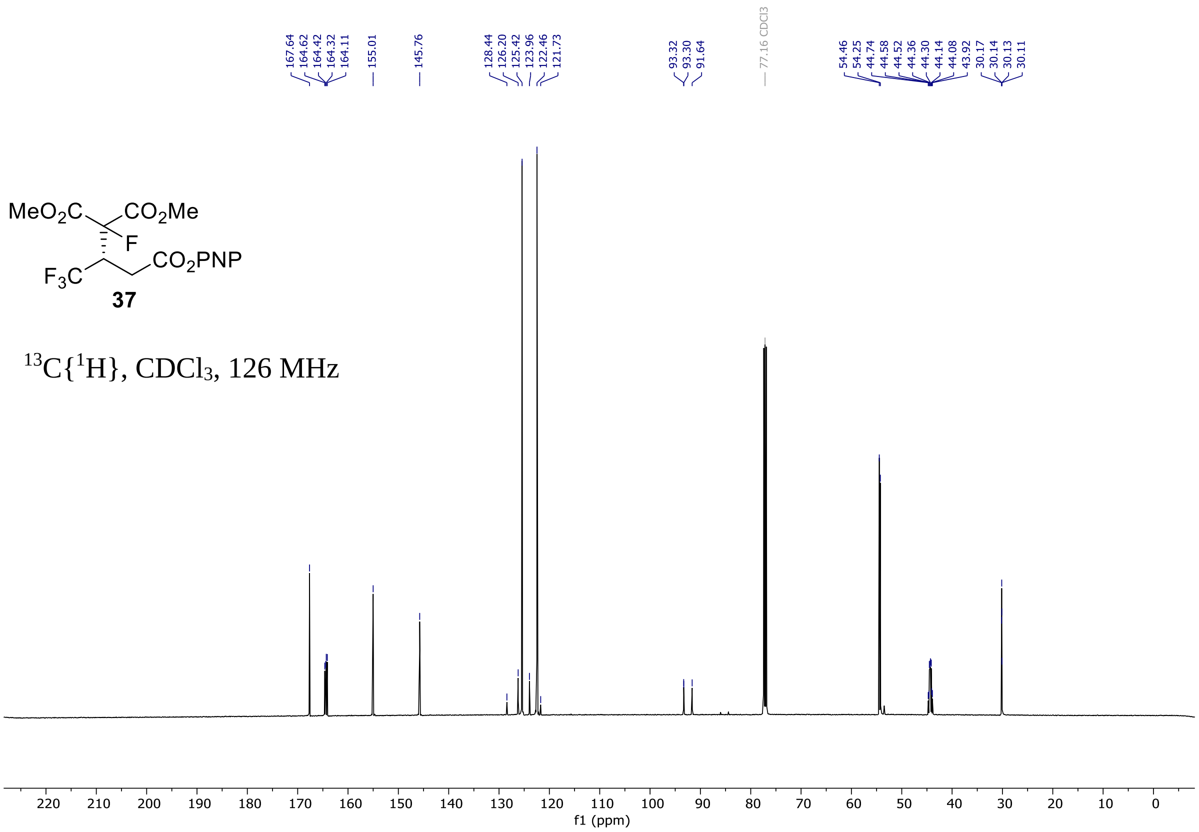
MeO2C CO2Me
F
CO2PNP
F3C
37
13C{1H}, CDCl3, 126 MHz
f1 (ppm)

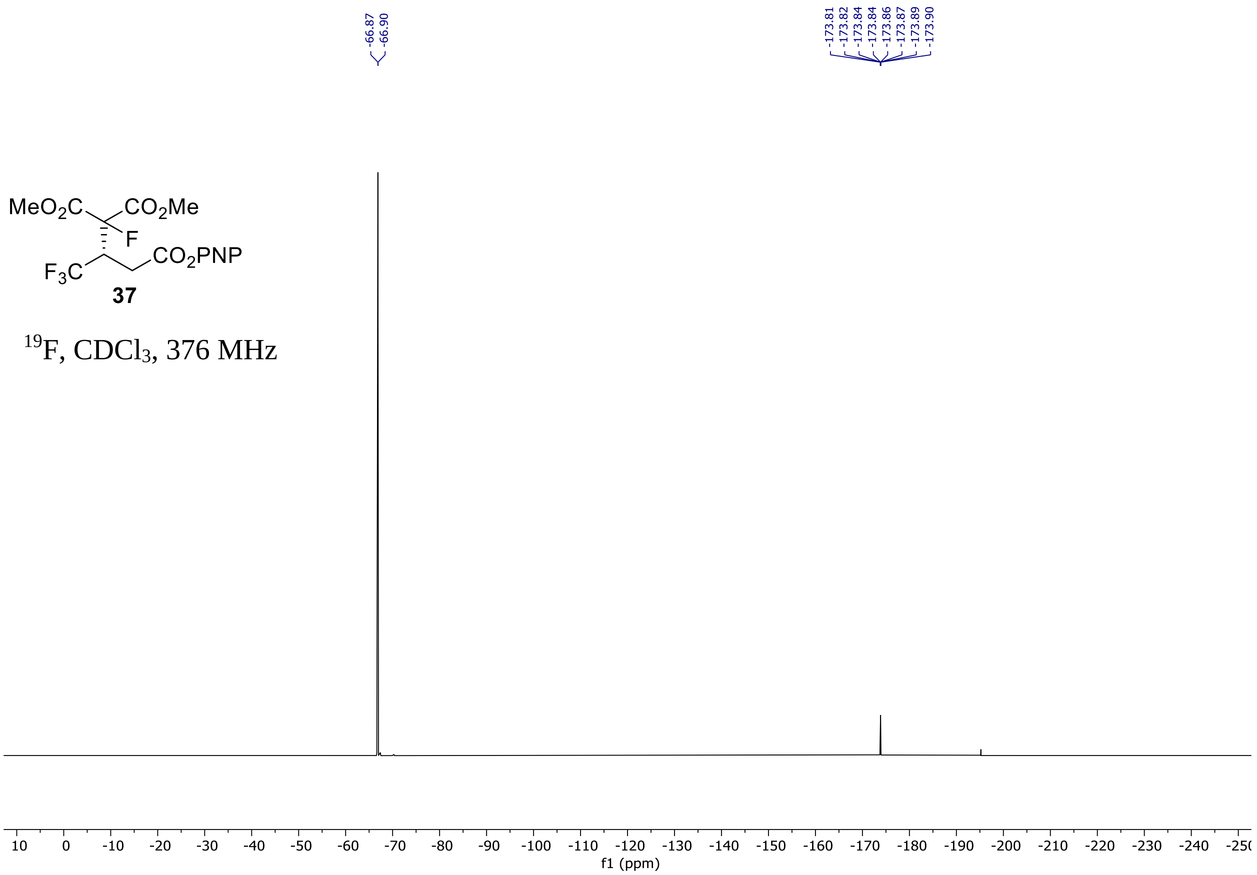
-66.87
-66.90
-173.81
-173.82
-173.84
-173.84
-173.86
-173.87
-173.89
-173.90
MeO2C
CO2Me
F
F3C
CO2PNP
37
19F, CDCl3, 376 MHz
10 0 -10 -20 -30 -40 -50 -60 -70 -80 -90 -100 -110 -120 -130 -140 -150 -160 -170 -180 -190 -200 -210 -220 -230 -240 -250
f1 (ppm)

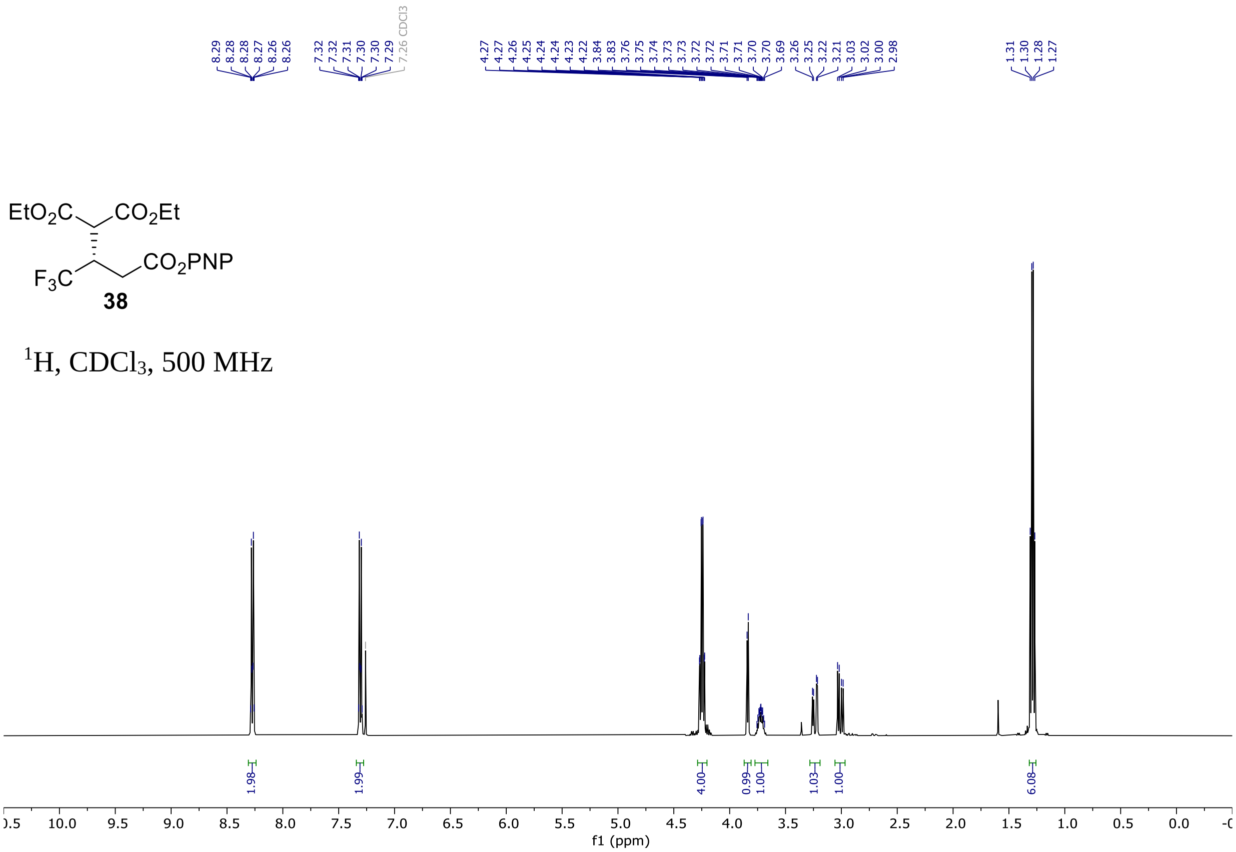
8.29 8.28 8.28 8.27 8.26 8.26
7.32 7.32 7.31 7.30 7.30 7.29
7.26 CDCl3
4.27 4.27 4.26 4.25 4.24 4.24 4.23 4.22 3.84 3.83 3.76 3.75 3.74 3.73 3.73 3.72 3.72 3.71 3.71 3.70 3.70 3.69 3.26 3.25 3.22 3.21 3.03 3.02 3.00 2.98
1.31 1.30 1.28 1.27
EtO2C CO2Et
F3C CO2PNP
38
1H, CDCl3, 500 MHz
1.98
1.99
4.00
0.99
1.00
1.03
1.00
6.08
f1 (ppm)

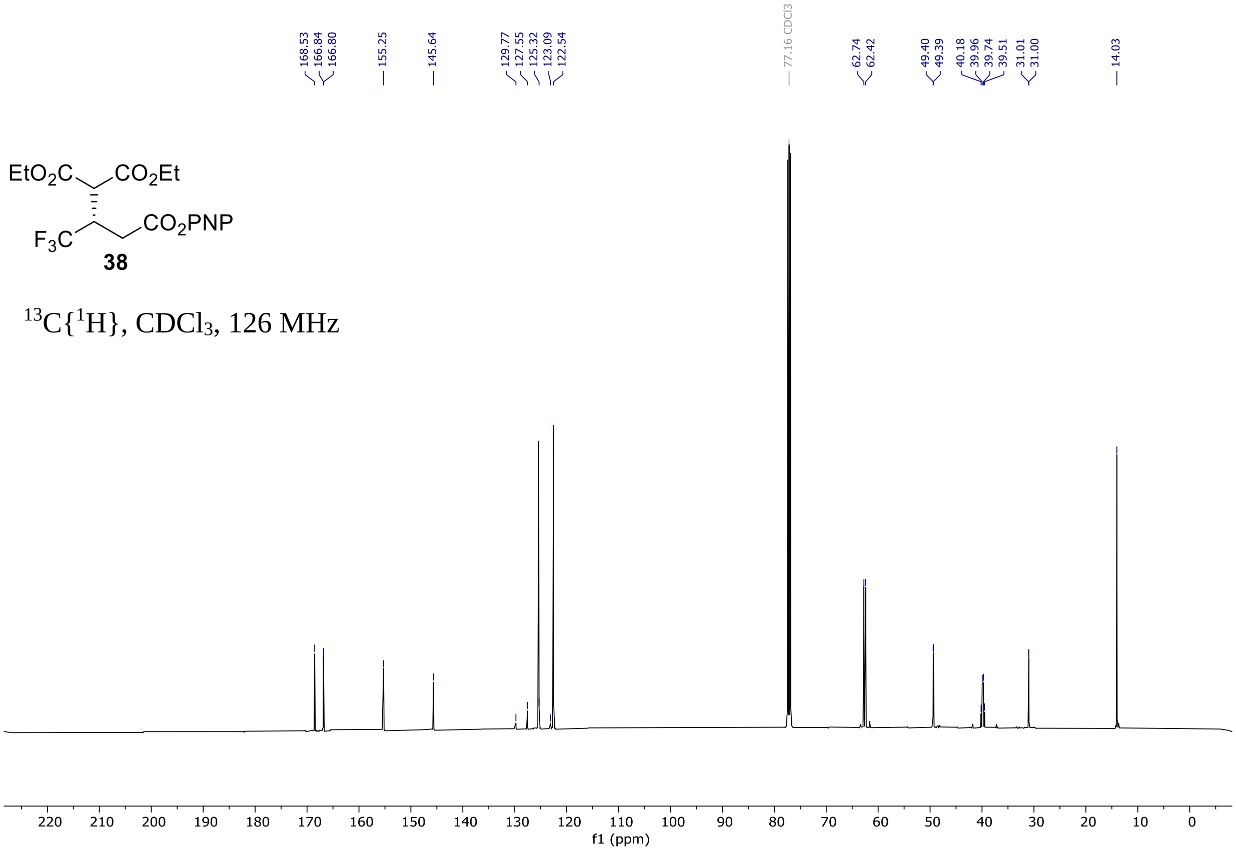
168.53 166.84 166.80
155.25
145.64
129.77 127.55 125.32 123.09 122.54
77.16 CDCl3
62.74 62.42
49.40 49.39
40.18 39.96 39.74 39.51
31.01 31.00
14.03
EtO2C CO2Et
F3C CO2PNP
38
13C{1H}, CDCl3, 126 MHz
f1 (ppm)

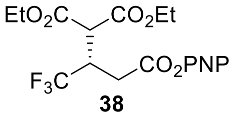

38

^{19}F, $CDCl_3$, 471 MHz

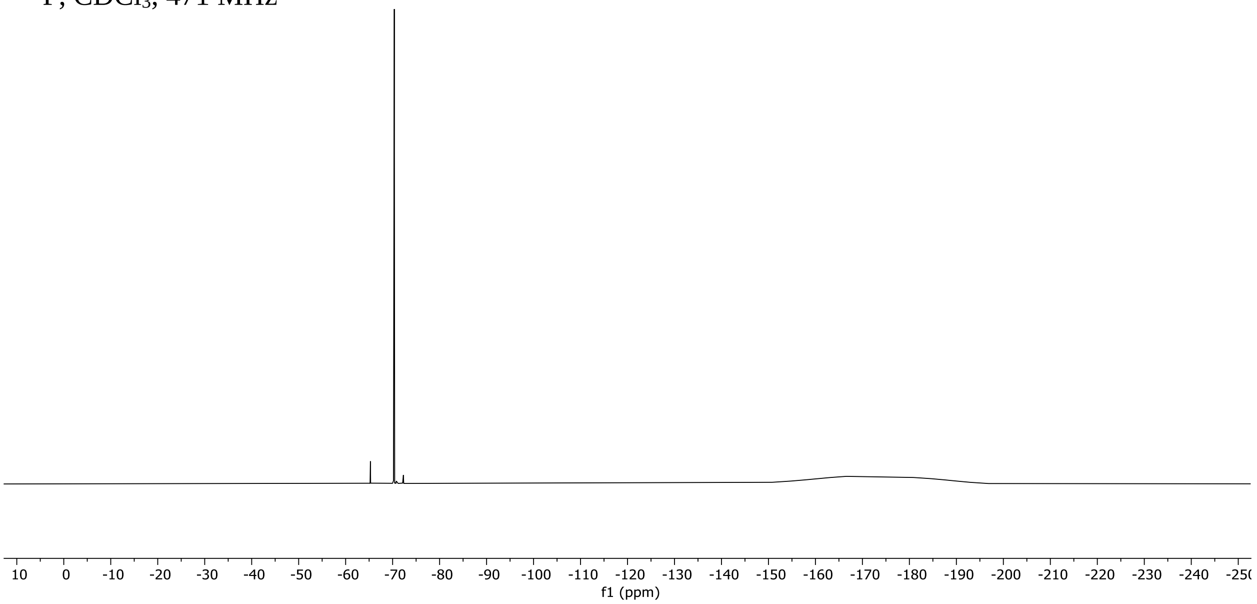

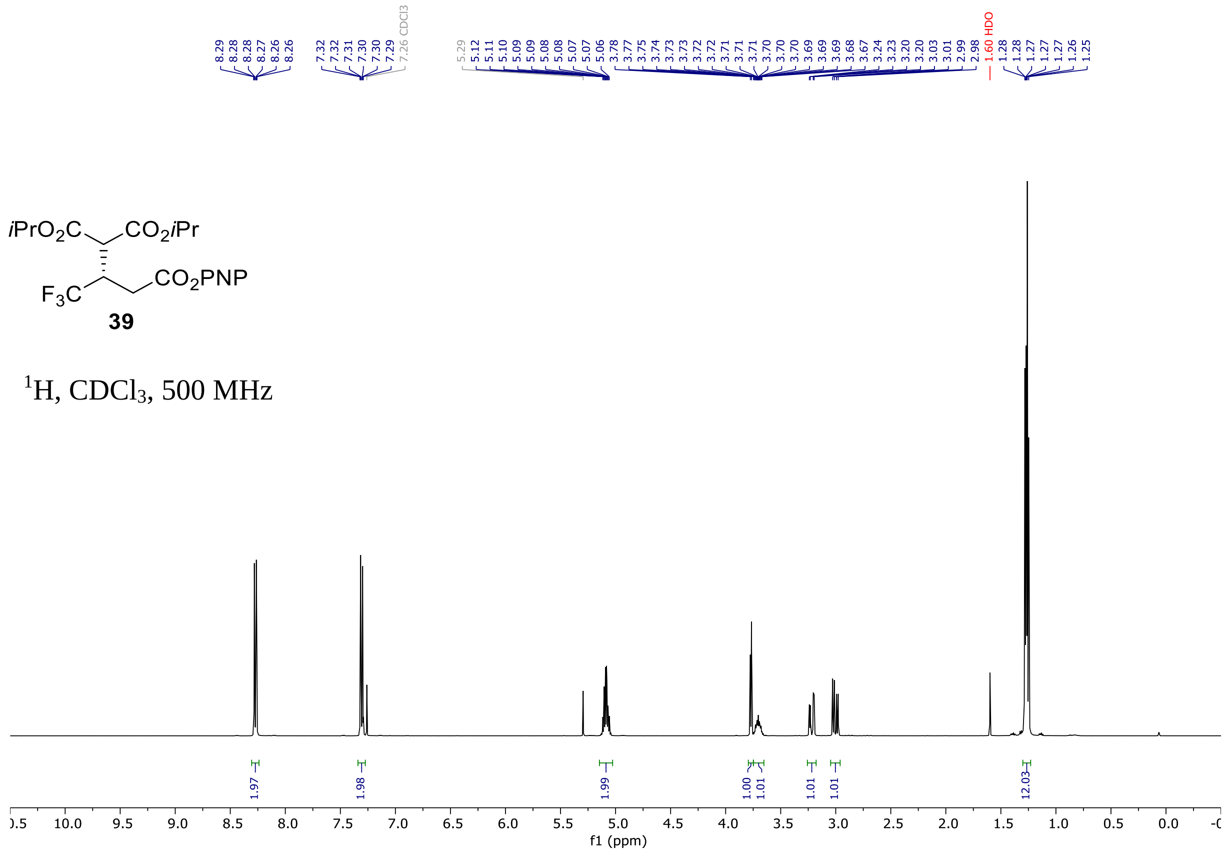
39
¹H, CDCl₃, 500 MHz
f1 (ppm)

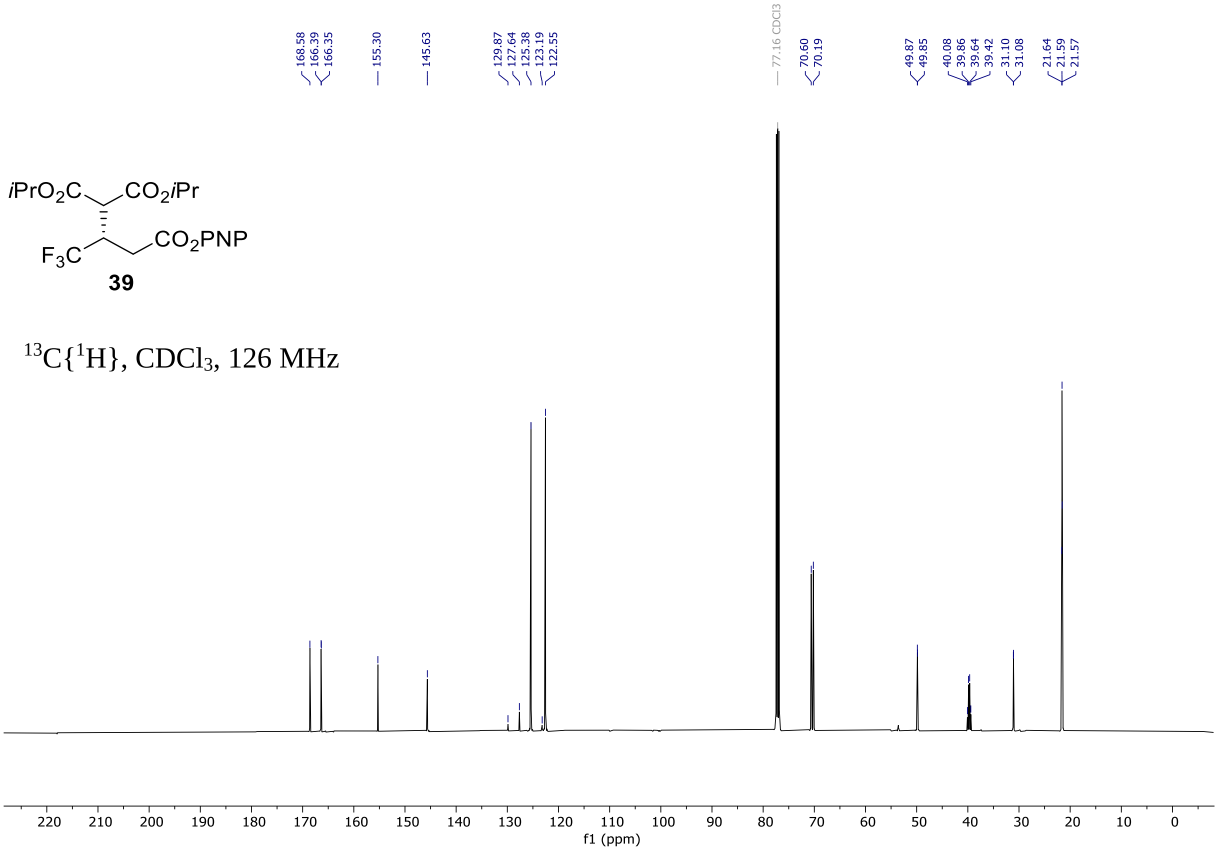
39
¹³C{¹H}, CDCl₃, 126 MHz
f1 (ppm)

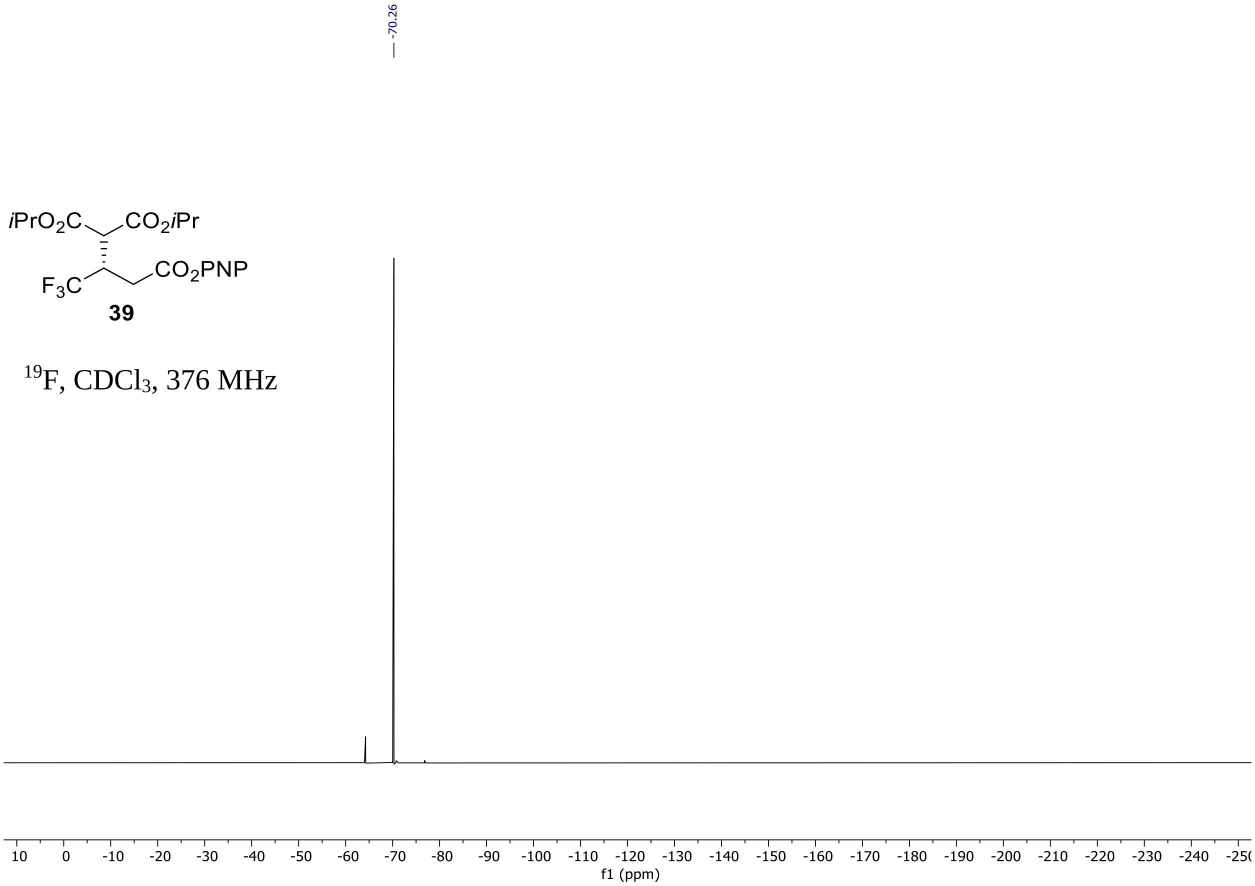
-70.26
39
19F, CDCl3, 376 MHz
10 0 -10 -20 -30 -40 -50 -60 -70 -80 -90 -100 -110 -120 -130 -140 -150 -160 -170 -180 -190 -200 -210 -220 -230 -240 -250
f1 (ppm)

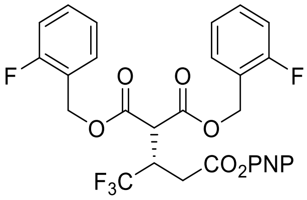

40

^1H, $CDCl_3$, 500 MHz

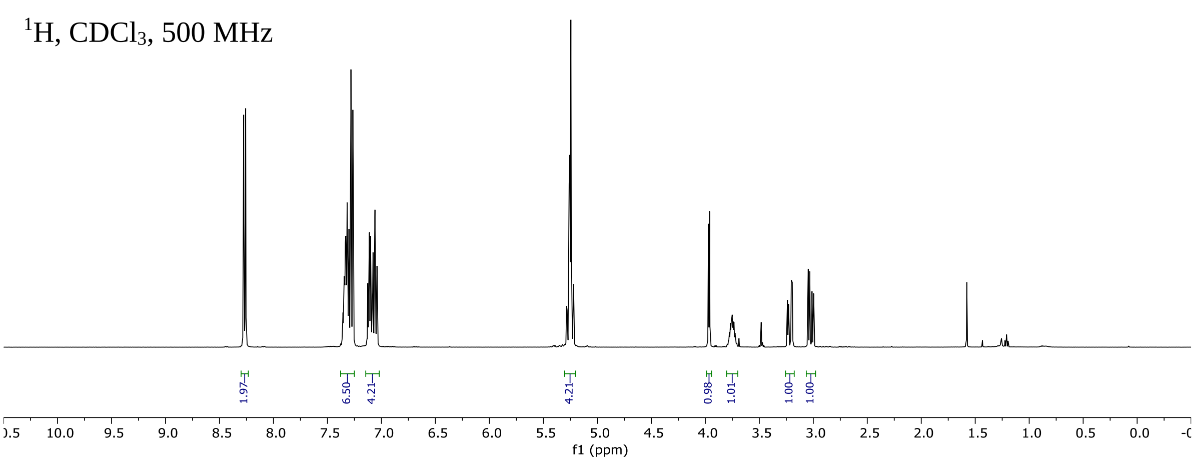

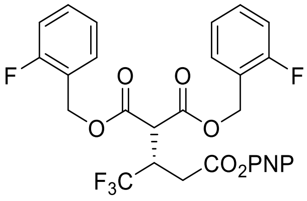

40

$^{13}C\{^1H\}$, $CDCl_3$, 126 MHz

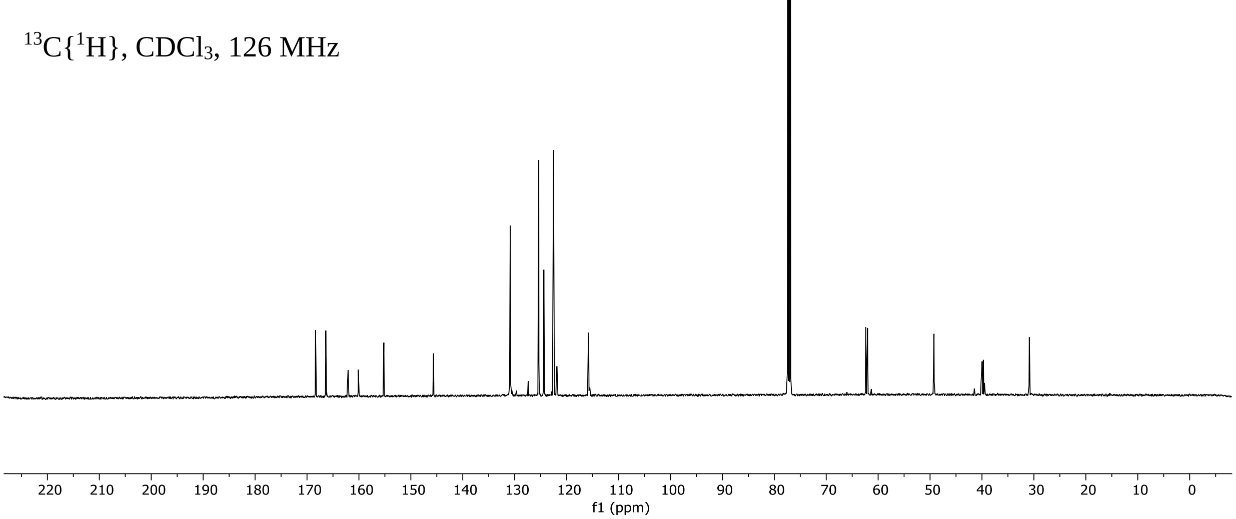

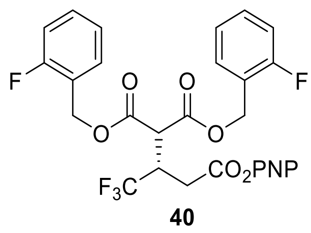

^{19}F, $CDCl_3$, 471 MHz

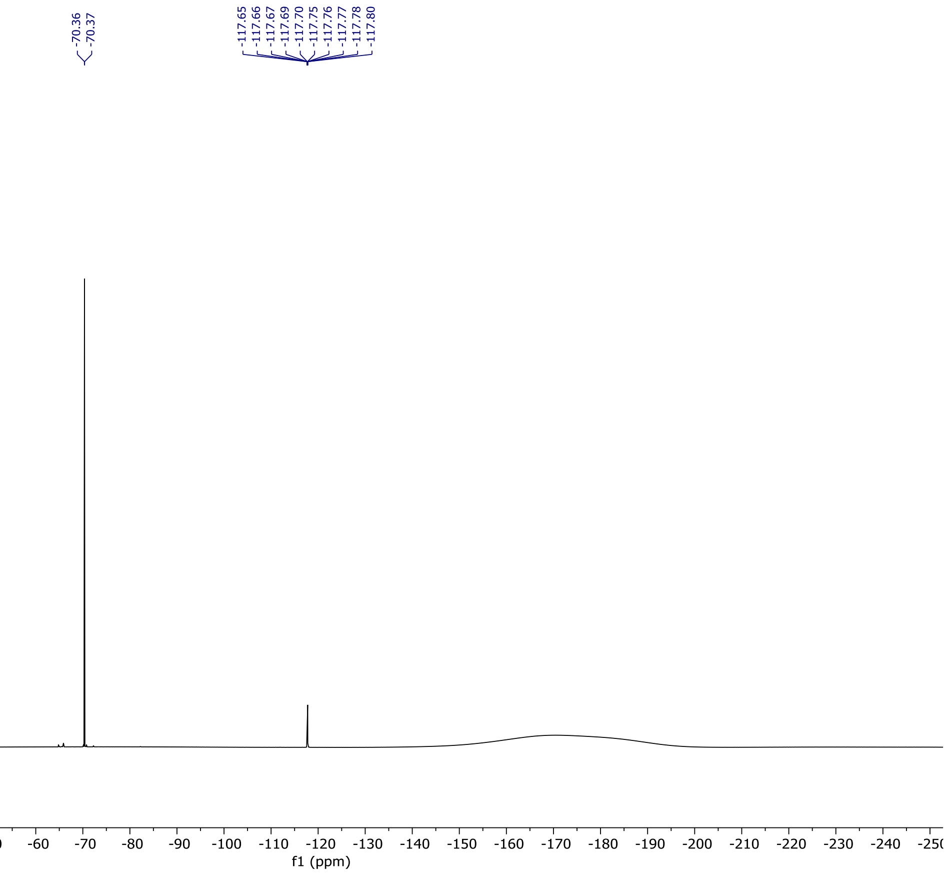

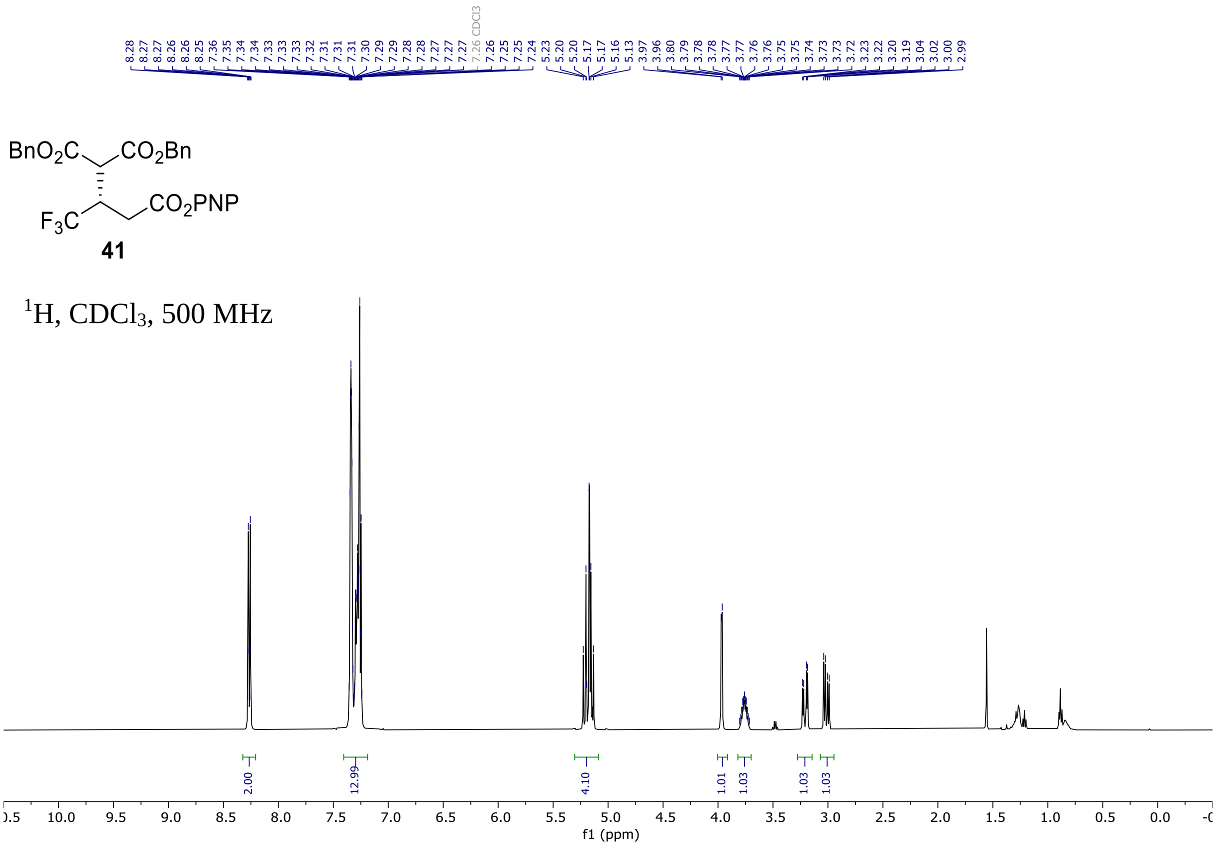
8.28 8.27 8.27 8.26 8.26 8.25 7.36 7.35 7.34 7.34 7.33 7.33 7.33 7.32 7.31 7.31 7.30 7.29 7.29 7.28 7.28 7.27 7.27 7.27 7.26 CDCl3 7.26 7.25 7.25 7.24 5.23 5.20 5.20 5.17 5.17 5.16 5.13 3.97 3.96 3.80 3.79 3.78 3.78 3.77 3.77 3.76 3.76 3.75 3.75 3.74 3.73 3.73 3.72 3.23 3.22 3.20 3.19 3.04 3.02 3.00 2.99
BnO_2C CO_2Bn F_3C CO_2PNP
41
^1H, $CDCl_3$, 500 MHz
2.00 12.99 4.10 1.01 1.03 1.03 1.03
f1 (ppm)

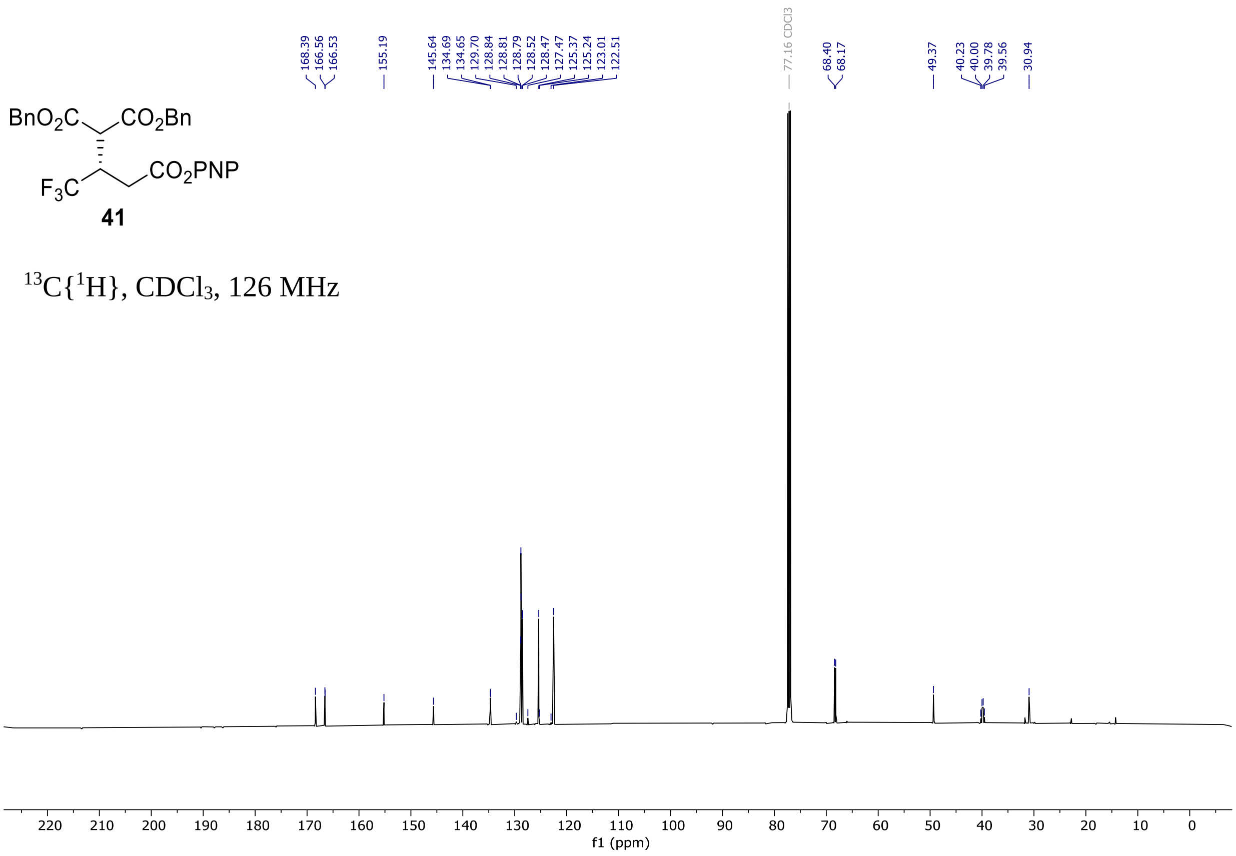
168.39 166.56 166.53 155.19 145.64 134.69 134.65 129.70 128.84 128.81 128.79 128.52 128.47 127.47 125.37 125.24 123.01 122.51 77.16 CDCl3 68.40 68.17 49.37 40.23 40.00 39.78 39.56 30.94
BnO_2C CO_2Bn F_3C CO_2PNP
41
$^{13}C\{^1H\}$, $CDCl_3$, 126 MHz
f1 (ppm)

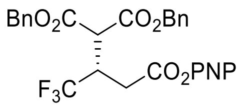

41

^{19}F, $CDCl_3$, 376 MHz

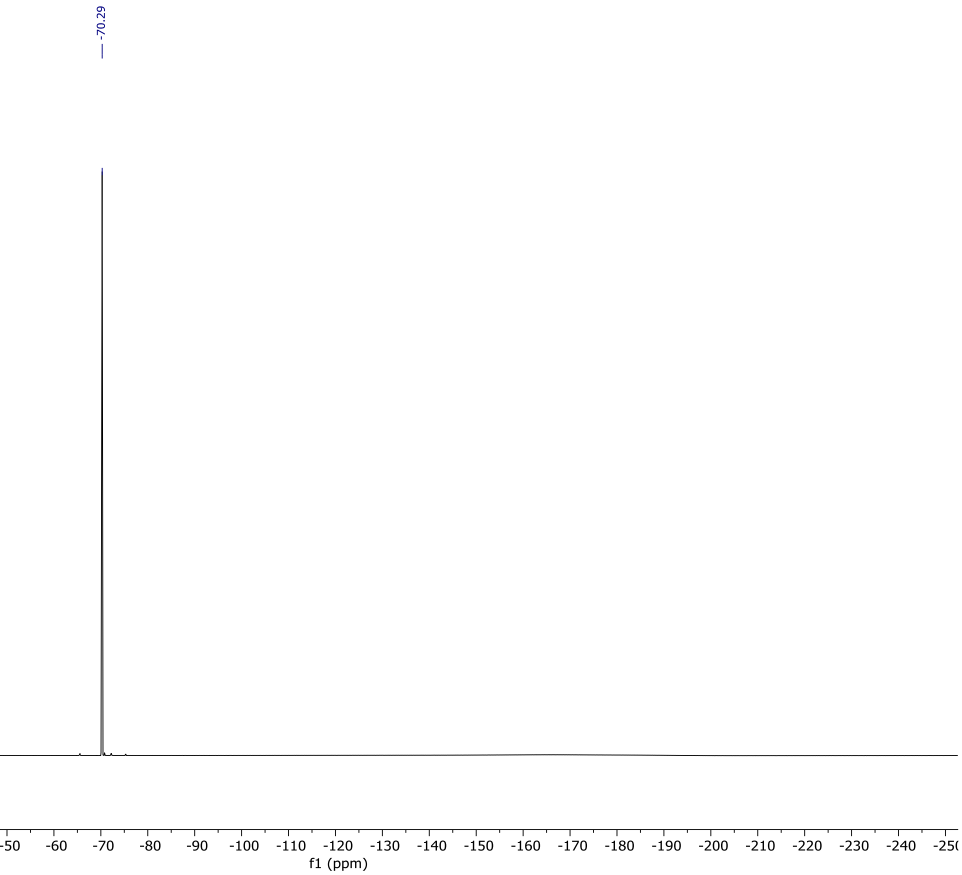

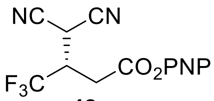

42

^{1}H, $CDCl_3$, 500 MHz

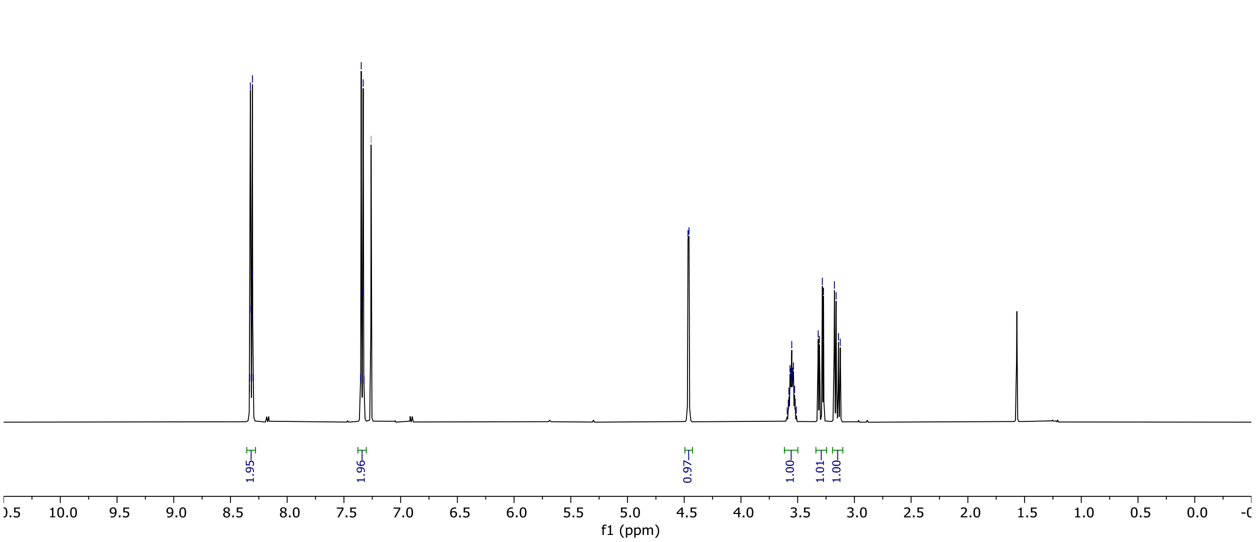

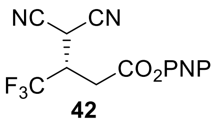

42

^{13}C{^{1}H}, $CDCl_3$, 126 MHz

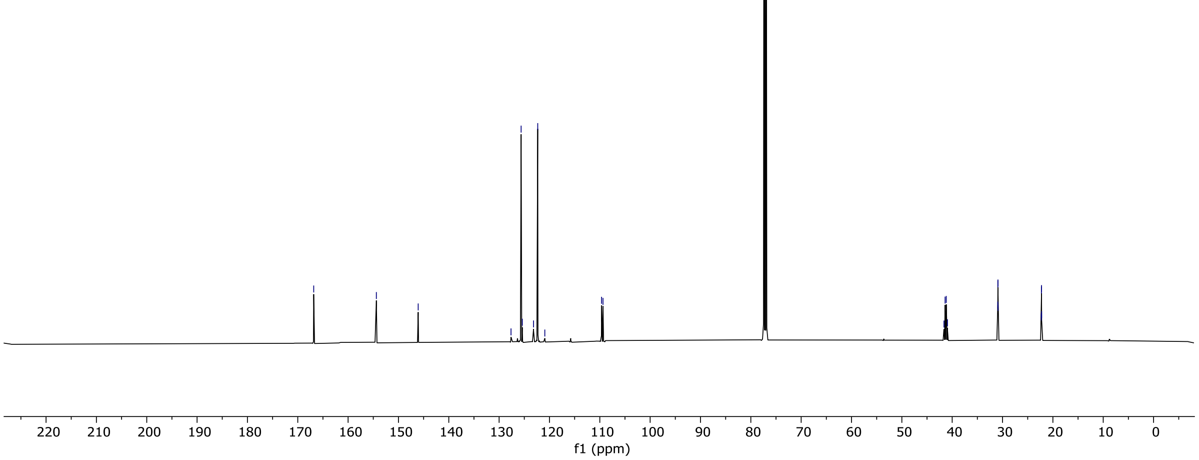

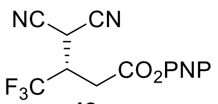

42

^{19}F, $CDCl_3$, 471 MHz

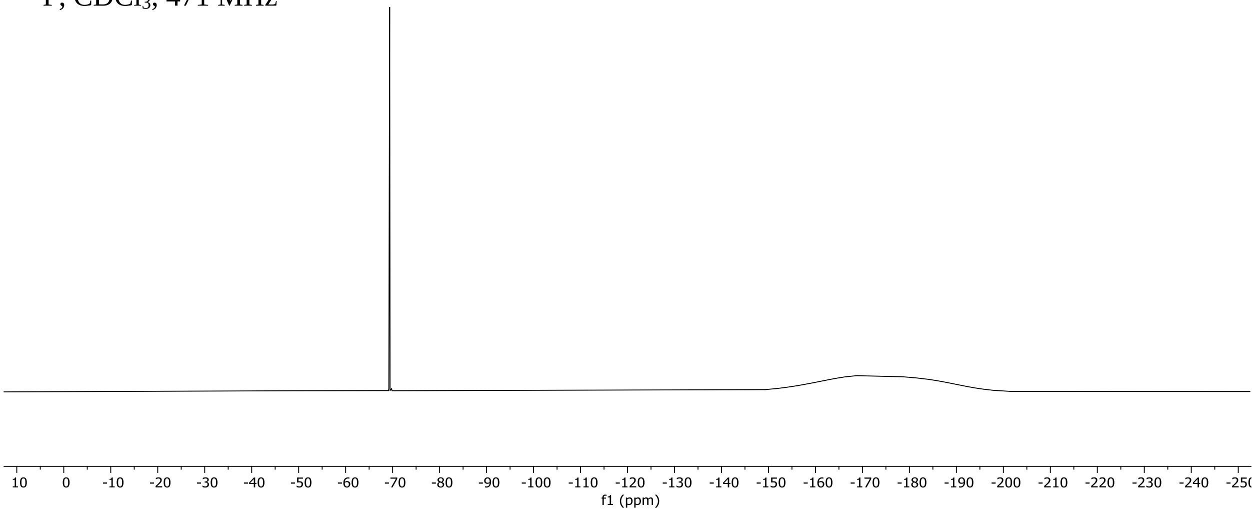

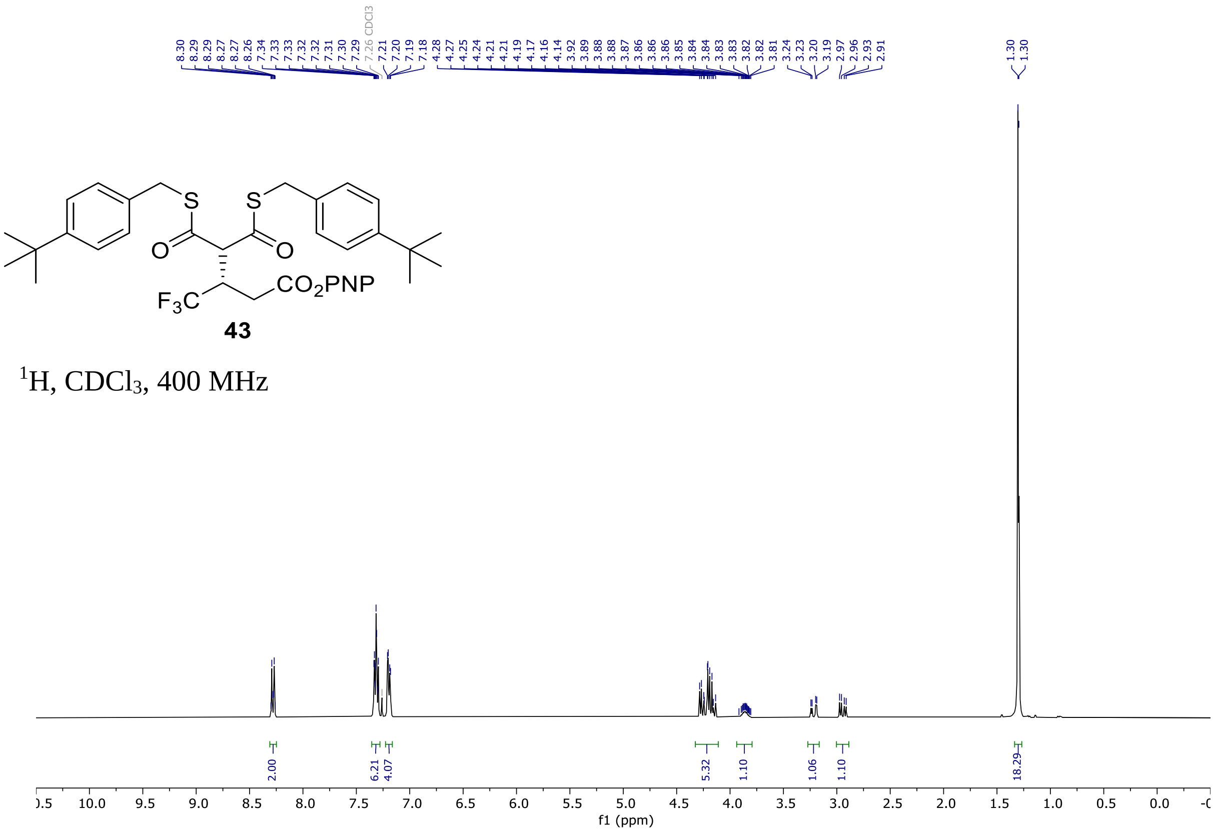

43

^1H, $CDCl_3$, 400 MHz

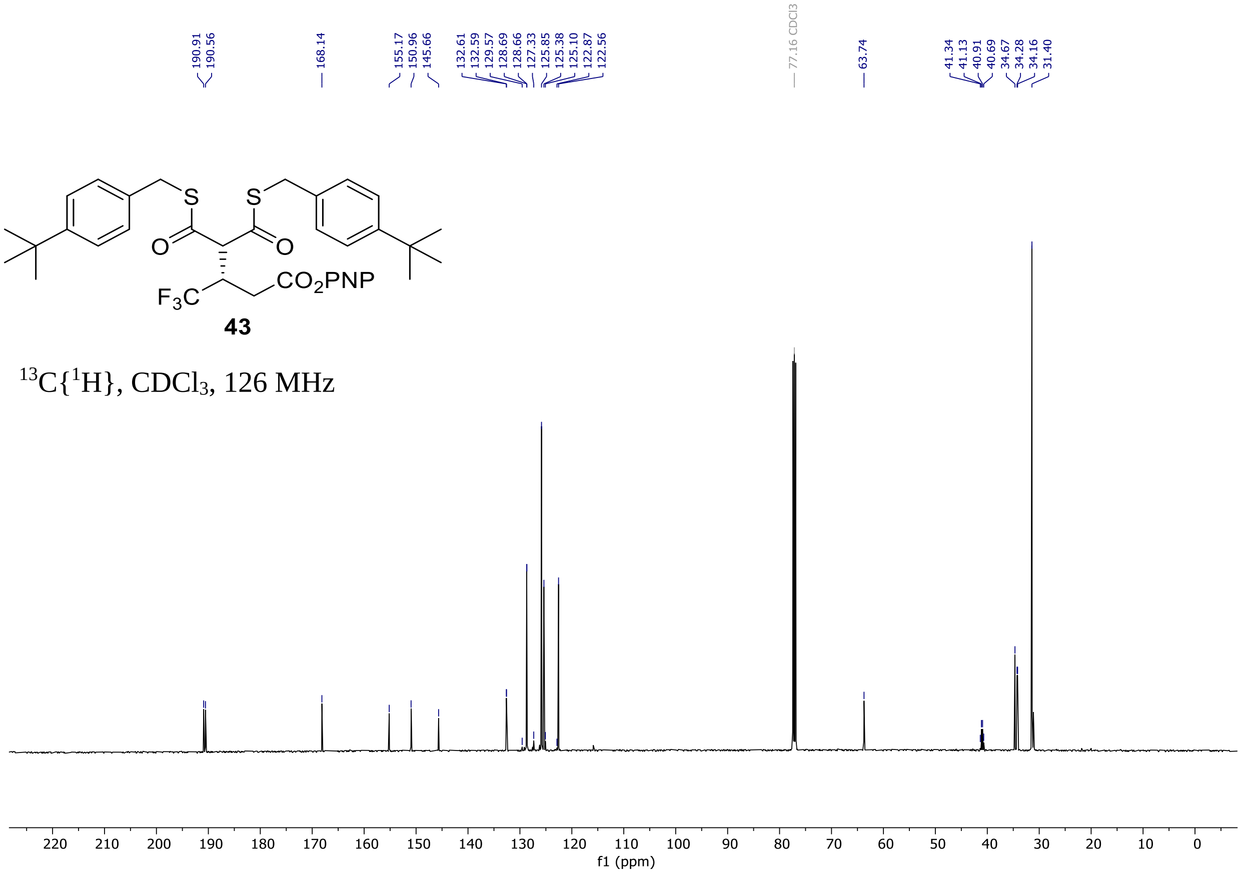

43

$^{13}C\{^1H\}$, $CDCl_3$, 126 MHz

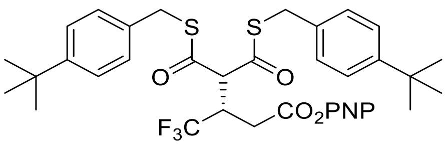

43

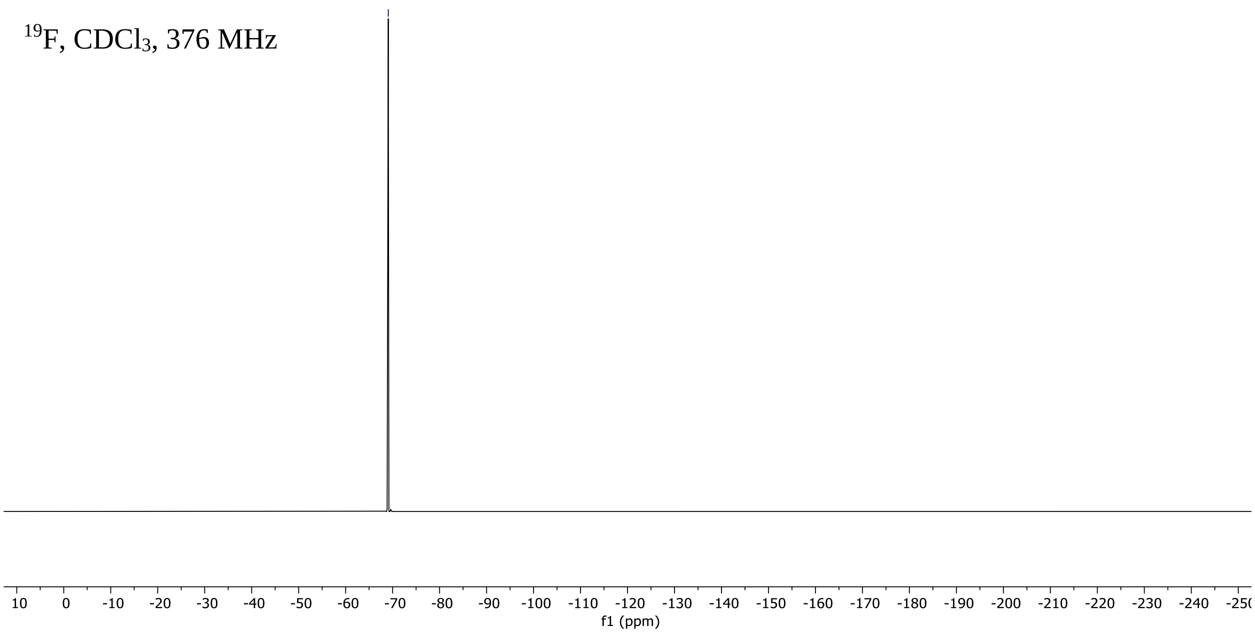

-69.08
19F, CDCl3, 376 MHz
10
0
-10
-20
-30
-40
-50
-60
-70
-80
-90
-100
-110
-120
-130
-140
-150
-160
-170
-180
-190
-200
-210
-220
-230
-240
f1 (ppm)

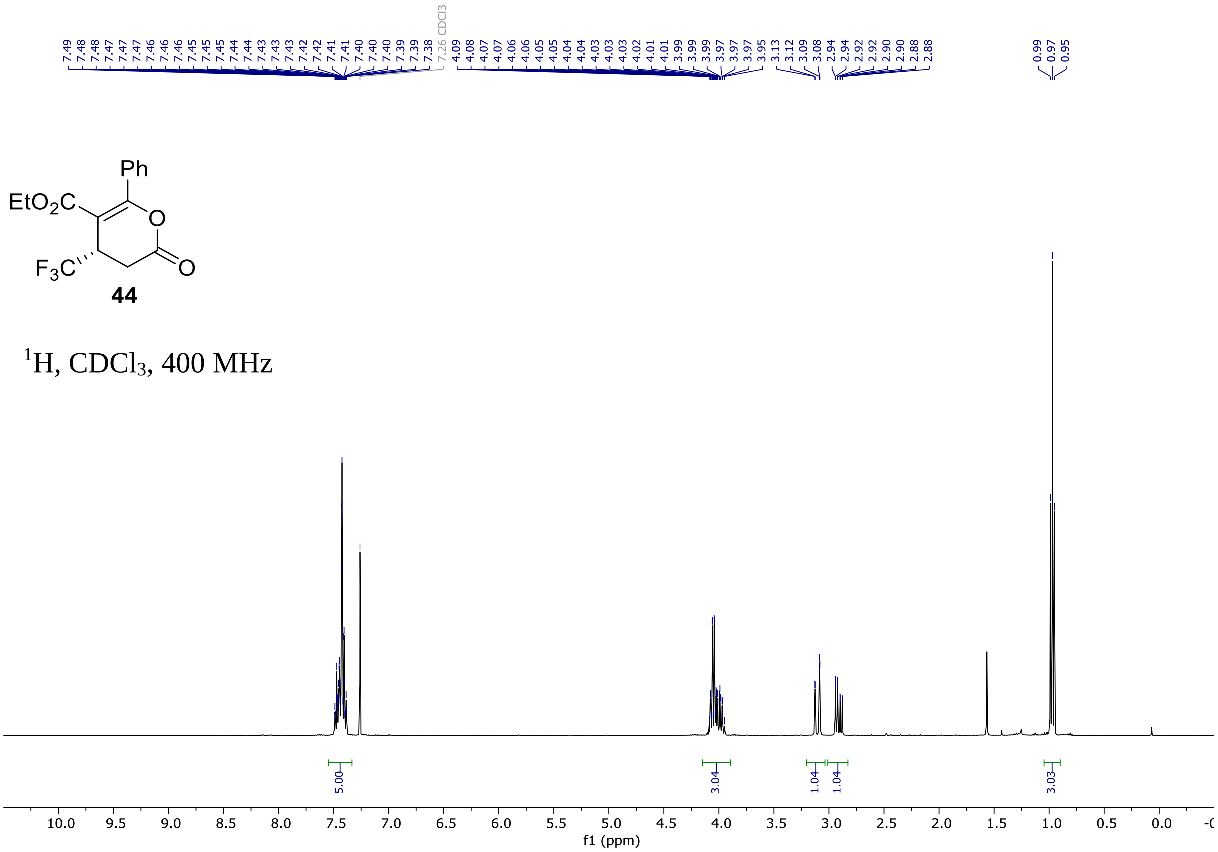
Ph
EtO2C
O
F3C
O
44
1H, CDCl3, 400 MHz
7.49 7.48 7.48 7.47 7.47 7.47 7.46 7.46 7.46 7.45 7.45 7.45 7.44 7.44 7.43 7.43 7.43 7.43 7.42 7.42 7.41 7.41 7.40 7.40 7.40 7.39 7.39 7.38
7.26 CDCl3
4.09 4.08 4.07 4.07 4.06 4.06 4.05 4.05 4.04 4.04 4.03 4.03 4.03 4.02 4.01 4.01 3.99 3.99 3.99 3.97 3.97 3.97 3.95
3.13 3.12 3.09 3.08 2.94 2.94 2.92 2.92 2.90 2.90 2.88 2.88
0.99 0.97 0.95
5.00 3.04 1.04 1.04 3.03
f1 (ppm)

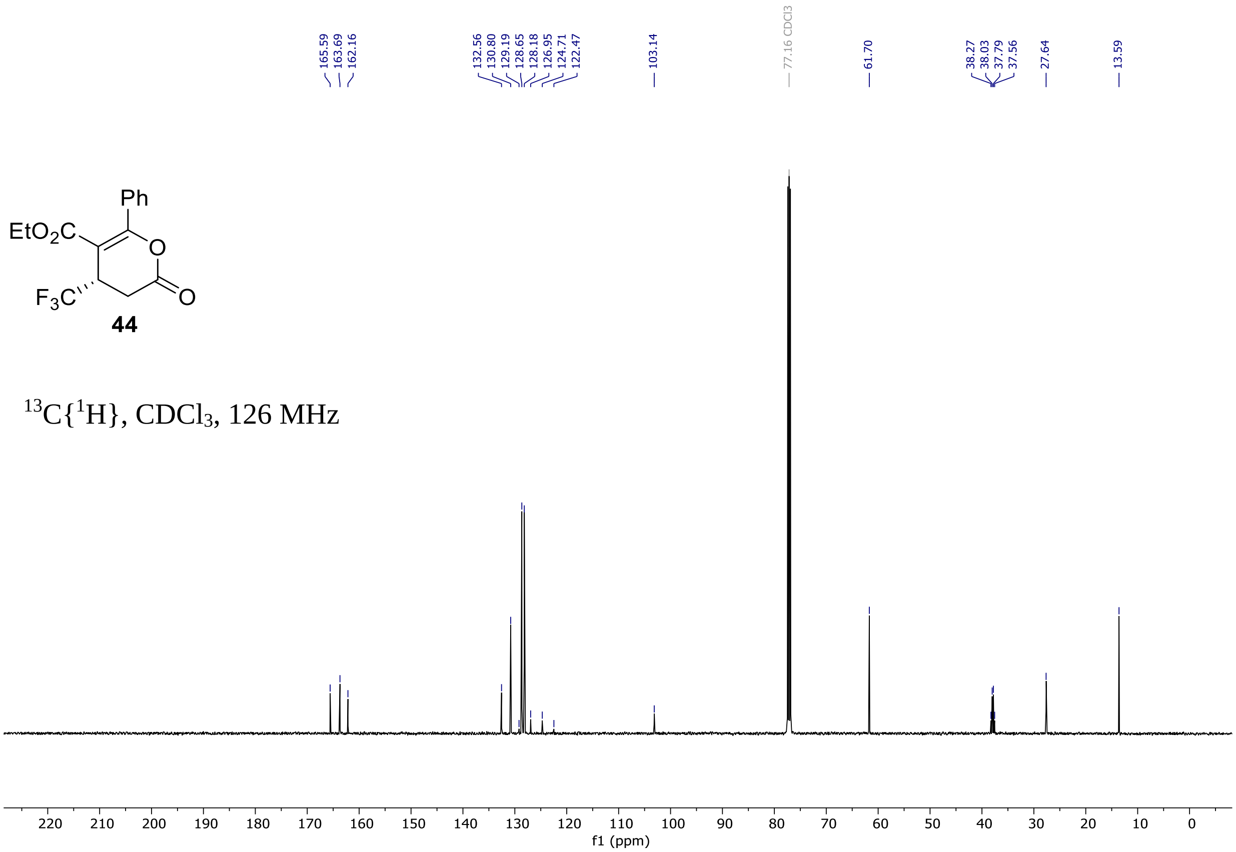
Ph
EtO2C
O
F3C
O
44
13C{1H}, CDCl3, 126 MHz
165.59 163.69 162.16
132.56 130.80 129.19 128.65 128.18 126.95 124.71 122.47
103.14
77.16 CDCl3
61.70
38.27 38.03 37.79 37.56
27.64
13.59
f1 (ppm)

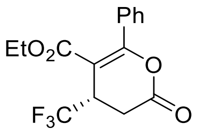

44

^{19}F, $CDCl_3$, 376 MHz

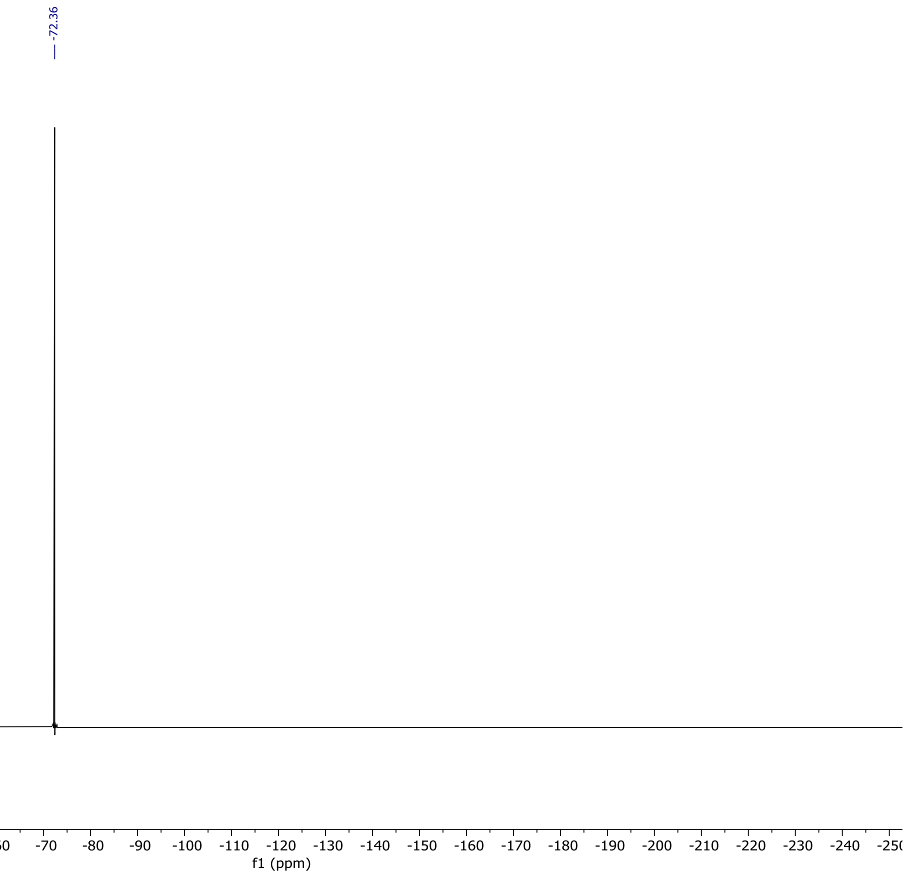

9. Appendix 2: HPLC Spectra

HPLC Data for **8a**: Chiralpak AD-H (90:10 hexane:IPA, flow rate 1.00 mL.min^{-1}, 211 nm, 30 °C) t_R (minor): 31.6 min, t_R (major): 19.3 min, >99:1 er.

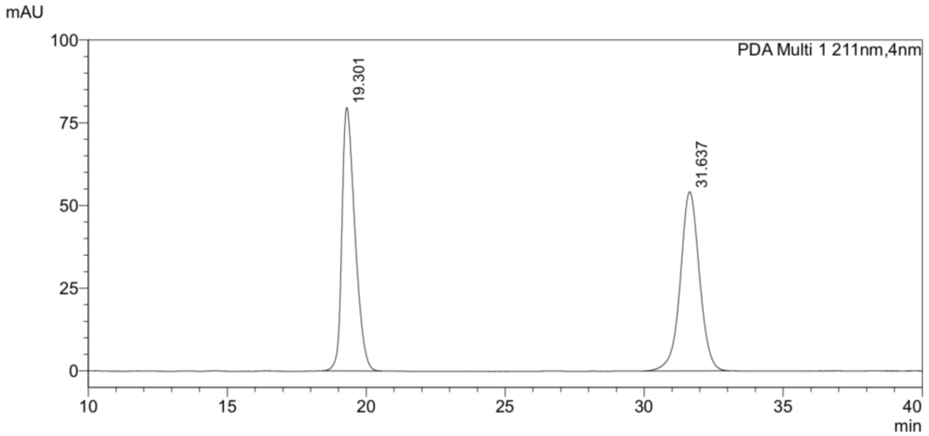

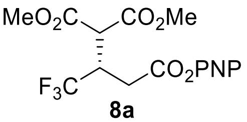

8a

PDA Ch1 211nm

Peak#	Ret. Time	Area%
1	19.301	50.003
2	31.637	49.997
Total		100.000

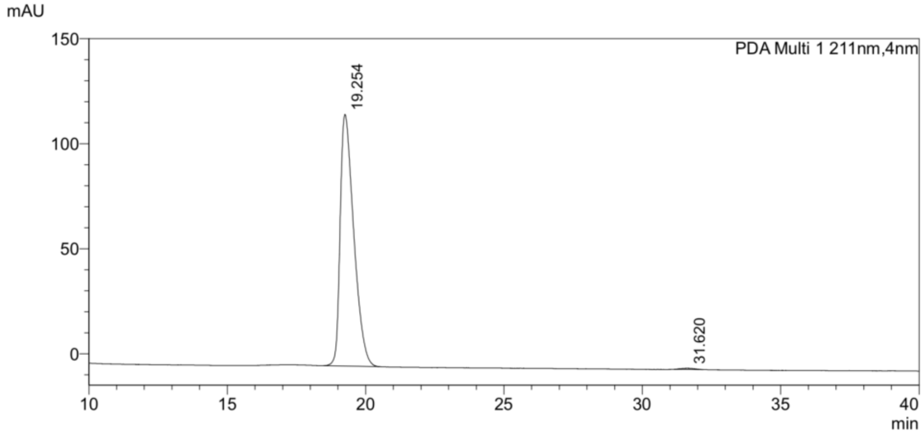

PDA Ch1 211nm

Peak#	Ret. Time	Area%
1	19.254	99.563
2	31.620	0.437
Total		100.000

HPLC Data for **8b**: Chiralpak AD-H (90:10 hexane:IPA, flow rate 1.00 mL.min^{-1}, 211 nm, 30 °C) t_R (minor): 16.5 min, t_R (major): 11.3 min, 99:1 er.

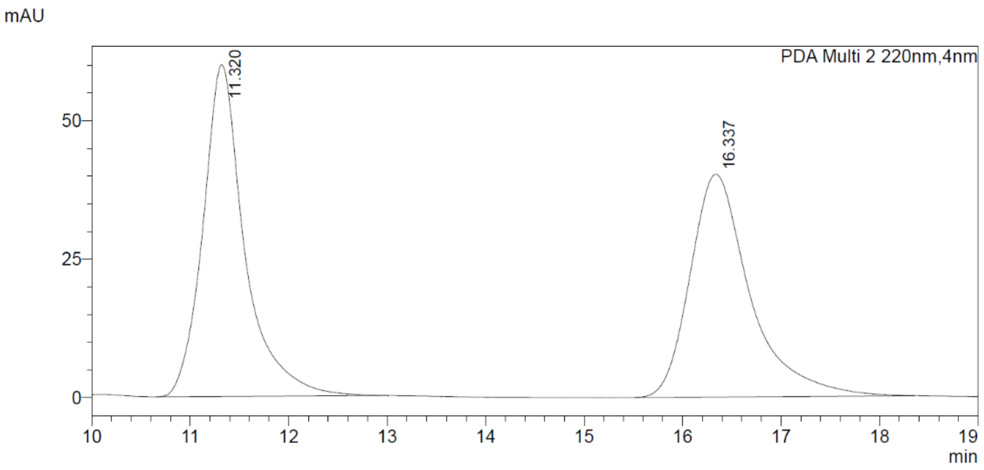

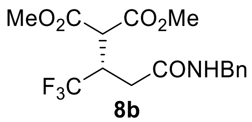

8b

PDA Ch2 220nm

Peak#	Ret. Time	Area%
1	11.320	50.890
2	16.337	49.110
Total		100.000

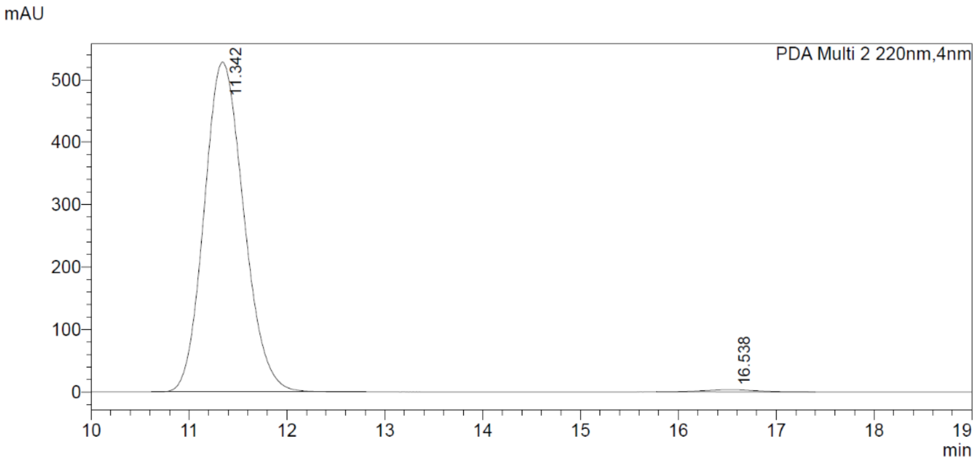

PDA Ch2 220nm

Peak#	Ret. Time	Area%
1	11.342	99.191
2	16.538	0.809
Total		100.000

HPLC Data for **18**: Chiralpak AD-H (90:10 hexane:IPA, flow rate 1.00 mL.min^{-1}, 211 nm, 30 °C) t_R (minor): 7.8 min, t_R (major): 6.5 min, >99:1 er.

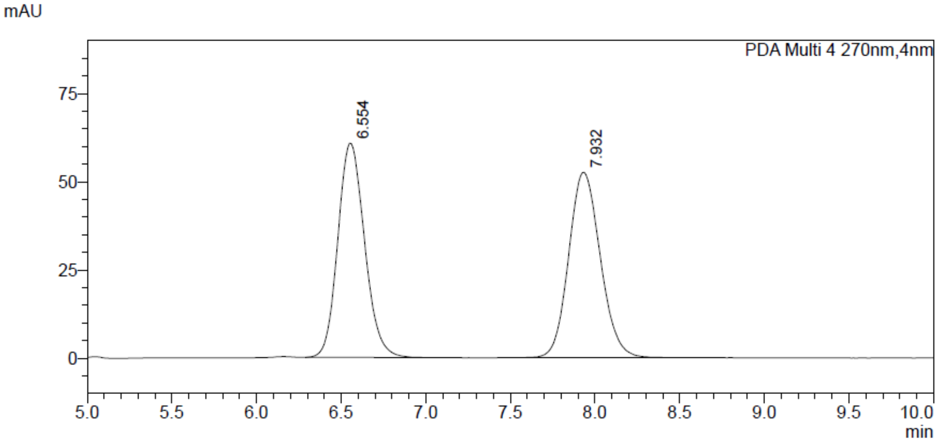

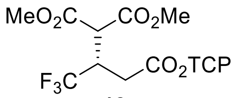

18

PDA Ch4 270nm

Peak#	Ret. Time	Area%
1	6.554	49.469
2	7.932	50.531
Total		100.000

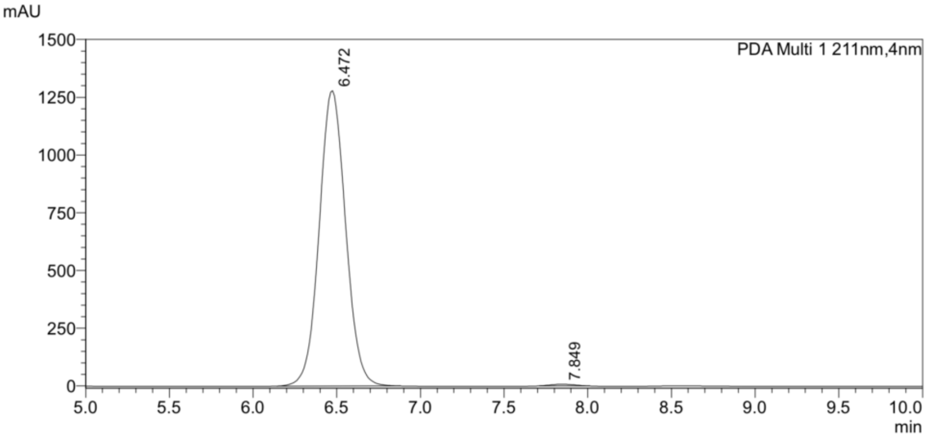

PDA Ch1 211nm

Peak#	Ret. Time	Area%
1	6.472	99.491
2	7.849	0.509
Total		100.000

HPLC Data for **19**: Chiralcel OD-H (85:15 hexane:IPA, flow rate 1.00 mL.min^{-1}, 211 nm, 30 °C) t_R (minor): 18.7 min, t_R (major): 17.2 min, 85:15 er.

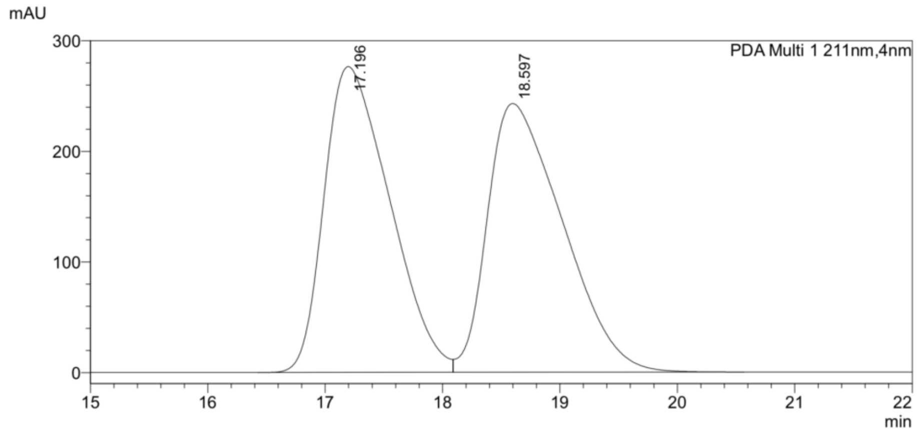

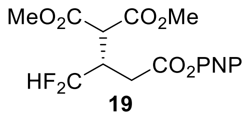

19

PDA Ch1 211nm

Peak#	Ret. Time	Area%
1	17.196	49.843
2	18.597	50.157
Total		100.000

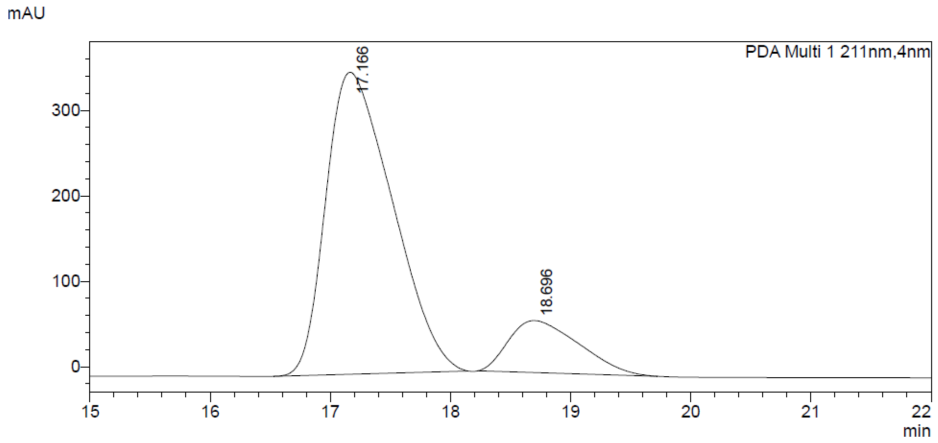

PDA Ch1 211nm

Peak#	Ret. Time	Area%
1	17.166	84.597
2	18.696	15.403
Total		100.000

HPLC Data for **20**: Chiralpak AD-H (90:10 hexane:IPA, flow rate 1.00 mL.min^{-1}, 211 nm, 30 °C) t_R (minor): 24.2 min, t_R (major): 11.9 min, >99:1 er.

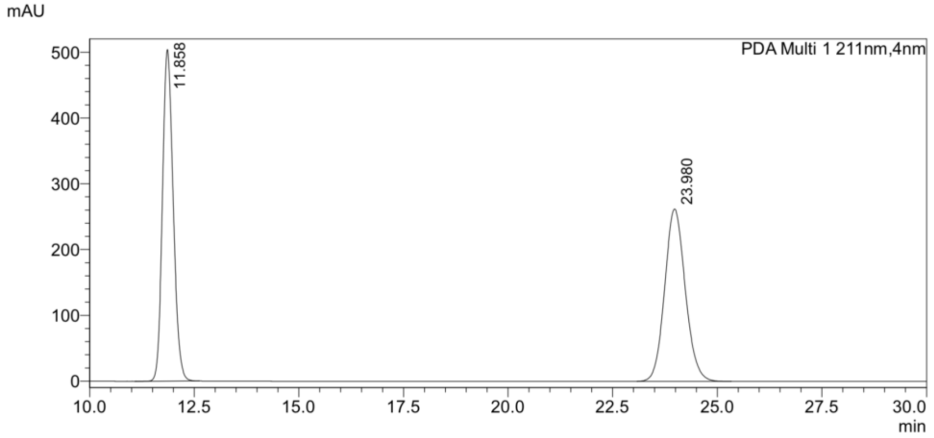

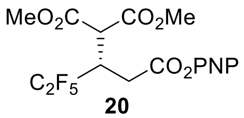

20

PDA Ch1 211nm

Peak#	Ret. Time	Area%
1	11.858	50.069
2	23.980	49.931
Total		100.000

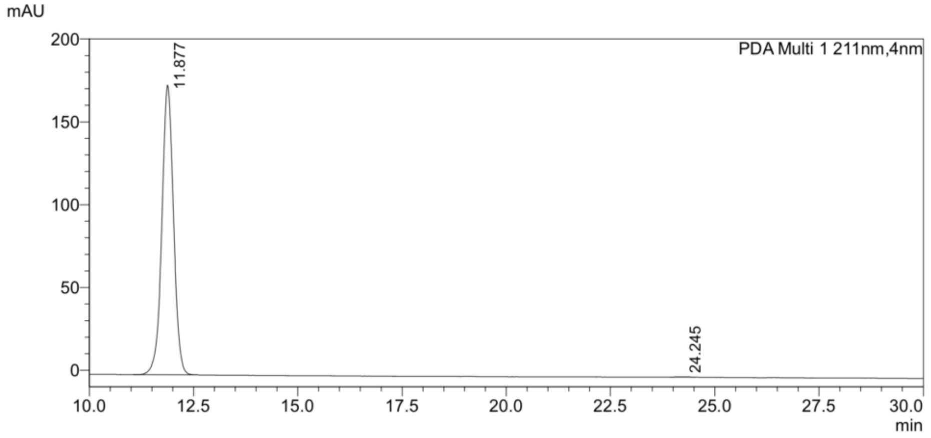

PDA Ch1 211nm

Peak#	Ret. Time	Area%
1	11.877	99.902
2	24.245	0.098
Total		100.000

HPLC Data for **21**: Chiralpak AD-H (90:10 hexane:IPA, flow rate 1.00 mL.min^{-1}, 211 nm, 30 °C) t_R (minor): 21.0 min, t_R (major): 29.8 min, >99:1 er.

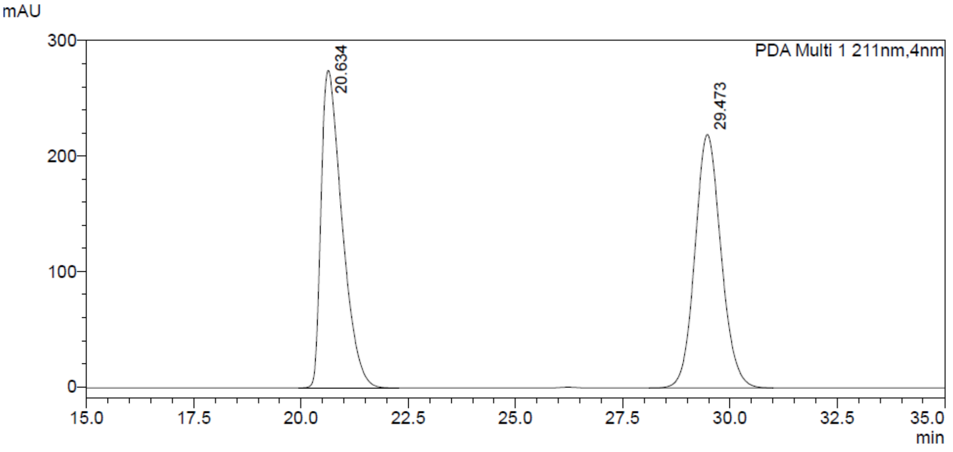

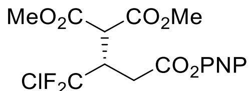

21

PDA Ch1 211nm

Peak#	Ret. Time	Area%
1	20.634	50.105
2	29.473	49.895
Total		100.000

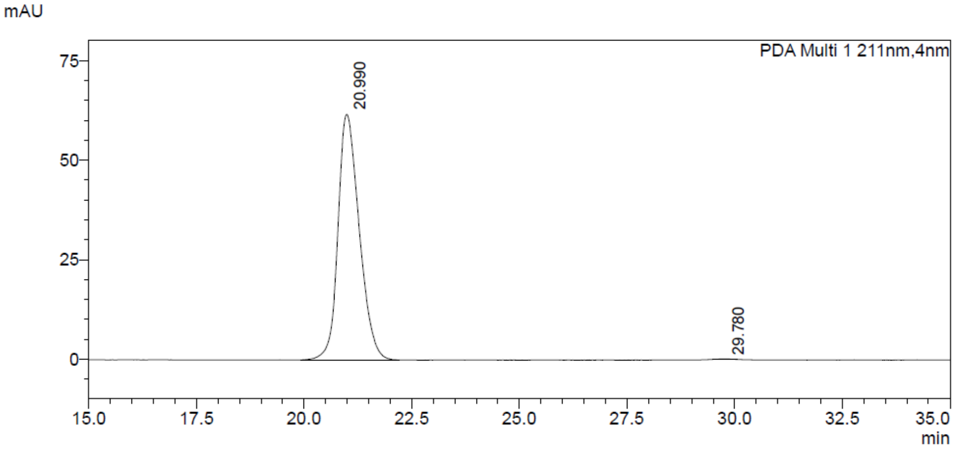

PDA Ch1 211nm

Peak#	Ret. Time	Area%
1	20.990	99.881
2	29.780	0.119
Total		100.000

HPLC Data for **22**: Chiralpak AD-H (90:10 hexane:IPA, flow rate 1.00 mL.min^{-1}, 211 nm, 30 °C) t_R (minor): 30.9 min, t_R (major): 23.8 min, >99:1 er.

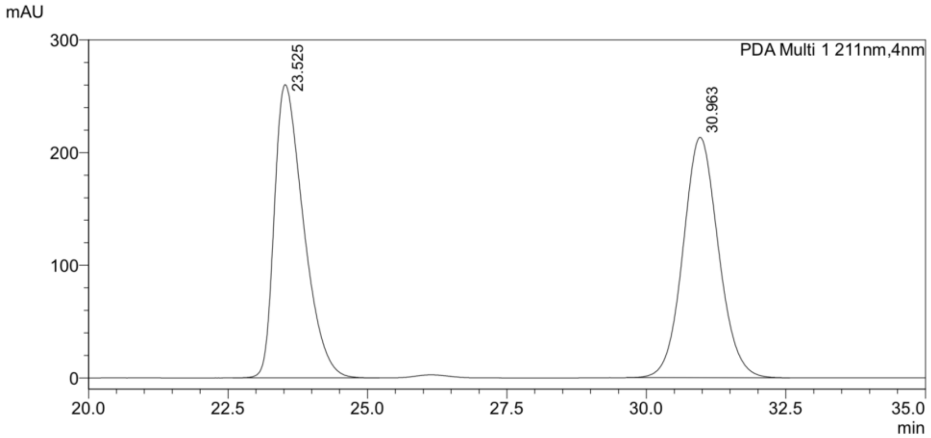

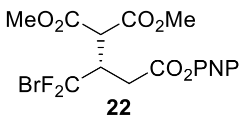

22

PDA Ch1 211nm

Peak#	Ret. Time	Area%
1	23.525	50.131
2	30.963	49.869
Total		100.000

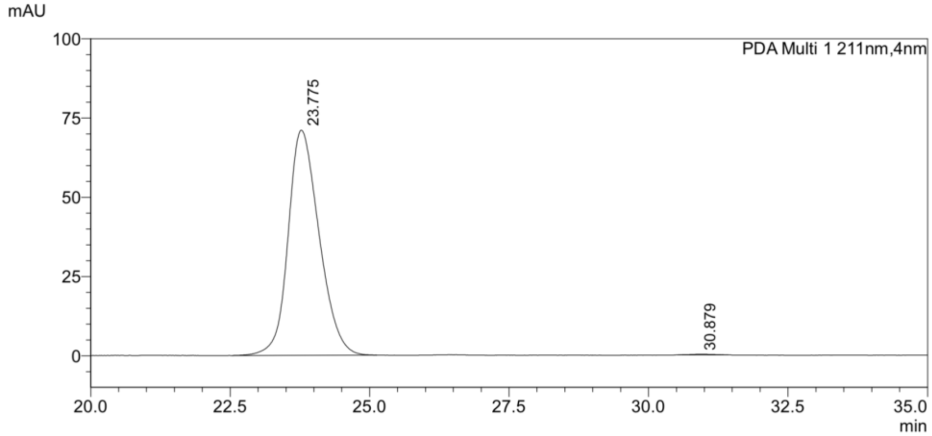

PDA Ch1 211nm

Peak#	Ret. Time	Area%
1	23.775	99.770
2	30.879	0.230
Total		100.000

HPLC Data for **23**: Chiralpak AD-H (90:10 hexane:IPA, flow rate 1.00 mL.min^{-1}, 211 nm, 30 °C) t_R (minor): 53.8 min, t_R (major): 41.5 min, 98:2 er.

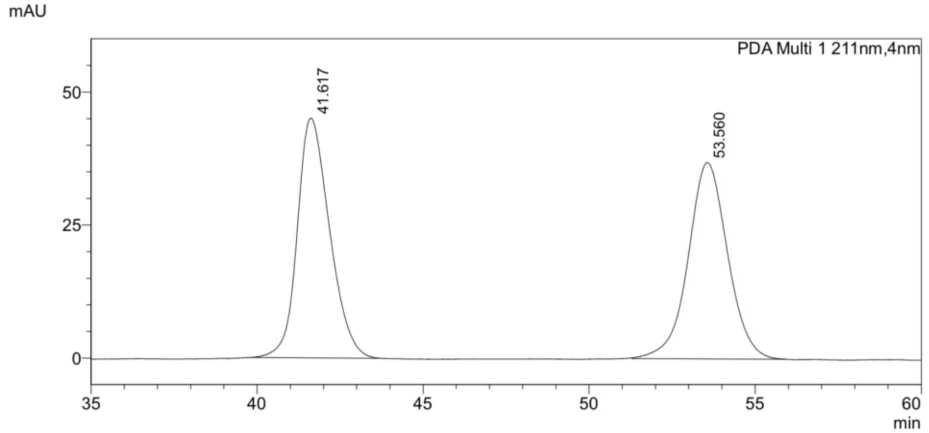

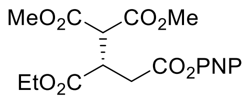

23

PDA Ch1 211nm

Peak#	Ret. Time	Area%
1	41.617	49.991
2	53.560	50.009
Total		100.000

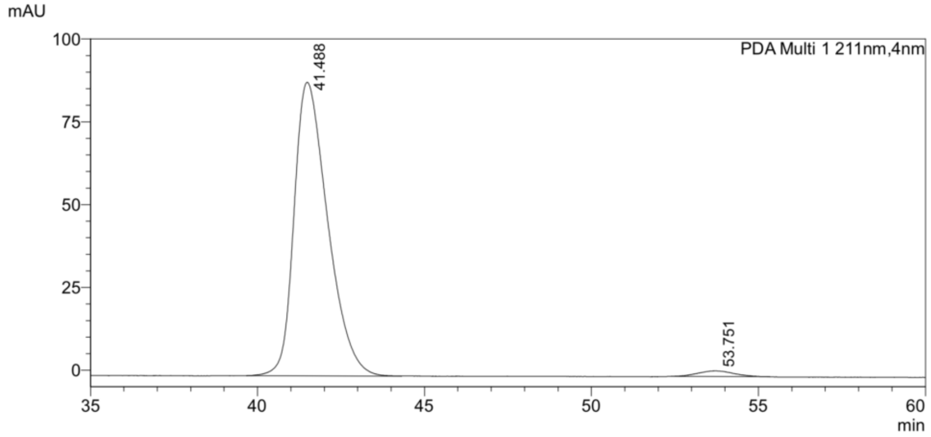

PDA Ch1 211nm

Peak#	Ret. Time	Area%
1	41.488	97.988
2	53.751	2.012
Total		100.000

HPLC Data for **24**: Chiralpak AS-H (95:5 hexane:IPA, flow rate 1.00 mL.min^{-1}, 211 nm, 40 °C) t_R (major): 27.5 min, t_R (minor): 24.7 min, 97:3 er.

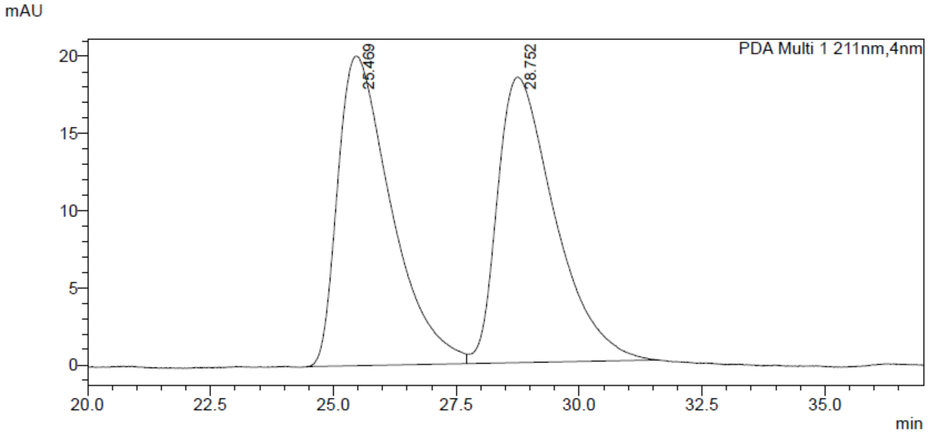

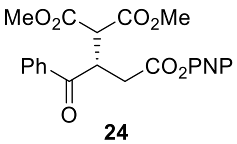

24

PDA Ch1 211nm

Peak#	Ret. Time	Area%
1	25.469	50.232
2	28.752	49.768
Total		100.000

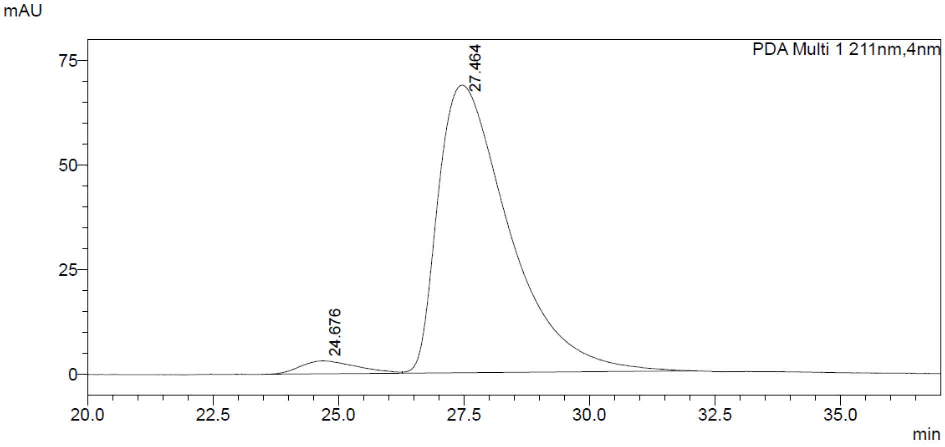

PDA Ch1 211nm

Peak#	Ret. Time	Area%
1	24.676	3.399
2	27.464	96.601
Total		100.000

HPLC Data for **37**: Chiralcel OD-H (90:10 hexane:IPA, flow rate 1.00 mL.min^{-1}, 211 nm, 30 °C) t_R (minor): 22.8 min, t_R (major): 15.6 min, 98:2 er.

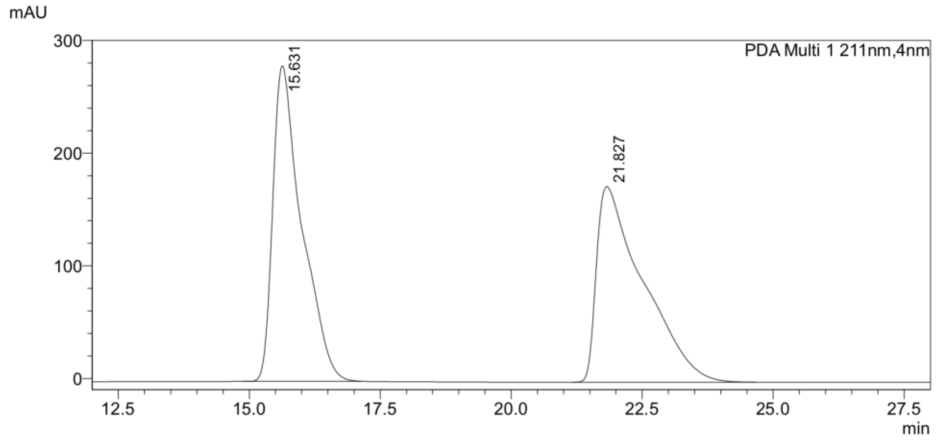

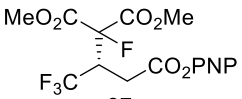

37

PDA Ch1 211nm

Peak#	Ret. Time	Area%
1	15.631	49.995
2	21.827	50.005
Total		100.000

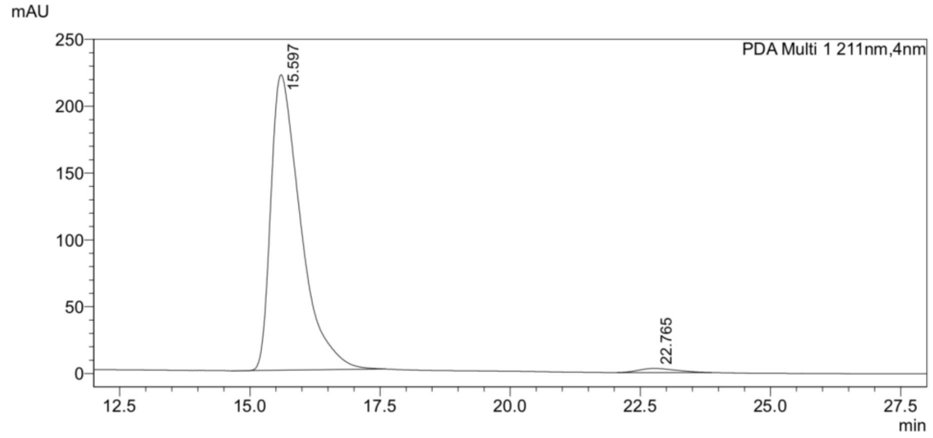

PDA Ch1 211nm

Peak#	Ret. Time	Area%
1	15.597	98.295
2	22.765	1.705
Total		100.000

HPLC Data for **38**: Chiralpak AD-H (90:10 hexane:IPA, flow rate 1.00 mL.min^{-1}, 211 nm, 30 °C) t_R (minor): 24.8 min, t_R (major): 15.4 min, >99:1 er.

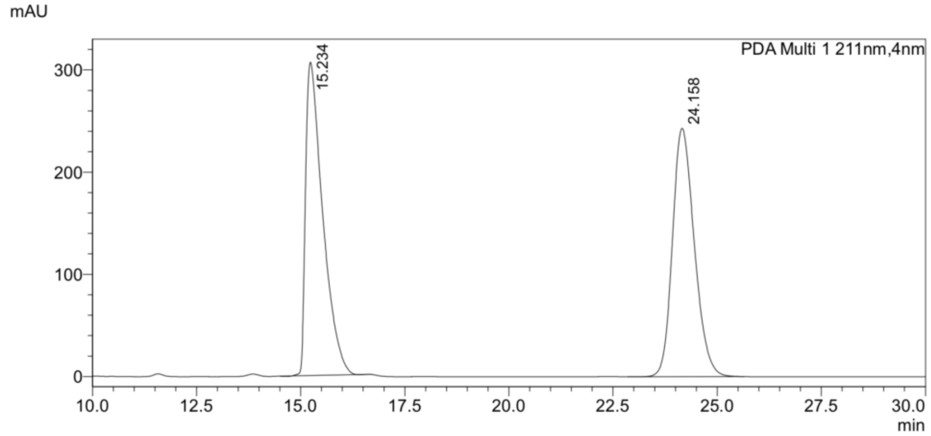

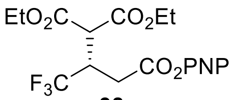

38

PDA Ch1 211nm

Peak#	Ret. Time	Area%
1	15.234	49.754
2	24.158	50.246
Total		100.000

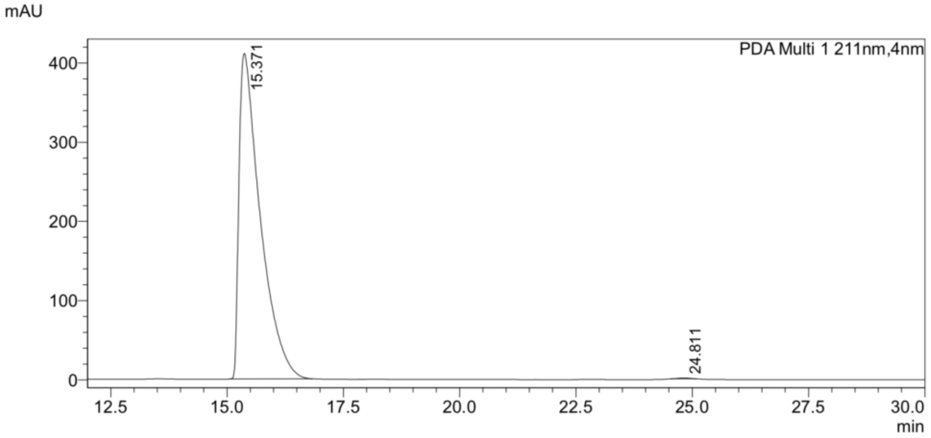

PDA Ch1 211nm

Peak#	Ret. Time	Area%
1	15.371	99.852
2	24.811	0.148
Total		100.000

HPLC Data for **39**: Chiralcel OD-H (99:1 hexane:IPA, flow rate 1.00 mL.min^{-1}, 211 nm, 30 °C) t_R (minor): 18.6 min, t_R (major): 15.1 min, >99:1 er.

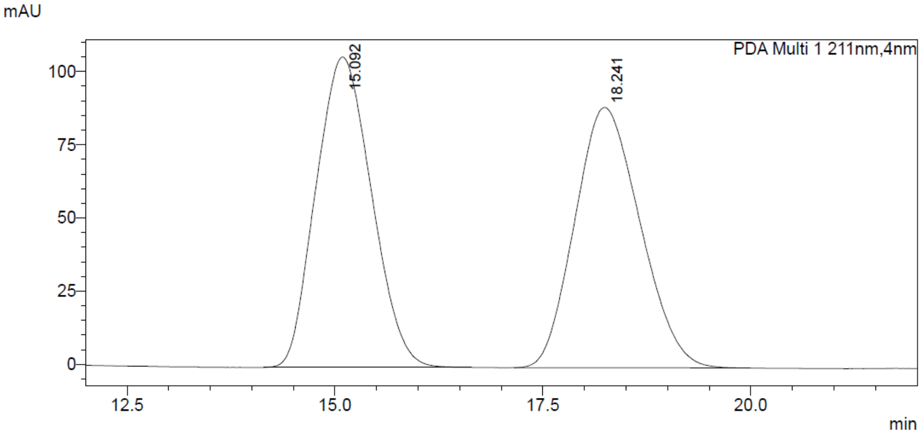

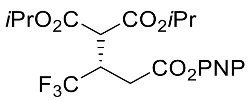

39

PDA Ch1 211nm

Peak#	Ret. Time	Area%
1	15.092	50.368
2	18.241	49.632
Total		100.000

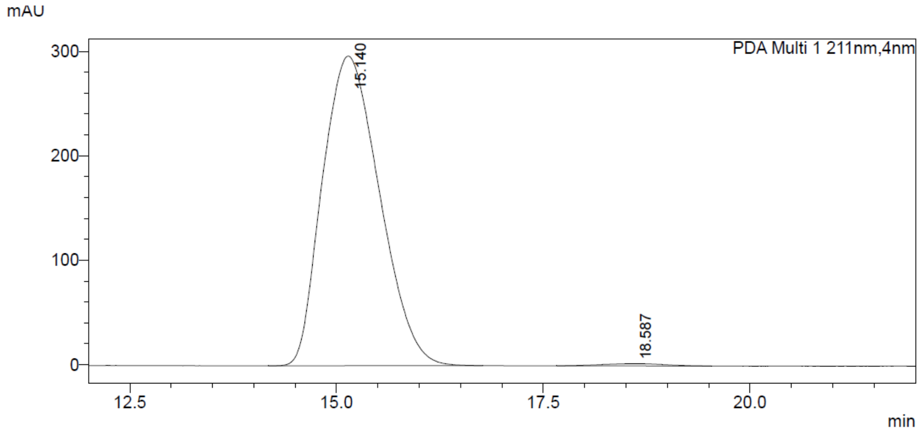

PDA Ch1 211nm

Peak#	Ret. Time	Area%
1	15.140	99.227
2	18.587	0.773
Total		100.000

HPLC Data for **40**: Chiralcel OD-H (90:10 hexane:IPA, flow rate 1.00 mL.min^{-1}, 211 nm, 30 °C) t_R (minor): 35.1 min, t_R (major): 24.0 min, 99:1 er.

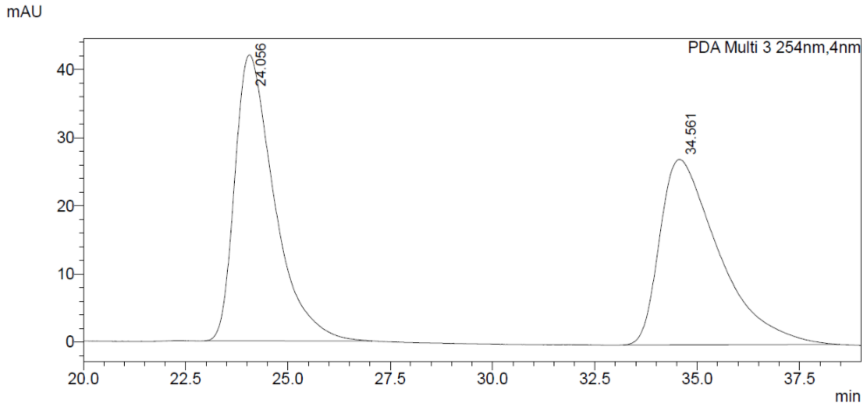

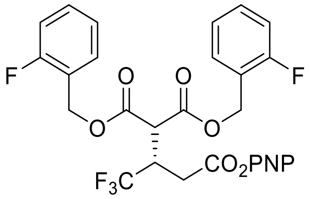

40

PDA Ch1 211nm

Peak#	Ret. Time	Area%
1	24.059	50.515
2	34.560	49.485
Total		100.000

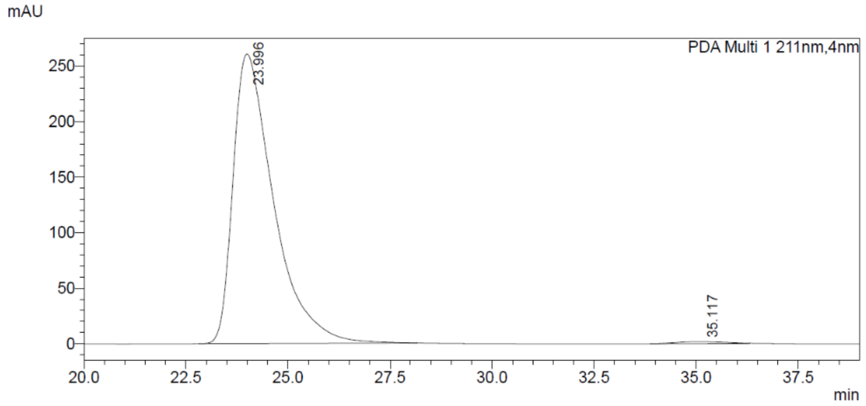

PDA Ch1 211nm

Peak#	Ret. Time	Area%
1	23.996	99.185
2	35.117	0.815
Total		100.000

HPLC Data for **41**: Chiralpak AD-H (90:10 hexane:IPA, flow rate 1.00 mL.min^{-1}, 211 nm, 30 °C) t_R (minor): 30.3 min, t_R (major): 34.3 min, >99:1 er.

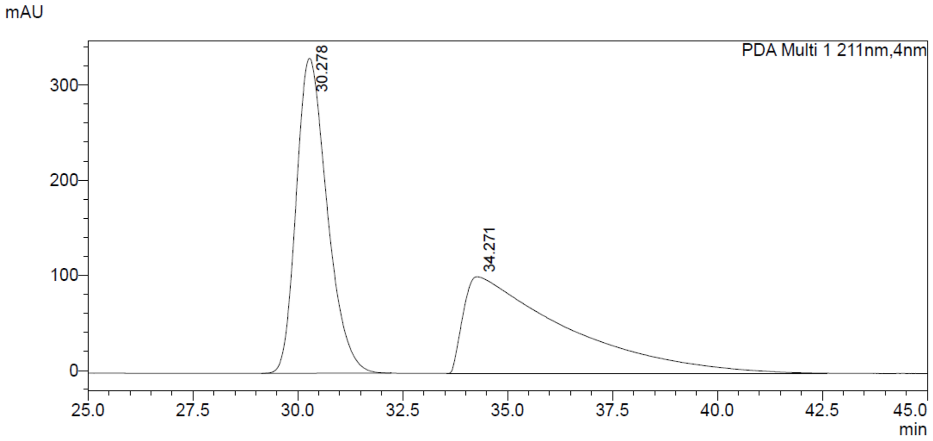

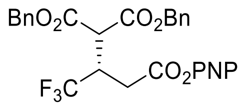

41

PDA Ch1 211nm

Peak#	Ret. Time	Area%
1	30.278	49.905
2	34.271	50.095
Total		100.000

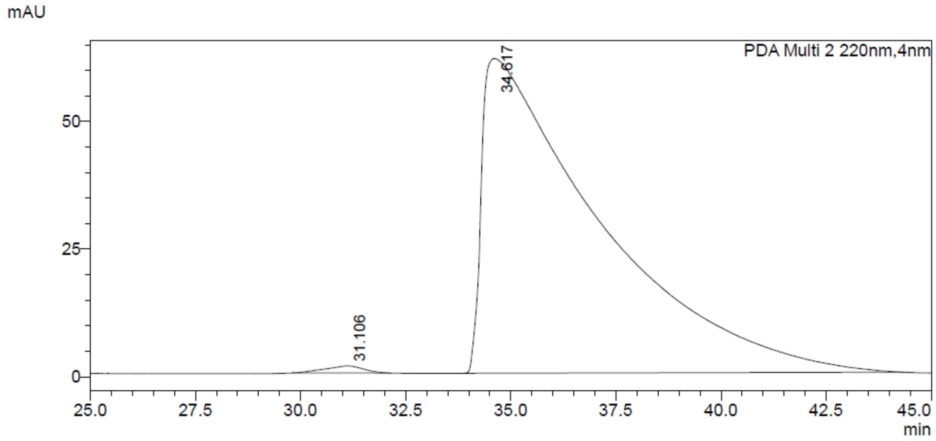

PDA Ch1 211nm

Peak#	Ret. Time	Area%
1	31.114	0.791
2	34.615	99.209
Total		100.000

HPLC Data for **42**: Chiralpak AD-H (95:5 hexane:IPA, flow rate 1.00 mL.min^{-1}, 211 nm, 30 °C) t_R (minor): 29.4 min, t_R (major): 89.5 min, >99:1 er.

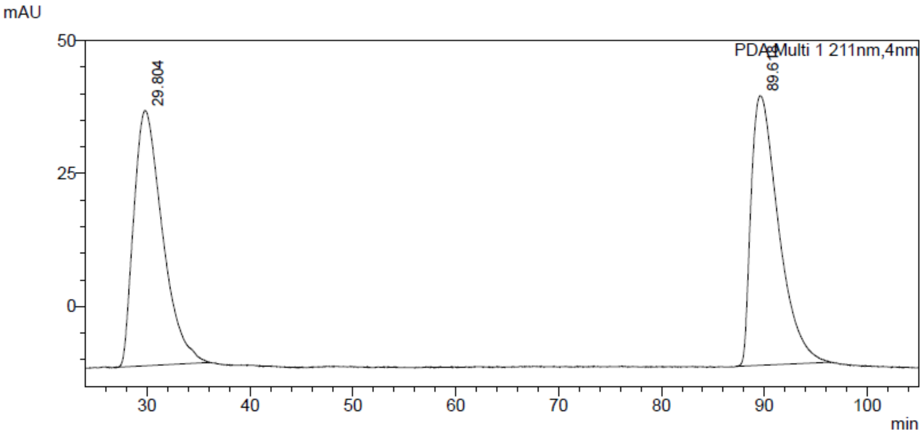

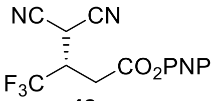

42

PDA Ch1 211nm

Peak#	Ret. Time	Area%
1	29.804	49.689
2	89.618	50.311
Total		100.000

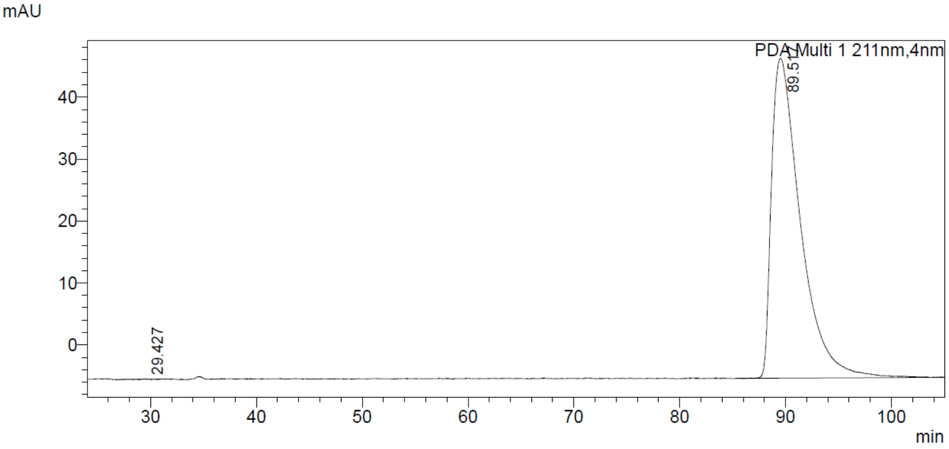

PDA Ch1 211nm

Peak#	Ret. Time	Area%
1	29.427	0.010
2	89.517	99.990
Total		100.000

HPLC Data for **43**: Chiralpak AD-H (90:10 hexane:IPA, flow rate 1.00 mL.min^{-1}, 211 nm, 30 °C) t_R (minor): 24.1 min, t_R (major): 12.9 min, >99:1 er.

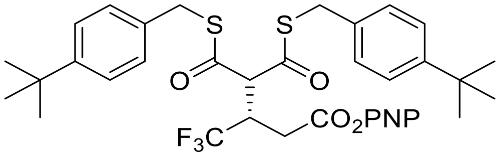

43

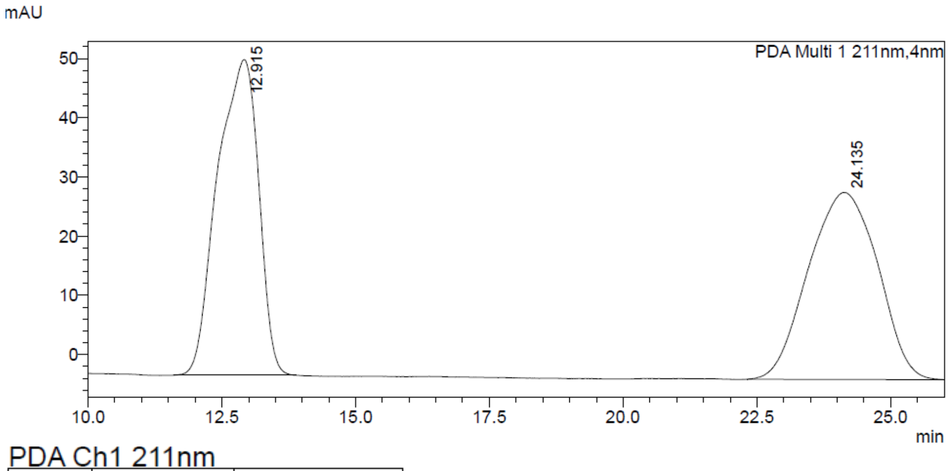

PDA Ch1 211nm

Peak#	Ret. Time	Area%
1	12.915	50.110
2	24.135	49.890
Total		100.000

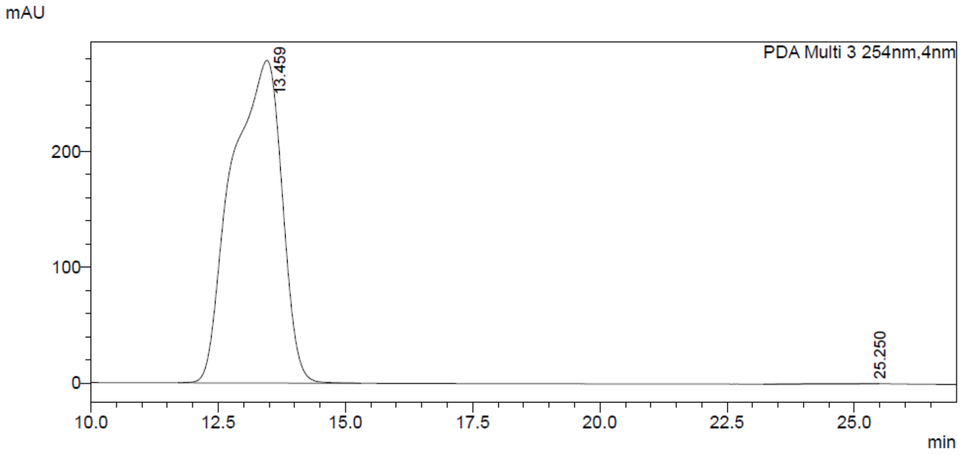

PDA Ch3 254nm

Peak#	Ret. Time	Area%
1	13.459	99.937
2	25.250	0.063
Total		100.000

HPLC Data for **44**: Chiralpak AD-H (90:10 hexane:IPA, flow rate 1.00 mL.min^{-1}, 211 nm, 30 °C) t_R (minor): 8.4 min, t_R (major): 7.7 min, >99:1 er.

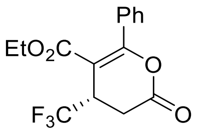

44

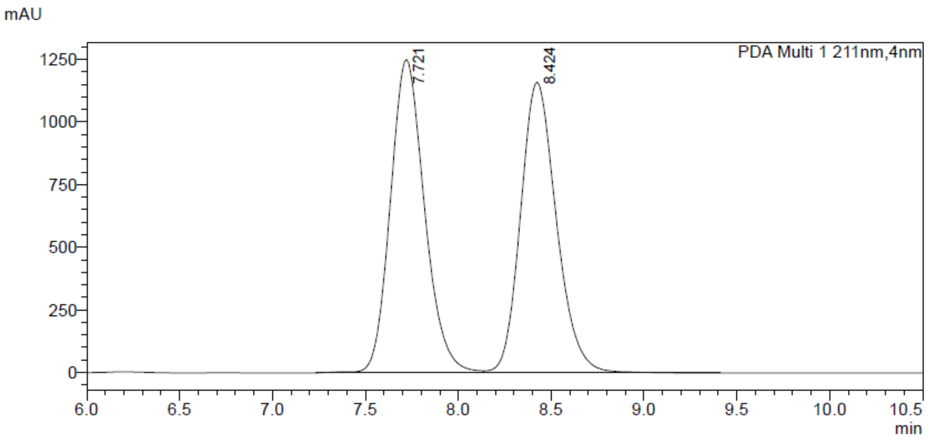

PDA Ch1 211nm

Peak#	Ret. Time	Area%
1	7.721	50.666
2	8.424	49.334
Total		100.000

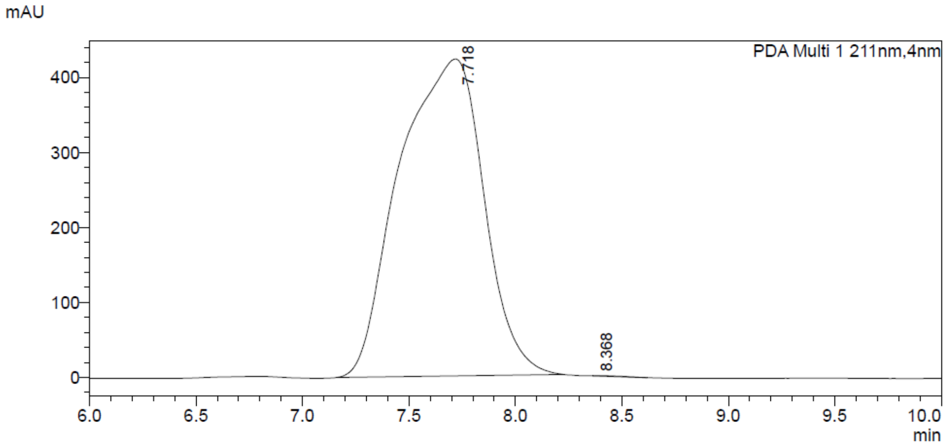

PDA Ch1 211nm

Peak#	Ret. Time	Area%
1	7.718	99.963
2	8.368	0.037
Total		100.000
